# Molecular Mechanisms of Temperature Tolerance Plasticity in an Arthropod

**DOI:** 10.1093/gbe/evae165

**Published:** 2024-07-26

**Authors:** Anne Aagaard, Jesper Bechsgaard, Jesper Givskov Sørensen, Tobias Sandfeld, Virginia Settepani, Tharina L Bird, Marie Braad Lund, Kirsten Gade Malmos, Kasper Falck-Rasmussen, Iulia Darolti, Kirstine Lykke Nielsen, Mogens Johannsen, Thomas Vosegaard, Tom Tregenza, Koen J F Verhoeven, Judith E Mank, Andreas Schramm, Trine Bilde

**Affiliations:** Section for Genetics, Ecology and Evolution, Centre for EcoGenetics, Department of Biology, Aarhus University, Aarhus C, Denmark; Section for Genetics, Ecology and Evolution, Centre for EcoGenetics, Department of Biology, Aarhus University, Aarhus C, Denmark; Section for Genetics, Ecology and Evolution, Centre for EcoGenetics, Department of Biology, Aarhus University, Aarhus C, Denmark; Section for Microbiology, Department of Biology, Aarhus University, Aarhus C, Denmark; Section for Genetics, Ecology and Evolution, Centre for EcoGenetics, Department of Biology, Aarhus University, Aarhus C, Denmark; General Entomology, DITSONG: National Museum of Natural History, Pretoria, South Africa; Department of Zoology and Entomology, University of Pretoria, Pretoria, South Africa; Department of Arachnology and Myriapodology, National Museum of Namibia, Windhoek, Namibia; Section for Microbiology, Department of Biology, Aarhus University, Aarhus C, Denmark; Interdisciplinary Nanoscience Center (iNANO), Aarhus University, Aarhus C, Denmark; Section for Genetics, Ecology and Evolution, Centre for EcoGenetics, Department of Biology, Aarhus University, Aarhus C, Denmark; Department of Zoology and Biodiversity Research Centre, University of British Columbia, Vancouver, British Columbia, Canada; Department of Ecology and Evolution, University of Lausanne, Lausanne, Switzerland; Department of Forensic Medicine, Aarhus University, Aarhus N, Denmark; Department of Forensic Medicine, Aarhus University, Aarhus N, Denmark; Interdisciplinary Nanoscience Center (iNANO), Aarhus University, Aarhus C, Denmark; Department of Chemistry, Aarhus University, Aarhus C, Denmark; Centre for Ecology and Conservation, University of Exeter, Penryn Campus, Penryn TR109FE, UK; Terrestrial Ecology Department, Netherlands Institute of Ecology (NIOO-KNAW), Wageningen 6708 PB, The Netherlands; Department of Zoology and Biodiversity Research Centre, University of British Columbia, Vancouver, British Columbia, Canada; Section for Microbiology, Department of Biology, Aarhus University, Aarhus C, Denmark; Section for Genetics, Ecology and Evolution, Centre for EcoGenetics, Department of Biology, Aarhus University, Aarhus C, Denmark; Centre for Ecology and Conservation, University of Exeter, Penryn Campus, Penryn TR109FE, UK

**Keywords:** temperature tolerance, transcriptomics, DNA methylation, metabolomics, phenotypic plasticity, population-specific plasticity

## Abstract

How species thrive in a wide range of environments is a major focus of evolutionary biology. For many species, limited genetic diversity or gene flow among habitats means that phenotypic plasticity must play an important role in their capacity to tolerate environmental heterogeneity and to colonize new habitats. However, we have a limited understanding of the molecular components that govern plasticity in ecologically relevant phenotypes. We examined this hypothesis in a spider species (*Stegodyphus dumicola*) with extremely low species-wide genetic diversity that nevertheless occupies a broad range of thermal environments. We determined phenotypic responses to temperature stress in individuals from four climatic zones using common garden acclimation experiments to disentangle phenotypic plasticity from genetic adaptations. Simultaneously, we created data sets on multiple molecular modalities: the genome, the transcriptome, the methylome, the metabolome, and the bacterial microbiome to determine associations with phenotypic responses. Analyses of phenotypic and molecular associations reveal that acclimation responses in the transcriptome and metabolome correlate with patterns of phenotypic plasticity in temperature tolerance. Surprisingly, genes whose expression seemed to be involved in plasticity in temperature tolerance were generally highly methylated contradicting the idea that DNA methylation stabilizes gene expression. This suggests that the function of DNA methylation in invertebrates varies not only among species but also among genes. The bacterial microbiome was stable across the acclimation period; combined with our previous demonstrations that the microbiome is temporally stable in wild populations, this is convincing evidence that the microbiome does not facilitate plasticity in temperature tolerance. Our results suggest that population-specific variation in temperature tolerance among acclimation temperatures appears to result from the evolution of plasticity in mainly gene expression.

**Significance**

Organisms can respond to changes in their environment by modifying their behavior or physiology. For example, in hot environments, animals could prioritize investment into a physiology that allows them to survive high temperatures. However, we have limited understanding of how these types of change are regulated. We investigated plasticity in responses to temperature stress in social spiders and found that spiders experiencing different prior temperature conditions were able to adjust their tolerances to extremely high and low temperatures. This tolerance was influenced by genes being switched on and off. Our findings help to understand how plasticity in gene expression contributes to modulate physiology and behavior to enable organisms to better cope with their environment.

## Introduction

Natural populations can respond to environmental variation by developing local adaptations or through phenotypic plasticity, and the relative importance of each in defining niche limits remains a key factor in determining how organisms respond to climatic changes. Evolutionary adaptations, based on standing genetic diversity or de novo mutations, typically require generations of selection to change phenotypes. This process may be too slow to allow populations to adapt to rapid environmental change, particularly in species with limited standing genetic variation and reduced efficacy of selection (Charlesworth 2009). Such species may be more likely to respond to environmental change by phenotypic plasticity.

Phenotypic plasticity allows organisms to rapidly and often reversibly respond to different environments. This strategy may be particularly advantageous for populations that live in heterogeneous or novel environments (DeWitt et al. 1998; Fox et al. 2019) and those that exhibit frequent extinctions and recolonizations (Hastings and Harrison 1994). However, there may be costs to plasticity which limit the flexibility it provides (Chevin and Hoffmann 2017; Gibert et al. 2019; van Heerwaarden and Kellermann 2020; Hangartner et al. 2022). Consequently, levels of plasticity are expected to be subject to selection (Pigliucci 2005). Evolutionary and plastic responses to the environment can therefore act both independently and complementarily to one other.

Temperature tolerance is a fitness-related phenotype with substantial variation in many species. In the context of climate change, temperature tolerance is a useful trait for testing the relative role of local adaptation and plasticity in shaping phenotypic variation. Moreover, there are many molecular mechanisms that have been associated with temperature tolerance, but the relative contribution of each to temperature tolerance phenotypes remains unclear. In ectotherms, responses to temperature are often initiated by gene regulation (Zhao et al. 2015; Clemson et al. 2016; Etges et al. 2017; Healy et al. 2017; Cahan et al. 2017; Metzger and Schulte 2018). Epigenetic modifications, specifically DNA methylation, may mediate such gene-regulating effects (Keller et al. 2016; Marshall et al. 2019). DNA methylation can be induced by environmental conditions, at least in some taxa (Dubin et al. 2015; Metzger and Schulte 2017). In this way, DNA methylation may alter the phenotype by modifying gene expression (Keller et al. 2016; Gatzmann et al. 2018; Kvist et al. 2018; Liu et al. 2019), thereby facilitating local responses to environmental change. The metabolome links genotypes to phenotypes (Fiehn 2002) and has the capacity to govern phenotypic responses to environmental conditions (Rohde et al. 2021). Metabolic products can facilitate cold tolerance (Koštál et al. 2001) and traits associated with cold tolerance (Overgaard et al. 2007; Colinet et al. 2012). Finally, adaptive functionality provided by the microbiome may provide a mechanism to enable the host to respond to novel environments (Burke et al. 2010; Shigenobu and Wilson 2011; Chevalier et al. 2015; Henry et al. 2019). Changes in microbiome composition can alter many phenotypes expressed by host organisms including their temperature tolerance (Dunbar et al. 2007; Chevalier et al. 2015; Raza et al. 2020).

The ability to respond by phenotypic plasticity to environmental change may be especially relevant for populations exposed to variable ecological conditions or those with restricted evolutionary potential. Both of these conditions apply to social spiders. The evolution of sociality in spiders is characterized by cooperative breeding, female-biased sex ratio, obligatory inbreeding, and female reproductive skew (Lubin and Bilde 2007; Settepani et al. 2017). These traits all contribute to reduce effective population size, which causes random loss of genetic diversity through drift and reduced efficacy of natural selection (Charlesworth 2009; Bechsgaard et al. 2019). Genetic data suggest that populations are not very long lived with frequent local extinctions, but with new colonizations established at sufficiently high rate to prevent species extinction (Settepani et al. 2014, 2017; Busck et al 2022). These meta-population dynamics reduce species-wide genetic diversity, since populations are frequently lost, removing lineage-specific variation. Existing populations are consequently not very genetically divergent but show distinct, albeit shallow, genetic differentiation (Settepani et al. 2014, 2017). Dispersal from natal sites to colonize new patches occurs over relatively long distances by mated females that fly using a sail of silk (termed ballooning) (Schneider et al. 2001). This implies that females and their offspring may colonize areas with climatic conditions that differ markedly from their natal environment. Indeed, social spider species are widely distributed and inhabit several climatic zones (Kraus and Kraus 1989; Majer et al. 2015), indicating that they are able to phenotypically respond to new and changing environments over relatively short timescales.

A first step toward the ultimate goal of understanding the mechanistic basis of a species’ capacity to thrive across a range of environments is to identify the relative contributions of different molecular modalities (i.e. genome, transcriptome, methylome, metabolome, and microbiome) to phenotypic variation. These modalities tend to be studied in isolation from one another (e.g. Lancaster et al. 2016; Metzger and Schulte 2017; Marshall et al. 2019; Raza et al. 2020; Rohde et al. 2021). Our aim in this study is to identify the modalities that contribute to shape plasticity in an ecologically relevant phenotype. Such insights may prove to be generalizable across species and also serve to identify future avenues for investigation. To this end, we performed a multiomic study to provide support for the role of different sources of variation (modalities) in modulating beneficial phenotypes in response to temperature acclimation in the social spider *Stegodyphus dumicola* Pocock, 1898 (Eresidae). We established a 42-d multiple common garden experiment with populations collected along a geographical temperature gradient. This allows us to examine the effects of population and temperature acclimation on heat and cold tolerance and their underlying sources of variation, including genome-wide genetic variation, epigenetic (DNA methylation), transcriptomic, metabolomic, and microbiome composition variation. Each of these molecular data sets represents different potential routes to variation in phenotype. This design enabled us to investigate the ability of a species known to have low molecular genetic variation and therefore likely low evolutionary potential to mount plastic phenotypic responses and to determine the relative importance of each modality in temperature tolerance.

## Results

### Temperature and Population Phylogeny

**Fig. 1.**
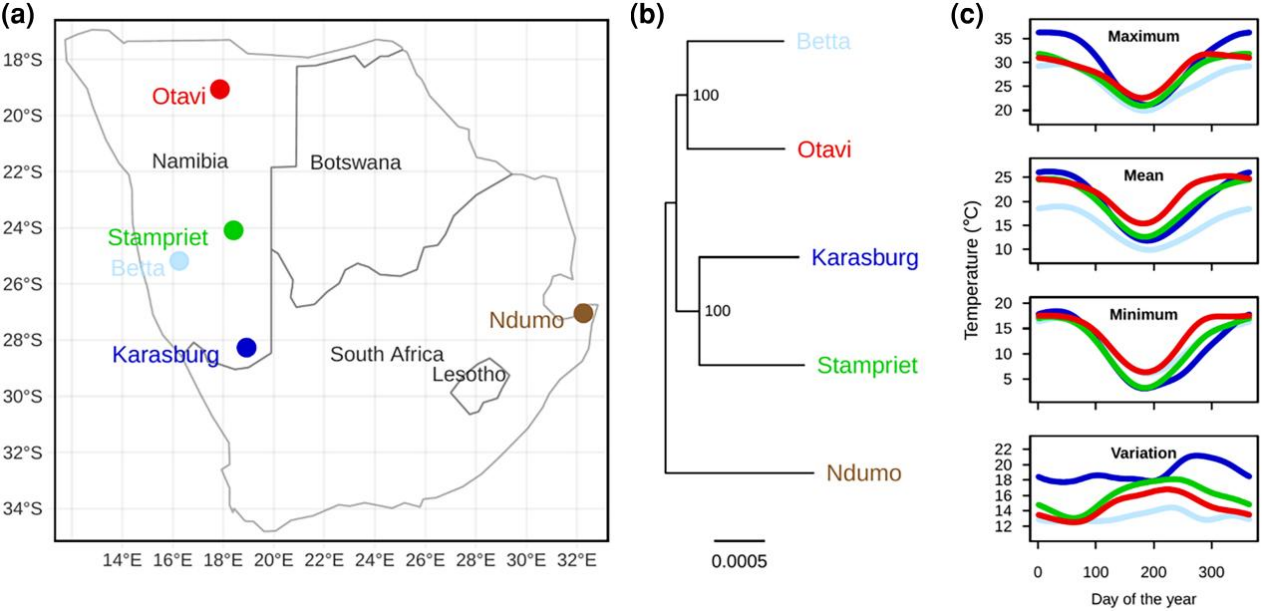
Geographical and phylogenetic information for the sampled *S. dumicola* social spider populations. a) Map of sampling sites (populations) in Namibia and the out-group Ndumo from South Africa. b) Phylogenetic relationship with bootstrap support above 80 on branch splits and the proportion of substitutions per site for branch length. c) Maximum, mean, and minimum temperature as well as variation in daily temperature over the course of a year (averaged across 30 years) at the sampling sites.

A phylogenetic reconstruction of the studied populations shows that Betta and Otavi are sister populations, as are Karasburg and Stampriet (Fig. 1b). These phylogenetic relationships do not map onto the geographical locations of the populations (Fig. 1a). Additionally, the most closely related populations do not share similar temperature conditions at each geographical location (Fig. 1c); for example, daily variation in temperature was more similar between Betta, Otavi, and Stampriet (Fig. 1c).

### Growth Rate and Survival

We acclimated spiders from each population to five temperatures in common gardens in the lab in order to assess growth and survival and to determine phenotypic responses in temperature tolerances and potential associated changes in gene expression, DNA methylation, metabolite profiles, and microbiome composition. Our data suggest that spiders from the four focal populations perform similarly in growth and survival under laboratory acclimation conditions independent of phylogenetic relationships. The best model for growth rate (full model: growth rate ∼ population ∗ temperature, *R*^2^ = 0.49, *F*_(7,296)_ = 40.66, *P* < 2.2e^−16^) shows strong effects of acclimation temperature on growth rate (*β* = 0.1, *P* < 2e^−16^; supplementary table S3, Supplementary Material online), but no overall effect of population or interaction (ANOVA; supplementary table S4 and fig. S2a, Supplementary Material online). The best model for survival was an additive model (family:binomial, logit link: survival ∼ population + temperature, AIC (Akaike Information Criterion) = 1665, *P* < 2e^−16^), with highly significant effects for both population and acclimation temperature (analysis of deviance; supplementary table S5, Supplementary Material online). The population effect was driven by Karasburg (*β* = 0.42, *P* = 5.2e^−05^; supplementary table S6, Supplementary Material online). Higher temperature acclimation (*β* = 0.11, *P* < 2e^−16^; supplementary table S6, Supplementary Material online) induced higher survival at higher temperatures (supplementary fig. S2b, Supplementary Material online).

### Temperature Tolerance

**Fig. 2.**
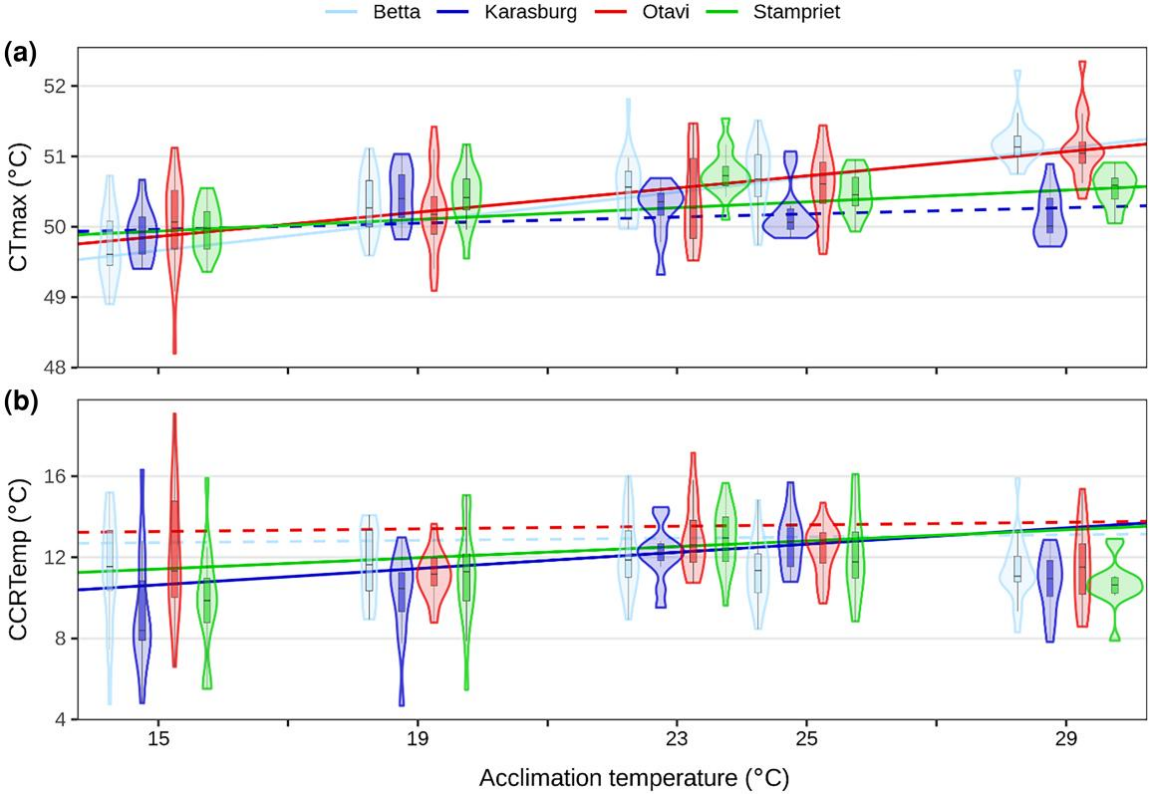
Violin plots of temperature tolerance assays as a function of acclimation temperature for four focal populations. a) Maximum temperature tolerance was measured as CTmax and shows population-specific acclimation capacities (slope of the trendline) of Betta = 0.1 °C/°C (CI: 0.084 to 0.124), Karasburg = 0.02 °C/°C (CI: −0.004 to 0.048), Otavi = 0.086 °C/°C (CI: 0.064 to 0.11), and Stampriet = 0.04 °C/°C (CI: 0.017 to 0.066). b) Cold tolerance was determined as CCRTemp and showed population-specific acclimation capacities of Betta = 0.028 °C/°C (CI: −0.065 to 0.12), Karasburg = 0.2 °C/°C (CI: 0.085 to 0.32), Otavi = 0.033 °C/°C (CI: −0.060 to 0.13), and Stampriet = 0.14 °C/°C (CI: 0.029 to 0.25). Solid lines have slopes significantly different from 0, while dashed lines do not (supplementary tables S7 and S8, Supplementary Material online). The extremities of violins reach the outliers of the boxplots. The *x*-values for the violins have been moved slightly around the acclimation temperature for easier interpretation of population-specific responses.

Spiders from the common temperature gardens were subsequently subjected to temperature tolerance tests, where both critical thermal maximum (CTmax) temperature and chill coma recovery temperature (CCRTemp) were determined. We identified differences among populations in how acclimation temperature affected temperature tolerance (i.e. population-specific plasticity): the best linear model for CTmax (linear dmodel: CTmax ∼ population ∗ temperature + body mass + time since feeding + preacclimation duration, *R*^2^ = 0.41, *F*_(10,298)_ = 20.61, *P* < 2.2e^−16^; supplementary table S9, Supplementary Material online) revealed an overall significant effect of both temperature acclimation (ANOVA; supplementary table S10, Supplementary Material online; *P* < 2.2e^−16^), population (*P* < 0.001), and interaction effects between the two (*P* = 1.3e^−07^). The population effect was driven by Karasburg where spiders showed a significantly lower CTmax compared with the other populations (*b* = −0.29, *P* = 0.001; supplementary table S9, Supplementary Material online). The model also revealed smaller but significant effects of body mass (ANOVA; *P* = 0.004; supplementary table S10, Supplementary Material online), time since feeding (*P* = 0.02), and preacclimation duration (*P* = 0.02; supplementary fig. S3, Supplementary Material online). All populations but Karasburg had slopes significantly different from 0 (supplementary table S7, Supplementary Material online). Betta and Otavi show similar patterns in slopes in CTmax (Fig. 2a), and both show steeper slopes, which differ statistically from those of Karasburg and Stampriet (supplementary table S11, Supplementary Material online).

The best linear model for CCRTemp (linear model: CCRTemp ∼ population ∗ temperature + body mass + time since feeding, *R*^2^ = 0.098, *F*_(9,320)_ = 3.86, *P* < 0.001; supplementary table S12, Supplementary Material online) revealed a significant effect of population (ANOVA; supplementary table S13, Supplementary Material online; *P* = 0.01), acclimation temperature (*P* = 0.02), time since feeding (*P* = 0.004; supplementary fig. S4, Supplementary Material online), and an interaction between population and acclimation temperature (*P* = 0.03). The population effect was driven by Karasburg having a significantly lower CCRTemp than the other populations (*β* = −0.83, *P* = 0.02; supplementary table S12, Supplementary Material online). Although all CCRTemp responses were not strictly linear for all populations, we apply linear models to facilitate biological interpretations of associated changes in phenotypic and molecular responses.

Despite the slopes/acclimation capacities not being significantly different from each other (Fig. 2; supplementary table S14, Supplementary Material online), slopes for Karasburg and Stampriet are significantly different from 0, as opposed to Betta and Otavi (supplementary table S8, Supplementary Material online). This created population patterns similar to those of CTmax: Betta and Otavi show a more similar response for CCRTemp as compared with the more similar response shared by Karasburg and Stampriet (Fig. 2). For both CTmax and CCRTemp, these patterns are in contrast to the population patterns for body mass, where Otavi spiders have the highest mass, Betta and Karasburg spiders show intermediate mass, while Stampriet spiders have the smallest body mass (supplementary fig. S5, Supplementary Material online), decoupling body mass as a primary explanatory variable for population patterns in CTmax and CCRTemp.

### Population and Acclimation Responses in Molecular Data Sets (Modalities)

**Fig. 3.**
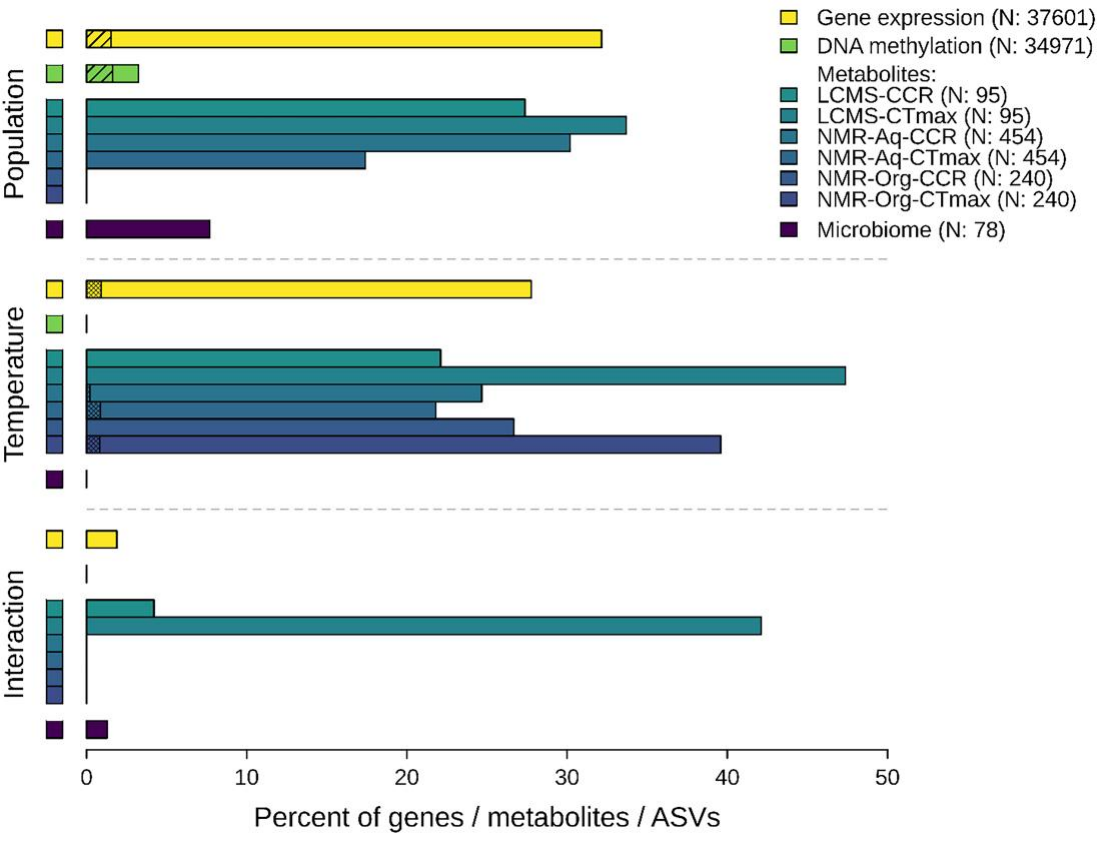
The percentage of genes (for gene expression and DNA methylation), metabolites, or ASVs (for microbiome) showing population and temperature responses as well as interaction between population and temperature. The total number of tested genes/metabolites/ASVs is indicated in parentheses in the legend. The hashed black lines indicate the percentage of genes with a population response in both DNA methylation and gene expression (total number of genes in overlap analysis: 34,971). The dense double hashing indicates the percent of genes or metabolites that may be involved in temperature tolerance phenotypes. DNA methylation data lacked power to test for interactions.

Gene expression and DNA methylation analyses were carried out on spiders from the five common temperature gardens, before spiders were subjected to temperature tolerance tests. We identified 12,089 differentially expressed genes based on population of origin (32%), 10,435 differentially expressed genes based on acclimation temperature (28%); 5,993 genes showed both population- and temperature-specific responses. Seven hundred eleven genes (1.9%) showed evidence of an interaction between population and acclimation temperature (Fig. 3; supplementary figs. S6a to S8, Supplementary Material online). DNA methylation of CpG, CHG, and CHH sites within gene bodies showed population-specific responses in 1,136 (Fig. 3; supplementary fig. S6b and S9, Supplementary Material online), 2, and 0 genes, respectively. No genes showed a methylation pattern consistent with an effect of acclimation temperature in either context.

A total of 572 genes showed a population response in both gene expression and DNA methylation (hashed bars, Fig. 3), which is very close to the number of genes expected to show such a response if there is no causal link between weighted methylation level (WML) and gene expression level $\left(\frac{N_{\mathrm{diff}.\;\mathrm{methy}.\;\mathrm{genes}}}{N_{\mathrm{all}.\mathrm{methy}.\;\mathrm{genes}}}\;*\;N_{\mathrm{diff}.\;\mathrm{expr}.\mathrm{genes}}\right)$. Correlations between WML and level of gene expression in the common genes that show a population response revealed a normal (or slightly bimodal) distribution of correlation coefficients (expectation if causal link: left skew; supplementary fig. S10, Supplementary Material online). This indicates that higher methylation level on its own does not lead to a higher level of gene expression. A slight depletion of correlation values around 0 (supplementary fig. S10, Supplementary Material online) might indicate the existence of a weak association between WML and gene expression; if so, however, the direction of this effect is inconsistent between genes. A subtle right skew on the histogram of correlations between standard deviation of expression and methylation level could indicate that higher methylation and stability of expression are correlated in some genes, but not consistent across the majority of genes (supplementary figs. S11 and S12, Supplementary Material online).

After spiders had been acclimated and assayed for temperature tolerances, samples were collected for metabolite analyses using two methods: Liquid chromatography–mass spectrometry (LC-MS) and nuclear magnetic resonance (NMR) spectroscopy. In the LC-MS metabolite data set, we retrieved 4,188 features using positive ionization, of which 21 metabolites could be identified from authentic standards or MS spectral database entries. Using negative ionizations, 3,215 features were found, resulting in 74 named metabolites. We chose to focus only on named metabolites for further analysis and retained 95 named metabolites. For spiders having gone through CTmax treatment, the intensity of 32 metabolites (34%) showed a population response, 45 metabolites (47%) were influenced by acclimation temperature, 15 showed an effect of both, and 40 metabolites (42%) showed interaction effects between population and acclimation temperature (Fig. 3 and supplementary fig. S13a, Supplementary Material online, visualization in supplementary fig. S16, Supplementary Material online). There was little differential clustering of populations of LC-MS metabolites on a principal component analysis (PCA), but a slight tendency for Karasburg and Stampriet spiders to separate from Betta and Otavi spiders (supplementary fig. S14, Supplementary Material online), while Karasburg spiders were more separated on a partial least squares discriminant analysis (PLS-DA) (supplementary fig. S15d, Supplementary Material online). LC-MS metabolites with acclimation temperature effect separated clearly in PLS-DA space (supplementary fig. S15e, Supplementary Material online).

Spiders showed clear metabolomic signals of heat stress after CTmax treatment (LC-MS; supplementary table S2 and fig. S16, Supplementary Material online), including changes in amino acid abundance, likely resulting from protein degradation, intermediates from the citric acid cycle, and glycolysis. Nucleosides/nucleotides and their degradation products, indicating ATP breakdown and disruption of homeostasis, were also identified (supplementary table S2, Supplementary Material online). In addition, we saw responses in the intensity of osmolytes and antioxidants, indicating osmotic challenges (supplementary table S2, Supplementary Material online). Collectively, these metabolites indicate heat stress in the spiders. Several of the same metabolites were also identified in the warmest natural population of *S. dumicola* (Sandfeld et al. 2022), suggesting that our laboratory conditions likely induced metabolic changes consistent with temperature-induced stress responses in wild populations.

For spiders subject to CCRTemp treatment, intensities of 26 metabolites (27%) showed a population-specific response, 21 (22%) responded to acclimation temperature, seven showed an effect of both population and temperature, while four metabolites (4%) showed interaction effects (Fig. 3 and supplementary fig. S13b, Supplementary Material online, visualization in supplementary fig. S19, Supplementary Material online). Multivariate analyses on metabolites with population effect tended to separate Karasburg and Stampriet from Betta and Otavi (PCA, supplementary fig. S17, Supplementary Material online; PLS-DA, supplementary fig. S18d, Supplementary Material online). Metabolic groups with many representatives for CCRTemp treatment included amino acids, sugars, nucleoside, and nucleoside precursors, indicating breakdown of proteins and nucleosides (supplementary table S2 and fig. S19, Supplementary Material online). We also saw indications of impaired redox homeostasis and oxidative stress and the fermentation product lactate (M89T71_neg, supplementary fig. S19, Supplementary Material online), indicating shortage of oxygen and thus a transition from respiration to fermentation. Some sugars and polyols also responded after CCRTemp treatment, indicating that thermo-protection against cold could have been induced.

NMR spectra of methanol/water extractions of metabolites showed 454 NMR peaks each of which was tested separately using ANOVA (aov in R) for effects of population, acclimation temperature, and their interaction. For spiders subjected to CTmax treatment, 79 NMR peaks (17%) showed a population effect, 99 (22%) showed an acclimation temperature effect, and 43 showed an effect of both population and acclimation temperature, while no interaction effect was identified (Fig. 3; supplementary figs. S13c, S15a and b, and S20, Supplementary Material online). For spiders subjected to CCRTemp, 137 NMR peaks (30%) showed a population effect, 112 (25%) showed an effect of acclimation temperature, and 33 showed both population and acclimation effects, while no interaction effect was found (Fig. 3; supplementary figs. S13d, S18a and b, and S21, Supplementary Material online).

The NMR spectra of organic extracts of metabolites revealed 240 peaks. CTmax spiders showed 95 peaks (40%) with effect of acclimation temperature (Fig. 3; supplementary figs. S13c, S15c, and S32a and c, Supplementary Material online), while CCRTemp spiders revealed 64 peaks (27%) with acclimation temperature effect (Fig. 3; supplementary figs. S13d, S18c, and S32b and d, Supplementary Material online). Organic extractions target hydrophobic metabolites such as compounds involved in membrane fluidity and cuticle wax layers. Most noticeable, no effect of population was found on peaks for neither CTmax- nor CCRTemp-treated spiders when analyzing organic extracts, indicating that membrane compounds primarily are responsive to acclimation temperatures.

The bacterial microbiome composition was investigated using 16S sequencing for spiders from the common temperature gardens, before spiders were subjected to tolerance tests. The bacterial microbiome showed negligible temperature acclimation responses (Fig. 3). Following filtering, 78 amplicon sequence variants (ASVs) were retained, six of which showed a population effect in relative abundance (8%), and only one ASV showed an interaction effect with acclimation temperature (1%; Fig. 3 and supplementary fig. S22, Supplementary Material online). The taxonomic identities of ASVs showing responses were as follows: *Mycoplasma* (ASV1), *Candidatus Arachnospira* (formerly classified as *Borrelia*, ASV12), *Diplorickettsia* (ASV2), *Weeksellaceae* (ASV4, ASV7), and *Rickettsiella* (ASV6, Interaction).

### Modalities Hypothesized to Govern Temperature Tolerance Plasticity

Temperature acclimation-induced patterns of variation in the transcriptome and the metabolome that resemble the patterns identified in phenotypic temperature tolerances, consistent with a direct involvement of these modalities in shaping plasticity in temperature response (interaction, Fig. 3). The expression of 270 out of 10,435 genes (2.6%) showed a temperature acclimation response and population-specific slopes with responses similar to CTmax (supplementary fig. S23, Supplementary Material online), and 65 out of 10,435 genes (0.6%) showed responses similar to that of CCRTemp (supplementary fig. S24, Supplementary Material online). These candidate genes for heat and cold tolerance responses showed varied functional annotations (supplementary table S15, Supplementary Material online). To add confidence to the similarity analyses, we estimated the number of false positives by rerunning the analyses on permuted gene expression and metabolite intensity data. The number of estimated false-positive genes for CTmax and CCRTemp patterns was 0 and 2, respectively. Surprisingly, the genes that showed similar expression responses to acclimation as the phenotypic temperature tolerances (330 genes in total) were much more methylated compared both with the remaining genes that showed an expression response to temperature acclimation and with the genes that did not show an expression response to temperature acclimation (supplementary fig. S33, Supplementary Material online).

Out of 239 metabolites with acclimation temperature effect after CTmax treatment, the intensities of six (2.5%) shared responses similar to that of CTmax (supplementary figs. S25 and S26, Supplementary Material online). One out of 197 metabolites discovered for CCRTemp-treated spiders showed a response similar to that of CCRTemp (0.5%, NMR, aqueous extraction, supplementary fig. S27, Supplementary Material online). The number of estimated false-positive metabolites for CTmax and CCRTemp patterns was 0 and 3, respectively.

## Discussion

### Phenotypic Plasticity in Temperature Tolerances

Our common garden-rearing design revealed that both heat and cold tolerance phenotypes were plastic. Reaction norms varied significantly among populations, revealing substantial variation among populations in temperature tolerance plasticity. The capacity to mount plastic phenotypic responses is expected to be exposed to evolutionary forces (DeWitt et al. 1998; Lande 2009; Chevin and Hoffmann 2017). However, while numerous examples of adaptive population differences in plasticity at the level of gene expression exist (e.g. Lancaster et al. 2016; Gibbons et al. 2017; Swaegers et al. 2020), there is scarce evidence for adaptive population differences in phenotypic plasticity in responses to environmental stress in functional traits at the organismal level and in temperature tolerance traits specifically (Gunderson and Stillman 2015; Sørensen et al. 2016). Our study, therefore, provides a novel example of population-specific plasticity in modulating temperature tolerance phenotypes.

Interestingly, the observed temperature tolerances were not necessarily associated with the climatic conditions from the collection locations. For example, individuals from the on average warmest (Otavi) and coldest populations (Betta) showed similar temperature tolerances. There was evidence consistent with adaptive responses to cold temperatures, as individuals from populations that experience the lowest temperatures during winter (Karasburg and Stampriet) also expressed the highest cold tolerance and showed plasticity in cold tolerance (Figs. 1c and 2b). However, heat tolerance was not shaped by maximum temperatures, as individuals from Karasburg, which experienced the highest temperatures during summer, showed the lowest heat tolerance and no plasticity in heat tolerance (Fig. 2).

The patterns of heat and cold tolerance indicate at least a partial decoupling of temperature tolerances from the temperature conditions at the sampling location. Otavi/Betta and Stampriet/Karasburg each represent sister populations (Fig. 1b); it is, therefore, possible that the observed variation results from phylogenetic inertia if local adaptation in plasticity constrains evolutionary responses to a new environment (supplementary fig. S28, Supplementary Material online) (Blomberg and Garland 2002; Cooper et al. 2010). Local adaptation to any one thermal regime can be hampered by several factors including long-distance dispersal, the lack of adaptive potential, or developmental or genetic constraints on plasticity (Chevin and Hoffmann 2017; van Heerwaarden and Kellermann 2020; Hangartner et al. 2022). Social spider lineages are propagated by long-distance dispersal (Schneider et al. 2001), which could lead to continual colonialization of different thermal environments exerting strong selection on the capacity to mount plastic responses in new environments (Ghalambor et al. 2007; Mallard et al. 2020). However, populations harbor low genetic diversity, which reduces adaptive potential particularly over short time frames thereby likely preventing continuous local adaptation (Settepani et al. 2017). This substantiates the argument that there should be selection for the general capacity to mount plastic responses to local temperature conditions.

### Modalities Hypothesized to Govern Temperature Tolerance Plasticity

We analyzed multiple molecular data sets to identify modalities that likely affect the plastic responses in temperature tolerances. We observed evidence of temperature acclimation-induced changes in the transcriptome and the metabolome matching the reaction norms in phenotypic temperature tolerances. Of all genes, 31% showed plasticity in gene expression in response to temperature acclimation (Fig. 3), indicating a substantial transcriptomic response to ambient temperature. Expression profiles of 274 plastically expressed genes (2%) showed similar population and acclimation responses to heat and cold tolerance (supplementary fig. S23), linking regulation of gene expression to phenotypic plasticity in temperature tolerances. These results suggest that the evolution of plasticity in gene expression explains much of the observed variation in temperature tolerance plasticity. This provides an important example of the evolution of plasticity in gene expression in wild populations, as empirical evidence mainly comes from experimental studies using model organisms (e.g. Gibbons et al. 2017; Mallard et al. 2020; but see Lancaster et al. 2016; Swaegers et al. 2020).

Interestingly, changes in gene expression in response to temperature acclimation did not appear to be directly regulated by DNA methylation, as we identified no changes in the methylome in response to acclimation temperature (Fig. 3; supplementary fig. S6, Supplementary Material online). This result shows that induced changes in methylation in response to acclimation do not occur over short time scales (6 weeks in common gardens) in this system. Within invertebrates, the relationship between gene body methylation and gene expression is unclear, but higher methylation has been proposed to stabilize gene expression (e.g. Gatzmann et al. 2018), a pattern we also find for a subset of genes (supplementary figs. S11 and S12, Supplementary Material online). Gene function has been proposed to play a role, such that plastic genes should be less methylated. However, we found plastic genes to be either highly methylated or lowly methylated (bimodal), while genes likely to be involved in temperature tolerances were generally highly methylated (supplementary fig. S33, Supplementary Material online). Thus, the effects of methylation on gene expression vary across genes in our study, emphasizing that the effects of DNA methylation on gene expression vary substantially among invertebrates (Hunt et al. 2010; Sarda et al. 2012; Gavery and Roberts 2014; Dimond and Roberts 2016, 2020; Gatzmann et al. 2018; see also Duncan et al. 2022).

Individual metabolic profiles can tightly link genotypes to physiological phenotypes, either through amino acid composition in protein-coding genes or gene expression (Rohde et al. 2021). We identified substantial responses in metabolome composition as a function of temperature acclimation (Fig. 3; supplementary fig. S13, Supplementary Material online), with seven metabolites showing association with temperature tolerance phenotypes (2.5% for CTmax and 0.5% for CCRTemp; supplementary figs. S15 to S27, Supplementary Material online). This pattern suggests either a function of these metabolites in temperature tolerance plasticity, or that they are produced as consequence of heat or cold stress. Interestingly, metabolites involved in cellular membrane and cuticle wax layer composition, which influence membrane melting points and thereby temperature tolerance (Malmos et al. 2021), showed plastic but no population responses (Fig. 3; supplementary fig. S13, Supplementary Material online). This is consistent with a role of plastically induced membrane-related metabolites in shaping temperature tolerance through their effect on cellular function. In particular, the warmest acclimation temperature-induced changes in hydrophobic metabolite profiles that may facilitate heat tolerance (supplementary figs. S15c and S32a and c, Supplementary Material online), and similarly, the extreme temperature treatments mediated changes in metabolite profiles that may influence cold tolerance (supplementary figs. S18c and S32b and d, Supplementary Material online). Malmos et al. (2021) found functional support for metabolomic influence on cuticle membrane fluidity of spiders in response to temperature change, consistent with adaptive modification of the membrane melting temperature. Our results corroborate the functional role of metabolites in shaping phenotypic plasticity in temperature tolerance.

We found no acclimation response in microbiome composition, indicating that variation in the microbiome was not associated with plasticity in temperature tolerance phenotypes. This is notable, as host–microbiome interactions are hypothesized to aid environment-specific survival of hosts (Houwenhuyse et al. 2021) and colonization of novel environments (Henry et al. 2013). Instead of temperature-driven changes in host microbiome, we found population-specific variation in microbiome composition (Fig. 3; supplementary fig. S22, Supplementary Material online, driven primarily by the Karasburg population, supplementary figs. S29 to S30, Supplementary Material online). This is consistent with previous reports of consistent microbiome compositions within and among populations (Busck et al. 2020, 2022; Sandfeld et al. 2022; Rose et al. 2023). A previous environment association study identified correlations in microbiome composition with local humidity (Aagaard et al. 2022). While it is possible that population-specific host–symbiont associations facilitate host responses to humidity, we need more information on the drivers of host–symbiont associations to distinguish environmental determinants of host–symbiont compositions (e.g. Rose et al. 2023) from host–symbiont facilitation of adaptive host responses.

A significant number of variants from transcriptome, metabolome, methylome, and microbiome data sets retained population differences after more than 6 weeks in a common-garden setup, suggesting a role in shaping population differences (Fig. 3). The environment association study by Aagaard et al. (2022) on the same spider species identified variation in DNA methylation level in several thousand genes that showed strong correlations with temperature-related climate parameters. As we did not detect an acclimation response in gene methylation levels in the present study, the combined data sets suggest a role of population-specific methylations in shaping other responses to temperature variation than the two temperature tolerance traits measured here. This may also apply to population-specific differences in metabolic profiles. We found population-specific metabolomic differences in the levels of glycine, leucine, phosphorylated sugars, and glycerol after cold treatment, which have been shown to adaptively respond to cold treatment in other invertebrates (Overgaard et al. 2007; Michaud et al. 2008; Colinet et al. 2012; Slotsbo et al. 2012; Vesala et al. 2012). Observations of interaction effects on expressed metabolites (seen in the lower part of Fig. 3) provide a complimentary example of the genotype-by-environment interaction pattern seen in Fig. 2. Further work is required to identify the mechanistic basis of the interactions. We might expect some of them to be visible within the traits that we have quantified, but it is also possible that downstream interactions between gene expression products and the environment could be the mechanistic basis for our observed phenotypic GxE effects.

Understanding the mechanisms through which environmental variation impinges on the phenotype is important for predicting the ability of populations to keep pace with environmental change and to colonize new environments. We find that patterns of plastic and reversible changes in the phenotype of *S. dumicola* may be governed by variation in specific molecular modalities, i.e. the transcriptome and metabolome, while other modalities may be utilized to shape the phenotype across populations. The acclimation capacities for heat tolerance documented here are of a magnitude similar to other arthropods (Jumbam et al. 2008; Gunderson and Stillman 2015; Sørensen et al. 2016; Anthony et al. 2021). This acclimation capacity in temperature tolerance may be inadequate to enable organisms to cope with exposure to extreme temperatures (Fig. 2). Multiple physiological and behavioral responses acting in concert, as for example behavioral thermoregulation and cuticle melting point alterations (Gunderson and Stillman 2015; Sgrò et al. 2016; te Pas et al. 2017; Malmos et al. 2021; Rohde et al. 2021) may be required to enable organisms to cope with large environmental variation.

## Materials and Methods

### Sample Collection and Temperature Conditions

We collected *S. dumicola* spiders from four different geographical regions in Namibia: Betta, Karasburg, Otavi, and Stampriet in April 2017 (Fig. 1a). Approximately 3,000 individual spiders were sampled from each of these four populations (in total c. 12,000 individuals) and brought to the laboratory at Aarhus University. Temperature data from the four collection sites were extracted from a 30-year mean data set (Aagaard et al. 2022) (Fig. 1c). Phenotypic responses such as behavioral thermoregulation and cuticle melting point alterations in response to temperature changes have previously been demonstrated in this species (Malmos et al. 2021).

### Population Phylogenetic Reconstruction

To construct the phylogenetic relationship among populations, we used genomic data from spiders sampled in the same focal populations (Betta, Karasburg, Otavi, and Stampriet) as published in Aagaard et al. (2022) including the out-group from Ndumo, South Africa. We have monitored these populations continuously over several years (2017 to 2021), and previous studies show that individuals sampled within a population are genetically highly similar (Settepani et al. 2017). We used Bcftools to construct vcf-files (“mpileup” without indel calling (-I) and “call”; Li 2011), Samtools faidx (Li et al. 2009) to extract coding positions, and bcftools “consensus” (Danecek and McCarthy 2017) to call consensus sequences, subsequently concatenating them to one sequence per location and then aligning them. Every 50th exon was extracted, and a neighbor-joining tree was built by Mega-X (Kumar et al. 2018). The length of the aligned sequences was ∼1.5 Mb, and 1,000 bootstraps were run to support branching.

### Common Garden Acclimation

Upon arriving at the laboratory, all spiders were kept at room temperature (21 °C) for at least 2 weeks before being allocated to different acclimation treatments. Spiders were acclimated at five constant thermal regimes 15, 19, 23, 25, and 29 °C for 42 d prior to testing their thermal tolerances. We chose common garden temperature regimes based on temperature profiles collected in natural spider nests in several wild populations (Busck et al. 2020; Malmos et al. 2021). We decided to include temperatures that represent the lower range (15 °C) as well as the higher range (29 °C, based on averages) to challenge the spiders both at low and high temperatures in the common gardens. All thermal regimes had a 12-h/12-h light/dark photoperiod. To allocate the spiders to the five thermal acclimation regimes, 150 individuals taken from each communal nest were divided among five plastic boxes (10 × 10 × 15 cm) (hereafter referred to as nest boxes) with two sides replaced with mesh to allow airflow. In populations where we had <20 communal nests available, spiders from two or three nests were mixed in a nest box. We had 13 to 23 replicates (nest boxes) per population/acclimation group (supplementary table S1, Supplementary Material online), in total 371 nest boxes containing 11,130 spiders. Due to limitations on the number of spiders that could be included in each batch for thermal tolerance testing (∼200 individuals per batch), and to minimize both body size differences among spiders and block effects, the setup of nests was staggered so each day, and two communal nests from any one of the populations were distributed across the five acclimation treatments. This was done each weekday for 8 weeks. The order of nests was determined by selecting the two nests with the largest spiders, as evaluated by eye. During the following 42-d acclimation period, all spiders were sprayed with water three times per week and fed houseflies, crickets, mealworms, grasshoppers, or cockroaches twice per week. The common garden design and analyses aimed to disentangle adaptive and plastic responses in temperature tolerances. We are aware that we cannot rule out potential effects of developmental plasticity as individuals were not kept in common gardens for multiple generations. This is not possible with this species, which has a 1-year development time.

### Growth Rate and Survival

To assess spider survival and growth, live spiders were counted and weighed three times during the acclimation period. Growth rate was calculated as current body mass/initial body mass. Survival was estimated as the number of survived spiders after acclimation relative to live spiders in each box at the beginning of acclimation treatments. Statistical analyses were done in R v. 3.6.3 (R Core Team 2020), using lm() to model growth rate and glm() with binomial family and logit link to model survival data. Best models were found using the step() function in R (base stats package), based on AIC values.

### Thermal Tolerance

After thermal acclimation, we estimated CTmax and CCRTemp as measures of heat and cold tolerances, respectively. Twenty individuals from each nest box and acclimation temperature combination (∼400 individuals from each population and acclimation temperature and ∼8,000 individuals in total) were weighed separately prior to placement in 5-mL glass vials with watertight lids, divided randomly between the two assays, and attached to racks that were submerged into a water-filled aquarium (more details below).

### CTmax

Water temperature was adjusted to 25 °C prior to the experiment and increased at a rate of 0.1 °C/min immediately after submerging the racks, while stirring the water with a pump to ensure consistent water temperature surrounding all vials. Four cameras were used, each recording 25 spiders until the water had reached 55 °C. The footage was subsequently manually inspected to identify the time at which each individual spider ceased moving. The time point was converted to a temperature (the CTmax estimate) using the equation from the average standard curve based on temperatures recorded during several CTmax assays (supplementary fig. S1, Supplementary Material online).

### CCRTemp

Ethylene glycol was added to the aquarium before the CCRTemp experiment to prevent freezing. The temperature of the water and ethylene glycol mixture was adjusted to 0 °C prior to the start of the experiment. The rack was submerged and the temperature kept at 0 °C for 150 min, before increasing the temperature at 0.5 °C per min. Keeping the spiders at 0 °C for 150 min causes the spiders to enter chill coma, a reversible physiological state preventing movement (Findsen et al. 2014). Four cameras were used to each record 25 spiders until the water reached 25 °C. The videos were subsequently manually inspected to identify the time that each individual spider initiated coordinated movement. This time point was converted to a temperature (the CCRTemp estimate) using the standard curve based on average temperatures recorded during several CCRTemp assays (supplementary fig. S1, Supplementary Material online).

The temperatures used in assays for CTmax and CCRTemp, respectively, are ecologically relevant for *S. dumicola* spiders, as temperatures can reach 55 °C within nests and fall below 0 °C during the night in their natural habitats (Malmos et al. 2021). Spiders are, therefore, likely to both be exposed to temperatures that challenge their heat tolerance and to enter chill coma (see below).

### Thermal Tolerance Analyses

All analyses were performed using R v. 3.6.3 (R Core Team 2020). We calculated means of CTmax and CCRTemp for each nest box (population/acclimation replicate), and these were used as data points in subsequent statistical models. Linear models of CTmax and CCRTemp were set up with lm(), and the effects of population, acclimation temperature, and interactions were tested with ANOVA using the anova() function. The analyses also included spider weight (body mass), number of days since last feeding (time since feeding), and number of days spiders were kept at room temperature in the lab, before they entered acclimation conditions (preacclimation duration). The step() function (R base stats package) was used to determine the best model, based on AIC value. The slopes of the linear models were tested for significant differences between slopes and for difference to 0 using the emtrends() and cld() functions from the R emmeans and MultComp packages, respectively.

### Gene Expression (Transcriptome)

#### RNA Extraction and Sequencing

Extraction of RNA was done on ten spiders (one from each of the ten nest boxes) per population/acclimation temperature group (200 individuals in total), after 42 d of thermal acclimation. RNA-extracted spiders had not been subjected to temperature tolerance tests. Spiders were flash frozen in liquid nitrogen in preparation for RNA extraction. The extraction was done using QIAGEN RNeasy Mini Kit (Qiagen, Hilden, Germany); 199/200 extractions were successful and constructed libraries were sequenced with 150-bp paired-end on Illumina HiSeq2500. Further extraction and sequencing details are described in Liu et al. (2019).

#### Gene Expression Analyses

RNA-sequencing reads were analyzed following a protocol from Pertea et al. (2016), with specific parameters described earlier in Liu et al. (2019). In short, we used FastQC v. 0.11.5 (FastQC 2016) and trimmomatic v. 0.39 (Bolger et al. 2014) for quality check and trimming, HISAT2 v. 2.1.0 (Kim et al. 2015) for mapping to the *S. dumicola* genome (Liu et al 2019), and stringtie v. 2.1.1 (Pertea et al. 2015) for reference-guided transcriptome assembly and quantification. Subsequent analyses were done on the level of genes. We then used DEseq2 v. 1.26 (Love et al. 2014) for differential expression analysis, excluding gene parts (gp), estimating effects of population and acclimation temperature and interactions using likelihood ratio tests (false discovery rate [fdr] < 0.05) in the DEseq function. Minimum expression filtration was done automatically by DEseq2 results() function.

Since we extracted whole spiders, we note that differences in gene expression may also be caused by changes in tissue composition in response to temperature treatments (Montgomery and Mank 2016; Darolti and Mank 2023), although this should be relatively minor. Since DEseq2 does not allow a log fold change threshold when using likelihood ratio tests on multiple levels, we minimize this issue by applying strict fdr thresholds.

### DNA Methylation (Methylome)

#### DNA Extraction and Bisulfite Sequencing

From each of the ten nest boxes from the population/acclimation combinations, one spider (200 individuals in total) was placed in a −80 °C freezer after 42 d of thermal acclimation (before temperature tolerance tests were initiated). DNA was extracted from each spider separately using the QIAGEN DNeasy Blood and Tissue Kit (Qiagen, Hilden, Germany). The extracted DNA from all individuals from the same population and acclimation temperature was pooled (DNA from ten spiders/pool) in equal concentrations resulting in 20 pools in total (four populations × five acclimation temperatures) before bisulfite conversion. Paired-end sequencing (150 bp) was performed on an Illumina HiSeq2500 platform. λDNA was used as a control for the bisulfite conversion rate, and more than 99% of the unmethylated cytosines were converted. Library construction and sequencing were performed by Novogene Ltd. (Hong Kong).

#### DNA Methylation Analyses

We used FastQC v. 0.11.5 (FastQC 2016) and Trim Galore v. 0.4.1 (Trim Galore 2015) to check and trim bisulfite reads, before mapping them (“bismark”) to the prepared *S. dumicola* reference genome (“bismark_genome_preparation”) (Liu et al. 2019), using Bismark v. 0.19.0 (Krueger and Andrews 2011). Bismark was also used to remove PCR duplicates (“deduplicate_bismark”) and to extract methylated reads per cytosine (“bismark_methylation_extractor”). Deduplication removed ∼15% of the reads that mapped in each sample. Exact details on trimming and parameters used can be found in Liu et al. (2019). We filtered for coverage of cytosines between 10× and 32×, the high-end threshold determined as the top 1% of the coverage distribution (supplementary fig. S31, Supplementary Material online). Cytosine methylation in CpG (CG), CHG, and CHH contexts (H = C, T, or A) was investigated separately. To measure methylation in gene bodies, we calculated WML (sum_region_[C_Meth_]/sum_region_[C_meth_ + C_unmeth_]) within each gene (Schultz et al. 2012). To test whether genes were differentially methylated with respect to the main effects (population and acclimation temperature), we used DSS v. 2.34.0 (Park and Wu, 2016), which employs a beta-binomial regression model with arcsine link. In DSS, we tested the main effects of population and acclimation temperature using the DML.test function (Wald test), keeping genes with a fdr < 0.05. In all subsequent analyses, only CpG methylation was used.

### Metabolites (Metabolome)

#### Metabolite Extractions

Five spiders were collected and pooled from each of 3 to 7 nest boxes for each of the 20 population/acclimation combinations after they had been subjected to temperature tolerance tests, in total 112 samples for CCRTemp and 102 for CTmax (1,070 individuals in total). The pooled samples were frozen and stored at −80 °C. Frozen spider samples were used for metabolite extractions with methanol/water for LC-MS and NMR analyses, prepared as in Sandfeld et al. (2022). The residual spider and matrix were used for extraction with organic solvents.

Frozen spider sample (100 mg) was extracted twice with 4-mL cold methanol (80%) in TeenPrep Lysing Matrix E tubes (MP Biomedicals, USA) on a FastPrep-24 5G Homogenizer (MP Biomedicals, USA), using a custom program (4.5 m/s, 20 s, custom) and then centrifuged for 5 min (13,000 rpm, 4 °C), and the supernatant was transferred to freeze-dry tubes. The residual pellet was then extracted twice with 4-mL high-performance liquid chromatography-grade water as described above, and the supernatant was added to the methanol fraction, flash frozen in liquid nitrogen and freeze-dried using a MicroModulyo Freeze Dryer (Thermo Fisher Scientific, USA) coupled to a Chemistry-HYBRID RC 6 vacuum pump (Vacuubrand GmbH, Germany), and stored at −80 °C until processing. Approximately 1 mg was used for LC-MS analysis, while the remainder was used for ^1^H NMR analysis. The residual spider and matrix pellet were used for the organic/hydrophobic extractions with 1-mL methanol and 5-mL CHCl_3_, at room temperature for 5 min. The soluble hydrophobic fraction was transferred to a glass vial and dried under air flow at room temperature.

#### NMR Spectroscopy

The lyophilized aqueous metabolite extracts were dissolved in 700-µL D_2_O containing 20 mM sodium phosphate buffer and 0.08 mM sodium trimethylsilylpropanesulfonate (DSS). The dissolved sample was centrifuged for 10 min at 4,248 × *g*, and the supernatant was transferred to 5 mm SampleJet NMR tubes using glass Pasteur pipettes. The lyophilized organic metabolites were dissolved in 700-µL CDCl_3_ and transferred to 5-mm Samplejet tubes.

^1^H NMR 1D experiments were recorded on a Bruker 500-MHz spectrometer equipped with a Bruker Avance-II console, a 5-mm triple resonance probe, and an automatic sample changer (SampleJet, Bruker). For all samples, automatic tuning, locking, and shimming were applied, and all experiments were performed at 295.0 K. Experiments for the aqueous samples were recorded using the standard CPMG pulse sequence named cpmgpr1d with 64 scans, an acquisition time of 3.46 s, and a spectral width of 20 ppm followed by a repetition delay of 4 s. Experiments for the hydrophobic extracts were recorded using standard single-pulse excitation with the Bruker pulse program zg with 64 scans, an acquisition time of 8 s, and a spectral width of 16 ppm followed by a repetition delay of 2 s.

Spectra were processed using MestReNova (v. 14.2, Mestrelab research, ES). Spectra for all aqueous extracts were referenced to DSS at 0.0 ppm, while spectra of all organic extracts were referenced using the solvent signal at 7.26 ppm. All spectra were processed using 1.5-Hz exponential apodization followed by an automatic phase correction and ablative baseline correction.

#### Liquid Chromatography–Mass Spectrometry

For metabolite analysis by ultrahigh-performance liquid chromatography coupled to quadrupole time-of-flight mass spectroscopy, aqueous extracts from spiders acclimated at 19 or 29 °C were used (43 samples after CCRTemp treatment, 40 samples after CTmax treatment). The freeze-dried methanol/water extract was resuspended in 0.1% formic acid and run on an Acquity UPLC I-Class system (Waters Corporation, USA) coupled to a Q-TOF maXis Impact mass spectrometer (Bruker Daltonics GmbH, Germany) operated in positive (ESI+) or negative (ESI−) ionization mode. For details of run conditions, peak detection, and feature identification, we followed Sandfeld et al. (2022). The level of metabolite/feature identification was designated according to the guidelines of the Metabolomics Standard Initiative (Sumner et al. 2007) and can be found in supplementary table S2, Supplementary Material online.

#### Metabolite Data Analyses

Peak-intensity tables were obtained using the online tool MetaboAnalyst v. 4.0 (Xia and Wishart 2011), but subsequent analyses were done using MetaboAnalystR v. 3.2.0 (Pang et al. 2020) in R v. 4.05 (R Core Team 2021). Both LC-MS and NMR data were normalized to sum and Pareto scaled before we tested for population, acclimation temperature, and interaction effects using ANOVA with fdr < 0.05 from within MetaboAnalystR.

### Bacterial Microbiome

#### DNA Extraction and Sequencing

We screened the microbiome of spiders from acclimation temperatures 19 and 29 °C to evaluate whether there were temperature-induced differences among spiders in two very different temperatures, before proceeding with additional analyses. As we did not detect differences (see Results), we did not pursue further microbiome analyses. Three adult spiders from each nest box at 19 and 29 °C (120 individuals in total) were sampled, and whole spiders were used for DNA extraction (as described above) and amplicon sequencing of the V3 to V4 region of the 16S rRNA following the protocol in Busck et al. (2020). The reads were quality filtered using cutadapt v. 0.1.1 (Martin 2011), and ASVs were assigned using the DADA2 R package v. 1.18.0 (Callahan et al. 2016) and taxonomically classified according to the Silva SSU reference database nr. 132 (Quast et al. 2013). Finally, the relative abundances of the ASVs were calculated.

#### Microbiome Analysis

The microbiome revealed a total of 1,049 bacterial ASVs. These were filtered using a prevalence threshold, to retain only ASVs that are present in >25% of nests within a population, retaining 78 ASVs. This threshold was chosen somewhat arbitrarily to focus on the ASVs that are most likely to be ecologically relevant within populations based on prevalence. Following filtration, ANOVA (fdr < 0.05, *P* < 0.05) was used to test the effects of population, acclimation temperature, and their interaction on each ASV.

#### Visualizations of Modalities

All molecular variant data sets were analyzed and visualized by PCA using base R prcomp(). Metabolite data were furthermore analyzed and visualized by PLS-DA using the functions PLSR.Anal() and a modification of PlotPLS2DScore from the MetaboAnalystR package v. 3.2.0 (Pang et al. 2020). Plots for temperature tolerances and plots containing single genes, metabolites, and ASVs were plotted using Plot() and trendlines estimated by lm().

### Analyses of Interactions between the Environment and Specific Modalities Affecting Temperature Tolerance Phenotypes

We aimed to identify molecular variants that are associated with the different acclimation responses found among populations in the two different temperature tolerance measures CTmax and CCRT. To this end, we identified acclimation responses in modalities (transcriptome, methylome, metabolome, and microbiome) that responded in a similar pattern as the phenotypes CTmax and CCRT. For CTmax, the acclimation responses (slopes) were significantly different from 0 and in the same direction in Betta, Otavi, and Stampriet, while we identified no significant acclimation response in Karasburg. Modalities that showed similar acclimation responses were identified as those in which (i) the slopes estimated in the linear model (lm) were not significantly different from 0 and had an absolute value lower than 0.02 for Karasburg and (ii) were significantly different from 0, in the same direction and with absolute values higher than 0.02 for Betta, Otavi, and Stampriet. For CCRT, the acclimation responses were significant and in the same direction in Karasburg and Stampriet, while the acclimation responses were not different from 0 in Betta and Otavi. Modalities that showed similar acclimation responses were obtained by the following requirements: (i) the slopes estimated in the linear model should have an absolute value lower than 0.02 for Betta and Otavi and (ii) the slopes estimated in the linear model should all be significantly different from 0 and all have absolute values in same direction and higher than 0.02 for Karasburg and Stampriet. Only genes with a variance stabilizing transformation transcript count above 4.5 were considered in the gene expression analyses to exclude loci with low expression. To obtain an estimate of false positives, we ran the same filters described above (for CTmax and CCRTemp) on data sets with gene expression and metabolite intensity data permuted/resampled using sample() on each gene/metabolite. Gene ontology enrichment analyses were run for the genes with expression level that passed the filters for similarity to either CTmax or CCRTemp responses, respectively. The functional annotation was done using Eggnog orthology and the Eggnog mapper (emapper-2.1.9) using Diamond Search v. 0.9.21 (Huerta-Cepas et al. 2019; Buchfink et al. 2021; Cantalapiedra et al. 2021). Functional enrichment analyses were run using the Bioconductor R packages GoStats v. 2.60.0 and GSEAbase v. 1.56.0. (Falcon and Gentleman 2007; Morgan et al. 2022), and enriched terms with both count = 1 and size = 1 were removed.

## Supplementary Material

evae165_Supplementary_Data

## Data Availability

Transcriptome data can be accessed under bioproject PRJNA510316. Methylation data can be found under bioproject PRJNA808424. Microbiome data can be found under bioproject PRJNA962689. The code is available in https://github.com/Anneaa2/Stegodyphus_common_garden.
