# Supplementary material for: Molecular Mechanisms of Temperature Tolerance Plasticity in an Arthropod
Source: Genome Biol Evol. 2024 Jul 26;16(8):evae165. doi: 10.1093/gbe/evae165 (PMC11979766; doi:10.1093/gbe/evae165)
Supplement: evae165_Supplementary_Data [file evae165_supplementary_data.docx]

Supplementary materials for:

Anne Aagaard, Jesper Bechsgaard, Jesper Givskov Sørensen, Tobias Sandfeld, Virginia Settepani, Tharina L. Bird, Marie Braad Lund, Kirsten Gade Malmos, Kasper Falck-Rasmussen, Iulia Darolti, Kirstine Lykke Nielsen, Mogens Johannsen, Thomas Vosegaard, Tom Tregenza, Koen J.F. Verhoeven, Judith E. Mank, Andreas Schramm and Trine Bilde

**Molecular mechanisms of temperature tolerance plasticity in a social spider.**

## Contents

[Supplementary materials for: 1](#_Toc167178828)

[Contents 1](#_Toc167178829)

[Figure S1: Realized ramping temperatures for CTmax and CCRTemp experiments 3](#_Toc167178830)

[Figure S2: Growth and survival of spiders in acclimation treatments 5](#_Toc167178831)

[Figure S3: Plots of additional explanatory variables for CTmax treated spiders 6](#_Toc167178832)

[Figure S4: Plots of additional explanatory variables for CCRTemp treated spiders 7](#_Toc167178833)

[Figure S5: Details on body mass differences in acclimation treatments and populations 8](#_Toc167178834)

[Figure S6: Number of genes with responses to population and temperature in gene expression and DNA methylation 9](#_Toc167178835)

[Figure S7: PCA of genes with effect of population and temperature in expression level 10](#_Toc167178836)

[Figure S8: PCA of genes with interaction effect in expression level 11](#_Toc167178837)

[Figure S9: PCA of genes with population effect in weighted methylation level 12](#_Toc167178838)

[Figure S10: Histogram of correlation coefficients between methylation and gene expression 13](#_Toc167178839)

[Figure S11: Histogram of correlation coeficients between methylation and stability of gene expression 14](#_Toc167178840)

[Figure S12: Scatter- and violin plot of gene-wise stability of gene expression as a function of methylation level 15](#_Toc167178841)

[Figure S13: Number of metabolites with responses to population and temperature in LCMS and NMR analyses 16](#_Toc167178842)

[Figure S14: PCA of metabolite LCMS data from the CTmax treatment 17](#_Toc167178843)

[Figure S15: PLS-DA analysis of metabolite data from the CTmax treatment 18](#_Toc167178844)

[Figure S16: Metabolite intensities for named LC-MS metabolites for CTmax tested spiders. 19](#_Toc167178845)

[Figure S17: PCA of metabolite LCMS data from the CCRTemp treatment 24](#_Toc167178846)

[Figure S18: PLS-DA analysis of metabolite data from the CCRTemp treatment 25](#_Toc167178847)

[Figure S19: Metabolite intensities for named LC-MS metabolites for CCRTemp tested spiders. 26](#_Toc167178848)

[Figure S20: PCA of metabolite NMR-Aq data from the CTmax treatment 31](#_Toc167178849)

[Figure S21: PCA of metabolite NMR-Aq data from the CCRTemp treatment 32](#_Toc167178850)

[Figure S22: Number of ASVs with responses to population and temperature in microbiome 33](#_Toc167178851)

[Figure S23: Gene expression with similar population specific acclimation responses as the heat tolerance phenotype 34](#_Toc167178852)

[Figure S24: Gene expression with similar population specific acclimation responses as the cold tolerance phenotype 48](#_Toc167178853)

[Figure S25: Hydrophilic metabolites with similar population specific acclimation responses as the heat tolerance phenotype 52](#_Toc167178854)

[Figure S26: Hydrophobic metabolites with similar population specific acclimation responses as the heat tolerance phenotype 53](#_Toc167178855)

[Figure S27: Hydrophilic metabolites with similar population specific acclimation responses as the cold tolerance phenotype 54](#_Toc167178856)

[Figure S28: Phylogenies with scenarios for gain/loss of plasticity in heat and cold tolerance 55](#_Toc167178857)

[Figure S29: PCA of microbiomic data 56](#_Toc167178858)

[Figure S30: Plots of microbiomic ASVs with population effect 57](#_Toc167178859)

[Figure S31: Coverage plot for methylation data 58](#_Toc167178860)

[Figure S32: PCA of metabolite NMR-Org data from the CTMax and CCRTemp treatment 59](#_Toc167178861)

[Figure S33: Methylation in plastically expressed genes in response to temperature acclimation 60](#_Toc167178862)

[Table S1: Number of social spider *S. dumicola* nest replicates per population/acclimation group 61](#_Toc167178863)

[Table S2: Table of LCMS metabolites and their effects 62](#_Toc167178864)

[Table S3: Model summary output for growth rate model 68](#_Toc167178865)

[Table S4: ANOVA output for model on growth rate 69](#_Toc167178866)

[Table S5: ANOVA output for model on survival 70](#_Toc167178867)

[Table S6: Model summary for survival model 71](#_Toc167178868)

[Table S7: Test output on whether CTmax population trends differ from zero 72](#_Toc167178869)

[Table S8: Test output on whether CCRTemp population trends differ from zero 73](#_Toc167178870)

[Table S9: Best model summary on CTmax data 74](#_Toc167178871)

[Table S10: ANOVA output from CTmax model 75](#_Toc167178872)

[Table S11: Test output on whether CTmax population trends differ 76](#_Toc167178873)

[Table S12: Model summary output for CCRTemp model 77](#_Toc167178874)

[Table S13: ANOVA output from CCRTemp model 78](#_Toc167178875)

[Table S14: Test output on whether CCRTemp population trends differ 79](#_Toc167178876)

[Table S15: Gene ontology enrichment analysis results 80](#_Toc167178877)

[Supplementary comment 1: Potential confounding factors: 82](#_Toc167178878)

[References for supplement: 83](#_Toc167178879)

## Figure S1: Realized ramping temperatures for CTmax and CCRTemp experiments

The relationship between time and temperature for determining a) CTmax and b) CCRTemp. Coloured circles are measurements of temperature at a given time during ramping, while black circles indicate the mean with standard deviation. A linear model has been fitted on the average: lm = 0.098x + 42.99 for CTmax, and lm = 0.5x + 0.254 for CCRTemp. The number of measurements per timepoint are in table below graphs.


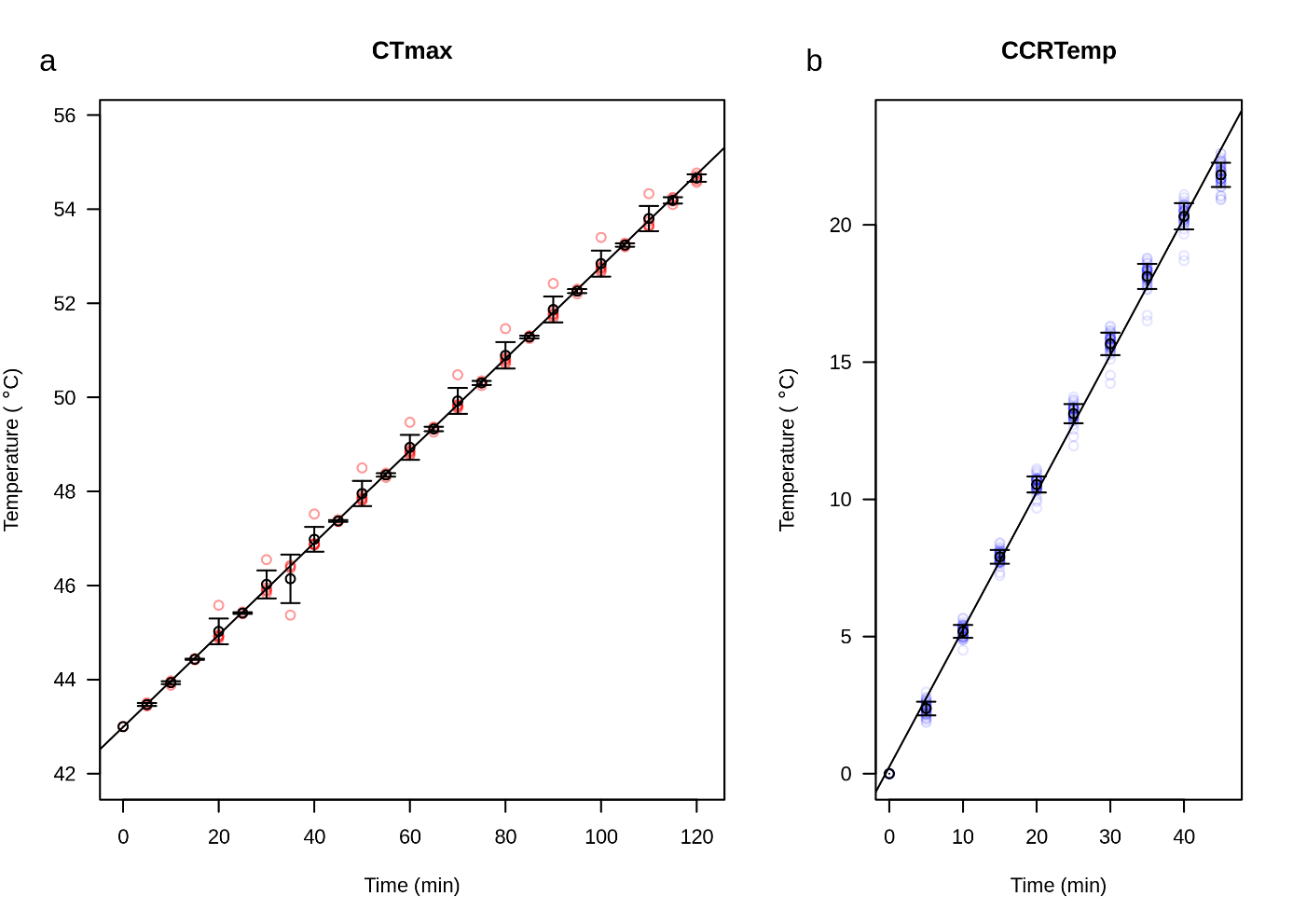


| time | N measurements CTmax | N measurements CCRTemp |
| --- | --- | --- |
| 0 | 6 | 38 |
| 5 | 4 | 38 |
| 10 | 6 | 38 |
| 15 | 4 | 38 |
| 20 | 6 | 38 |
| 25 | 4 | 38 |
| 30 | 5 | 38 |
| 35 | 4 | 38 |
| 40 | 6 | 37 |
| 45 | 4 | 36 |
| 50 | 6 |  |
| 55 | 4 |  |
| 60 | 6 |  |
| 65 | 4 |  |
| 70 | 6 |  |
| 75 | 4 |  |
| 80 | 6 |  |
| 85 | 4 |  |
| 90 | 6 |  |
| 95 | 4 |  |
| 100 | 6 |  |
| 105 | 4 |  |
| 110 | 6 |  |
| 115 | 4 |  |
| 120 | 5 |  |

## Figure S2: Growth and survival of spiders in acclimation treatments

Growth rate and survival data of *S. dumicola* spiders as a function of acclimation temperature. Linear models and trend lines have been added to growth rate (a) while binomial models and smoothed lines have been applied to survival data (b).


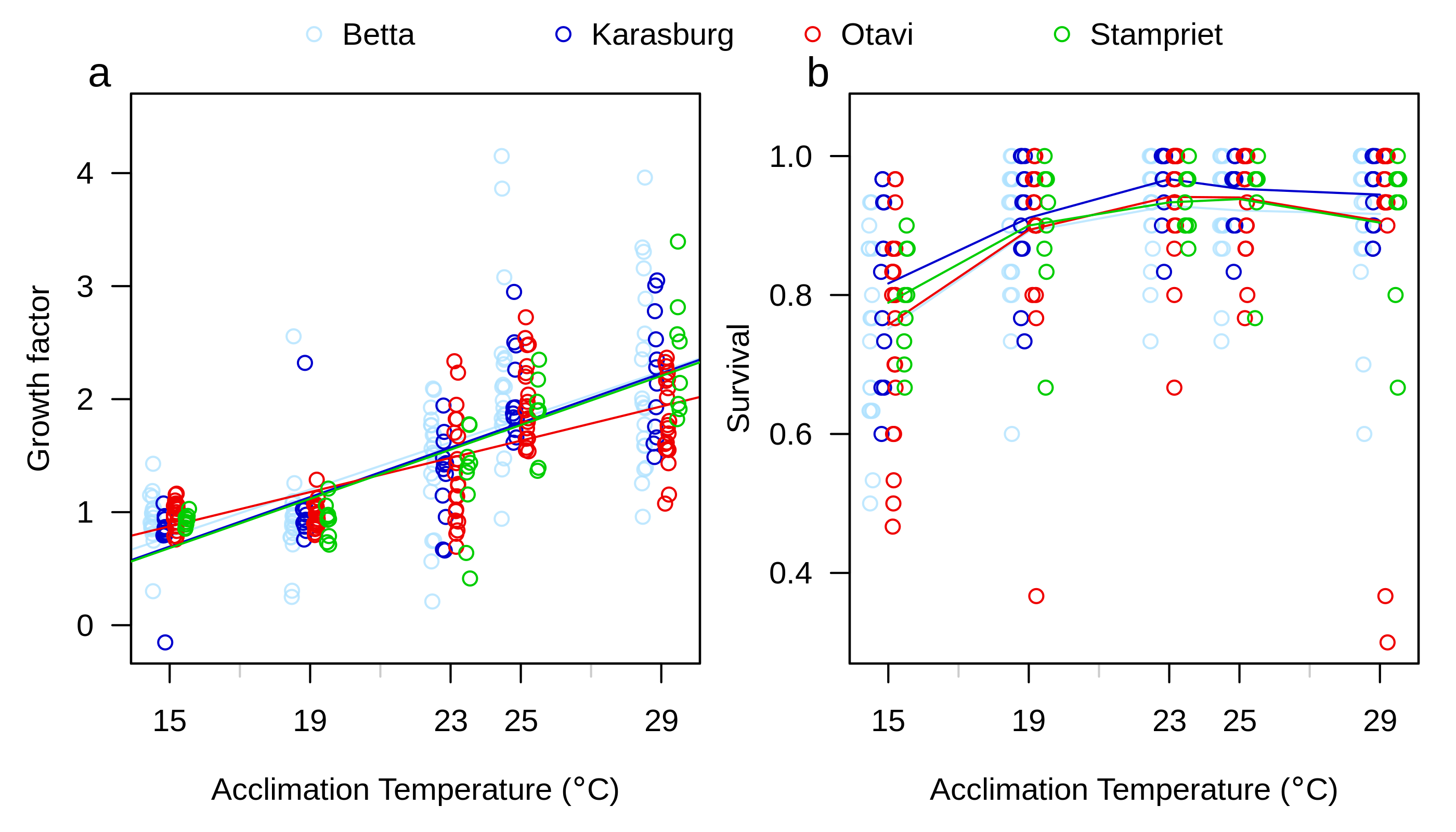


|  | **HSD.tests on models** | | | | |
| --- | --- | --- | --- | --- | --- |
|  |  | **Growth rate (HSD.test)** | | **Survival (emmeans)** | |
| Acclimation temperature effect | **Temperature** | **Estimate** | **Group** | **Estimate (logit)** | **Group** |
|  | 15 | 0.9221065 | c | 1.48 | a |
|  | 19 | 0.9705685 | c | 1.92 | b |
|  | 23 | 1.3633561 | b | 2.35 | c |
|  | 25 | 2.0595457 | a | 2.57 | d |
|  | 29 | 2.0895684 | a | 3.01 | e |
| Population effect | **Population** | **Estimate** | **Group** | **Estimate (logit)** | **Group** |
|  | Betta | 1.534714 | a | 2.11 | a |
|  | Karasburg | 1.484138 | a | 2.53 | b |
|  | Otavi | 1.411240 | a | 2.17 | a |
|  | Stampriet | 1.434181 | a | 2.23 | ab |

## Figure S3: Plots of additional explanatory variables for CTmax treated spiders

Plots of CTmax as a function of a) body mass (mg), (b) days since feeding, and (c) preacclimation duration (days at 21°C). A linear trend line has been added to the plots for easier interpretation.


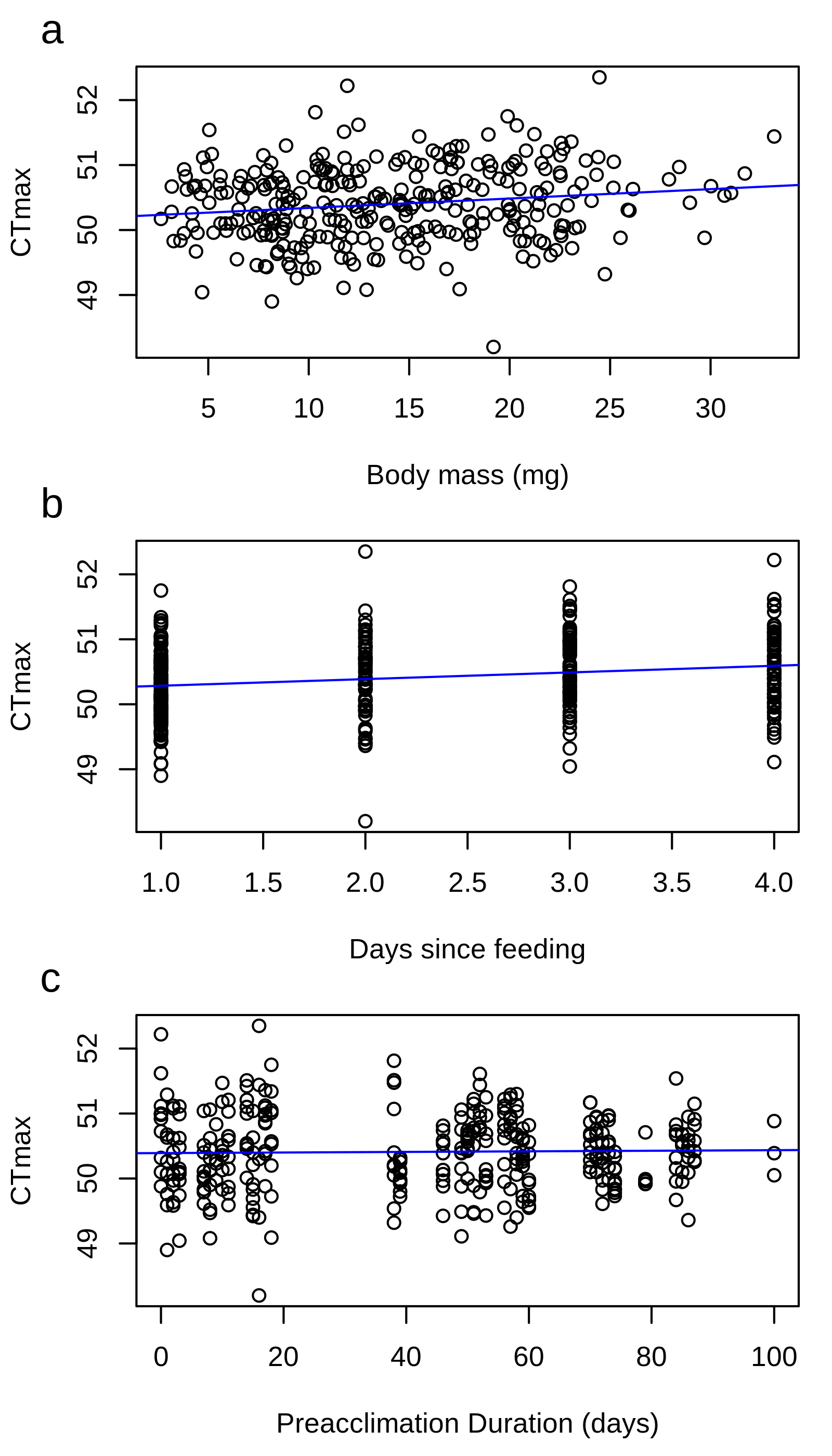


## Figure S4: Plots of additional explanatory variables for CCRTemp treated spiders

Plots of CCRTemp as a function of a) body mass (mg), (b) time since feeding, and (c) preacclimation duration (days at 21 °C). A linear trend line has been added to the plots for easier interpretation.


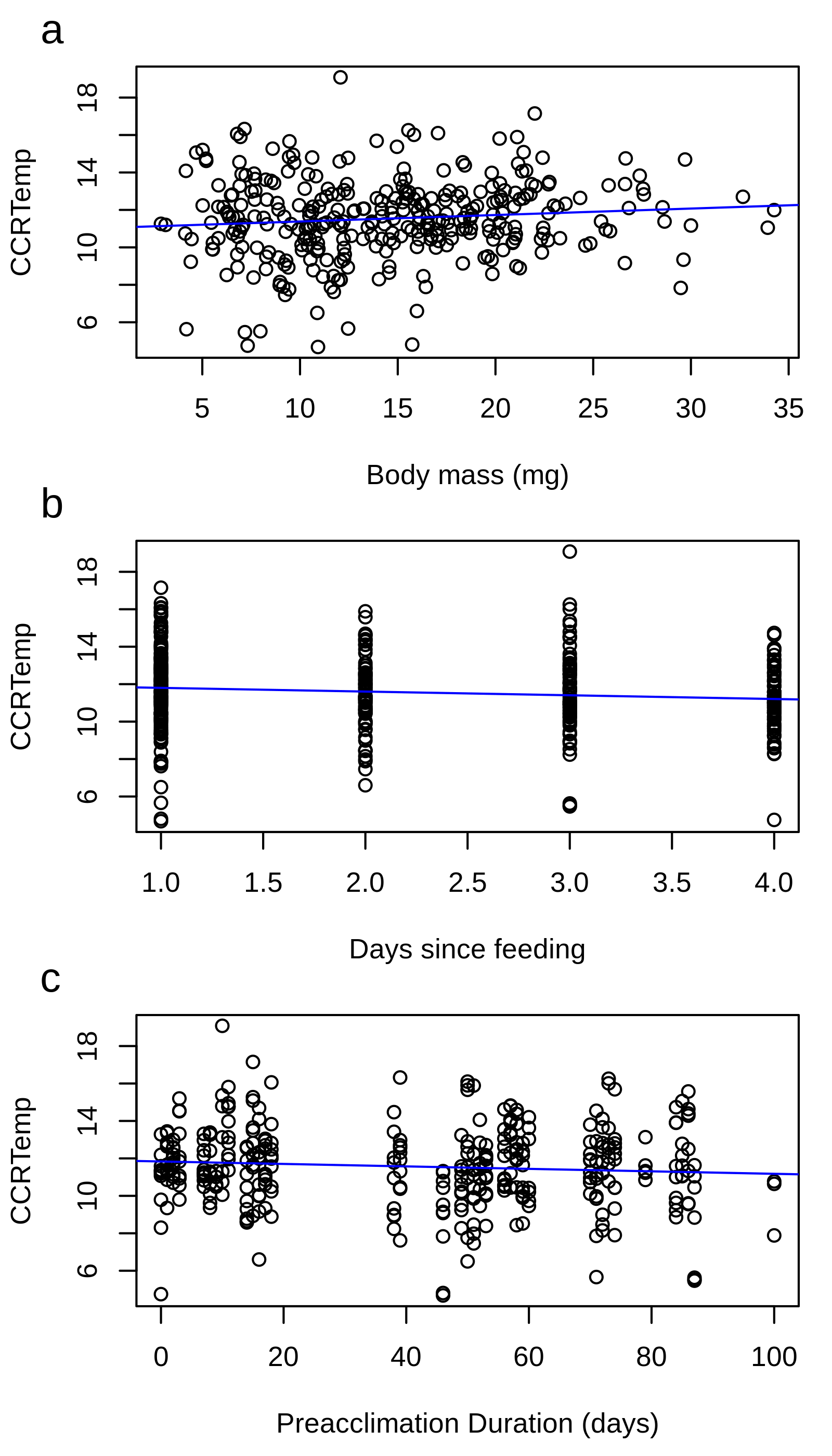


## Figure S5: Details on body mass differences in acclimation treatments and populations

Boxplots showing a, c) spider body mass (mg) and population, or b,d) acclimation temperature for spiders used in a, b) CTmax and c, d) CCRTemp assays. For both CTmax and CCRTemp, Betta and Karasburg spider body mass are not statistically different, while both Otavi and Stampriet spider body mass are significantly different from Betta and Karasburg (significance groups annotated on boxplots, ). Body mass increases with acclimation temperature (b, d).


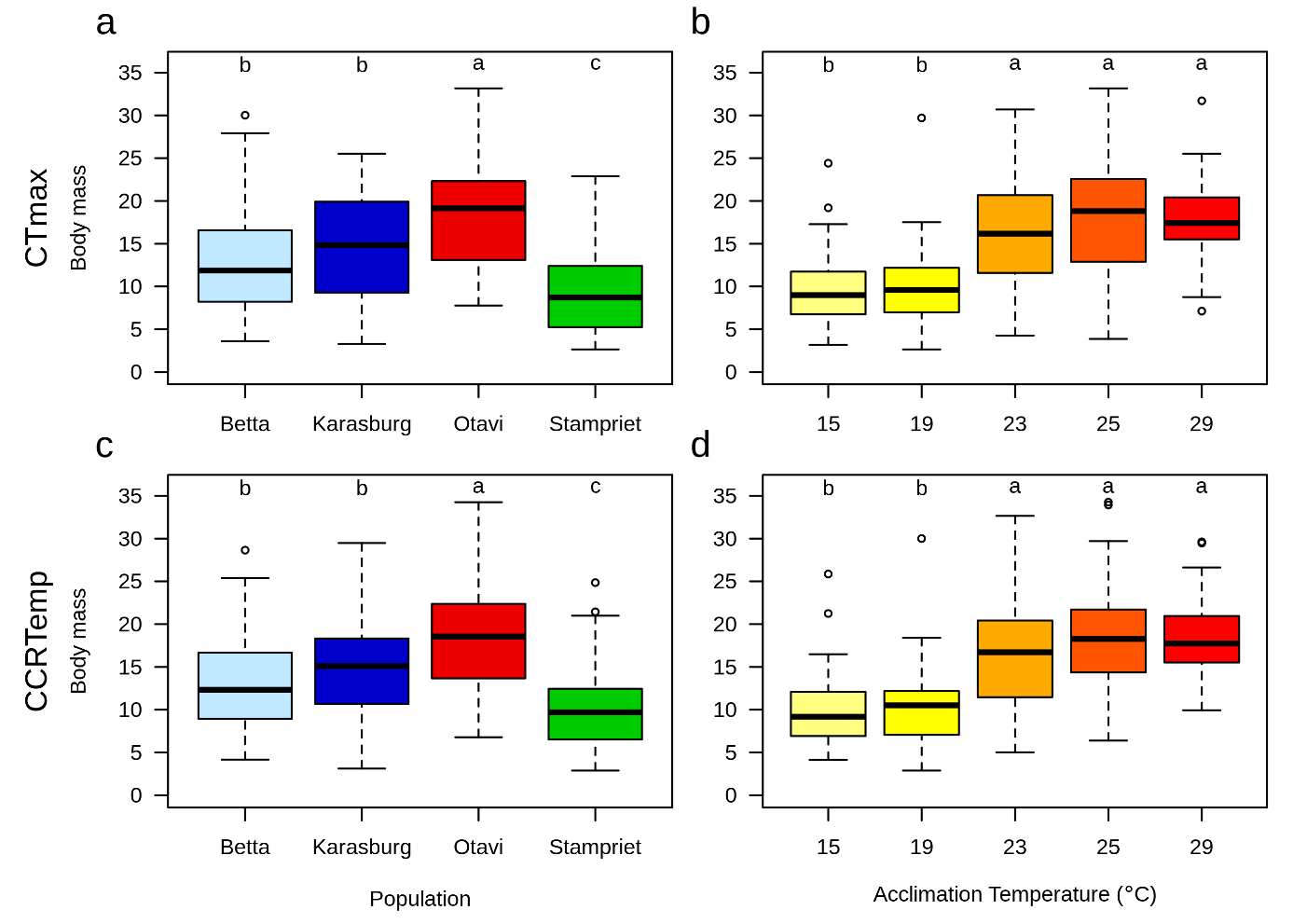


## Figure S6: Number of genes with responses to population and temperature in gene expression and DNA methylation

Barplots with the number of genes showing population and temperature responses in gene-wise a) gene expression and b) DNA methylation data. The y axis represents genes showing population responses (Population), acclimation temperature responses (Temperature), both population and temperature responses (Pop+Temp) and an interaction between population and temperature (Interaction). The hashed lines indicate the number of genes with a population response in both DNA methylation and gene expression. The arrow indicates the number of genes we expect to show a population response in both expression (DEG) and methylation (DMG) at random (N DMGs_pop_ / N all genes * N DEGs_pop_).


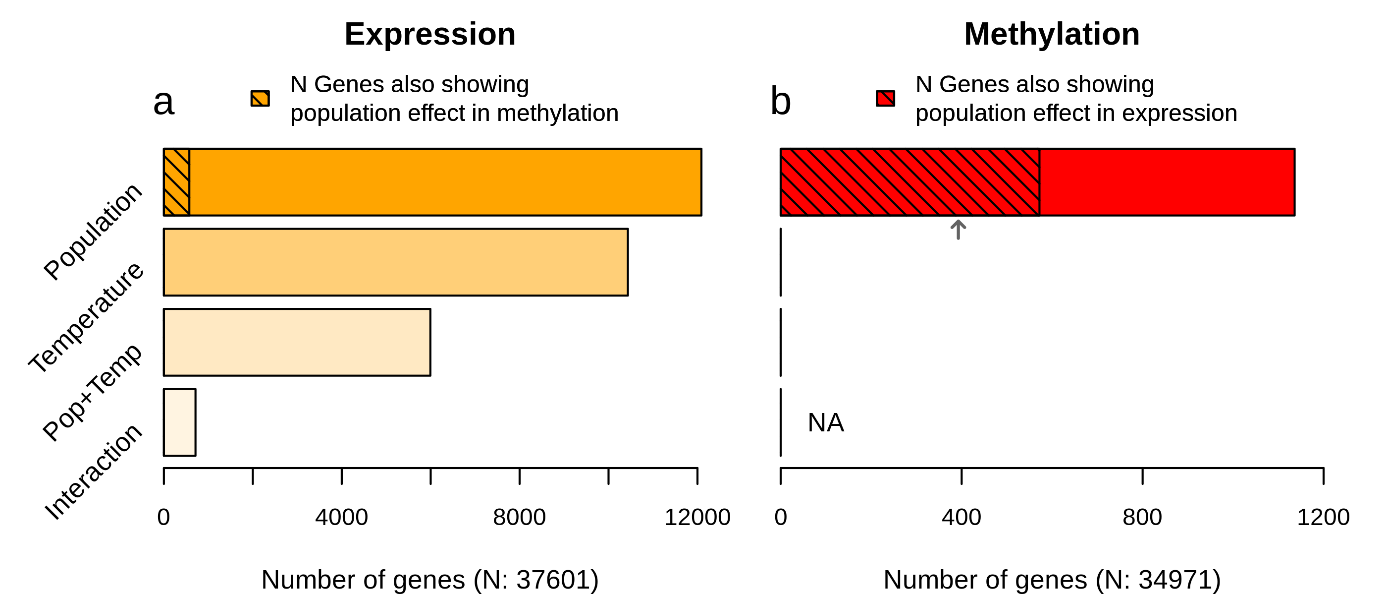


## Figure S7: PCA of genes with effect of population and temperature in expression level

Principal component analyses of differentially expressed genes between populations (a, c) and temperature acclimation (b, d). Here, three principal components are plotted. There is a tendency for Otavi (red) and Betta (light blue) to separate from Karasburg (blue) and Stampriet (green).


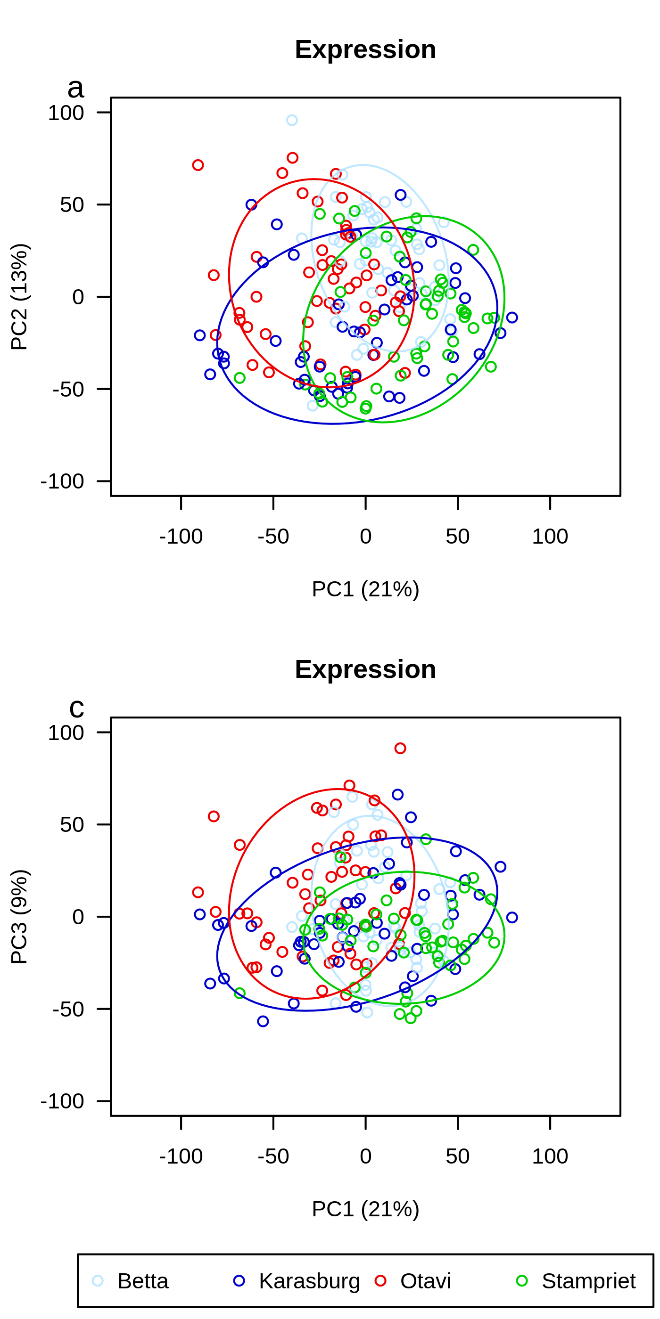

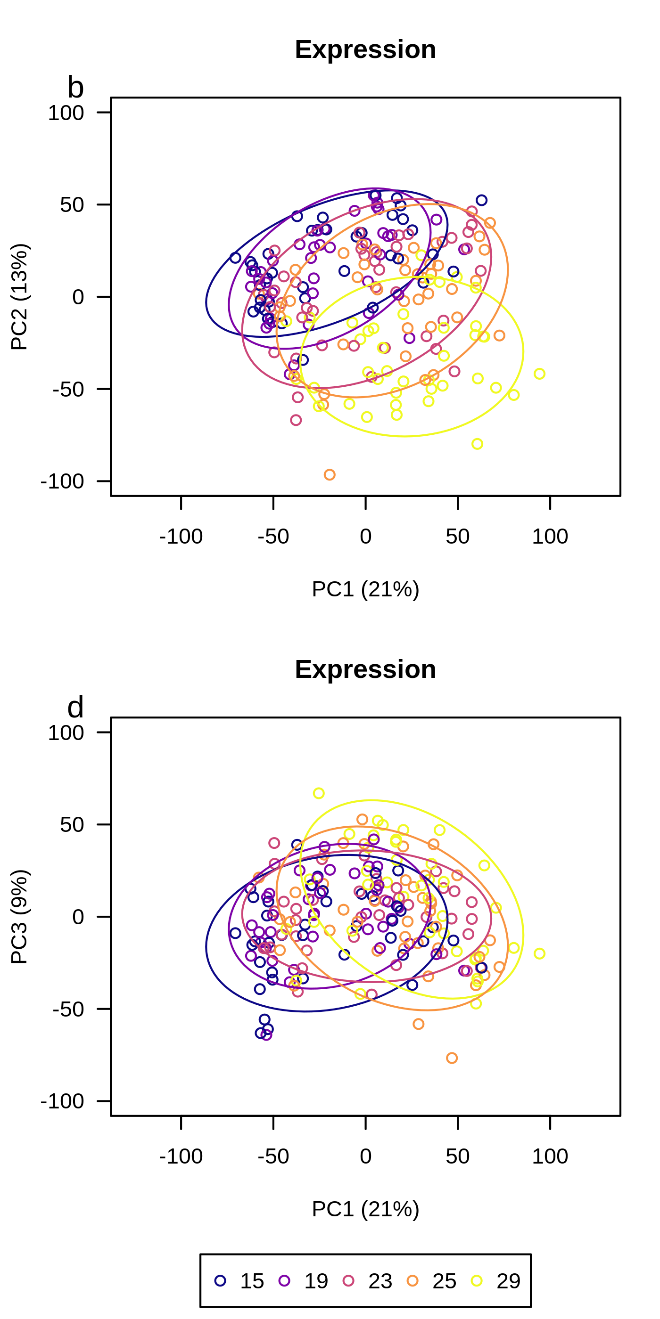


## Figure S8: PCA of genes with interaction effect in expression level

Principal component analyses of differentially expressed genes with interaction. Here, three principal components are plotted, along with a screeplot and a biplot. There is a tendency for Otavi (red) and Betta (light blue) to separate from Karasburg (blue) and Stampriet (green) on PC2.


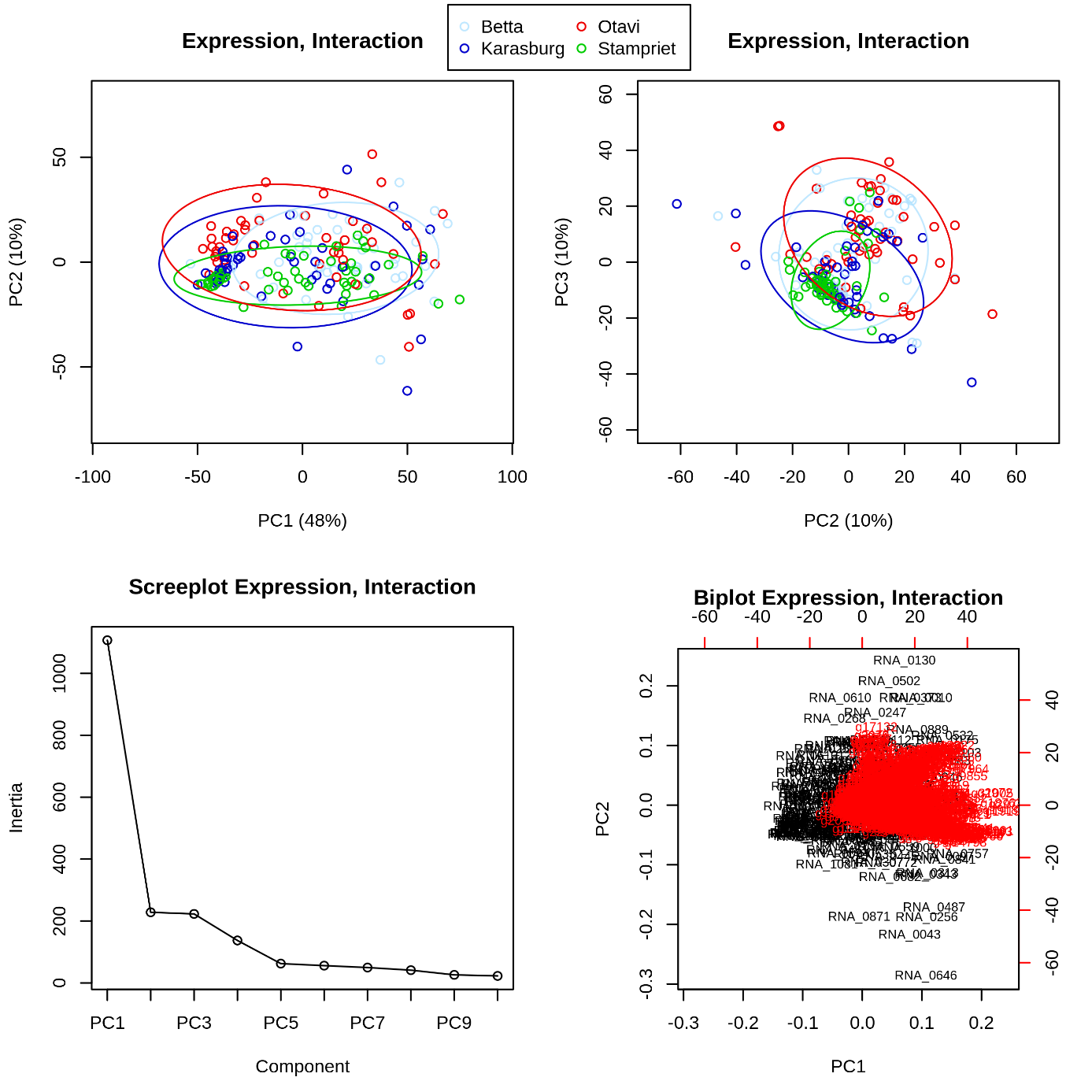


## Figure S9: PCA of genes with population effect in weighted methylation level

Principal component analyses of DNA Methylation and population of origin, including Scree- and biplot. Here, three principal components are plotted, along with a screeplot and a biplot. Otavi (red) and Betta (light blue) separate from Karasburg (blue) and Stampriet (green) on PC1.


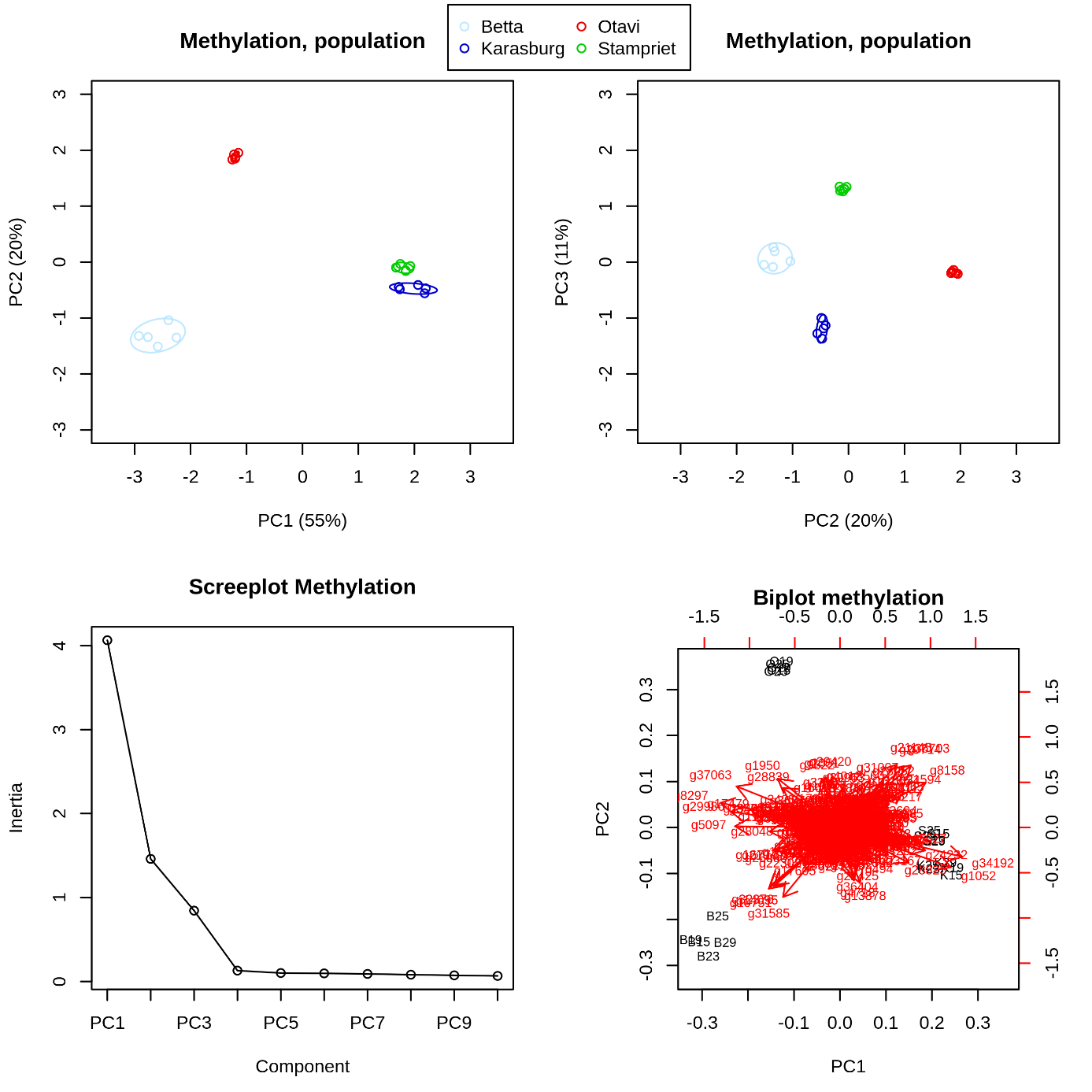


## Figure S10: Histogram of correlation coefficients between methylation and gene expression

Histogram of correlation coefficients between gene-wise weighted methylation level and vst transformed normalized gene expression level. All correlated genes showed a population response in both DNA methylation and gene expression. If the distribution is left skewed, then differences in methylation level between populations could be associated with population differences in gene expression. However, this seem to not be the case.


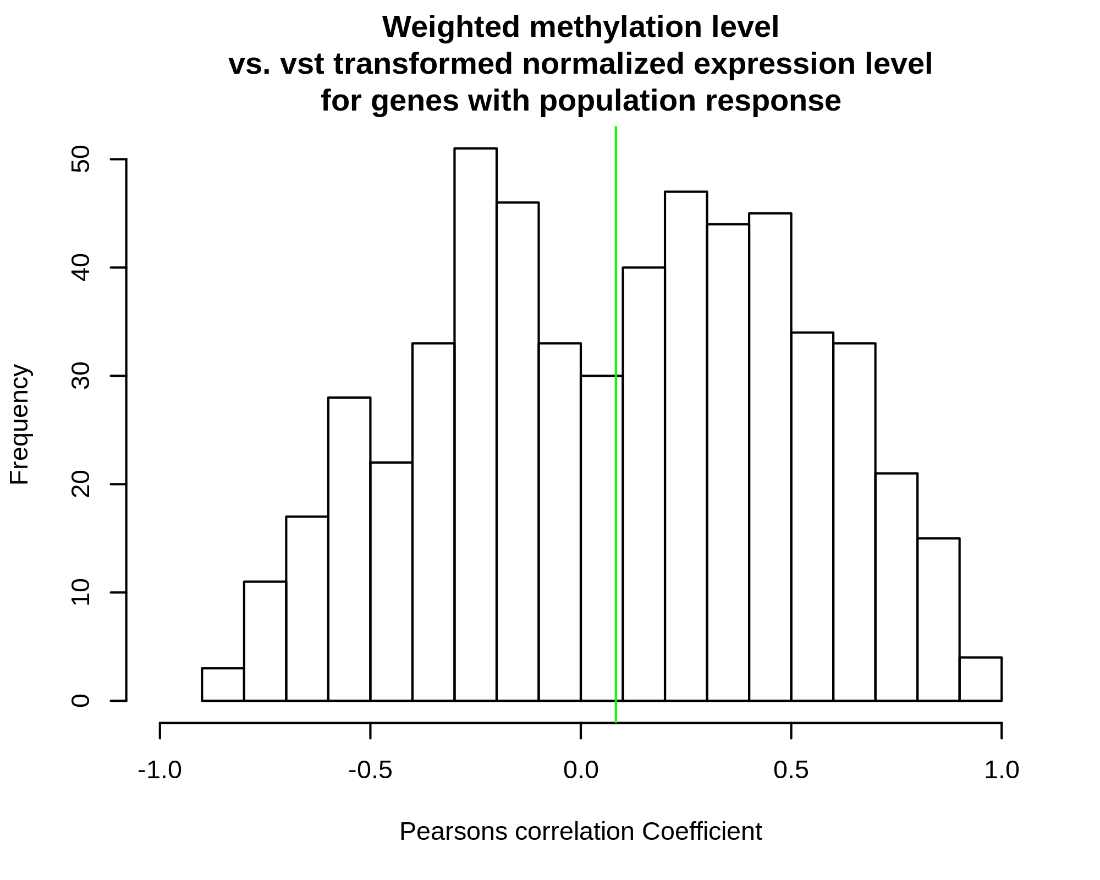


## Figure S11: Histogram of correlation coeficients between methylation and stability of gene expression

Histogram of correlation coefficients between weighted methylation level and Standard deviation of vst transformed normalized expression level. All correlated genes showed a population response in both DNA methylation and gene expression. If population specific higher methylation yield a more stable expression level, we would expect a right skew on the distribution. A very subtle right skew can be seen, indicating that such relation is not applicable across all genes.


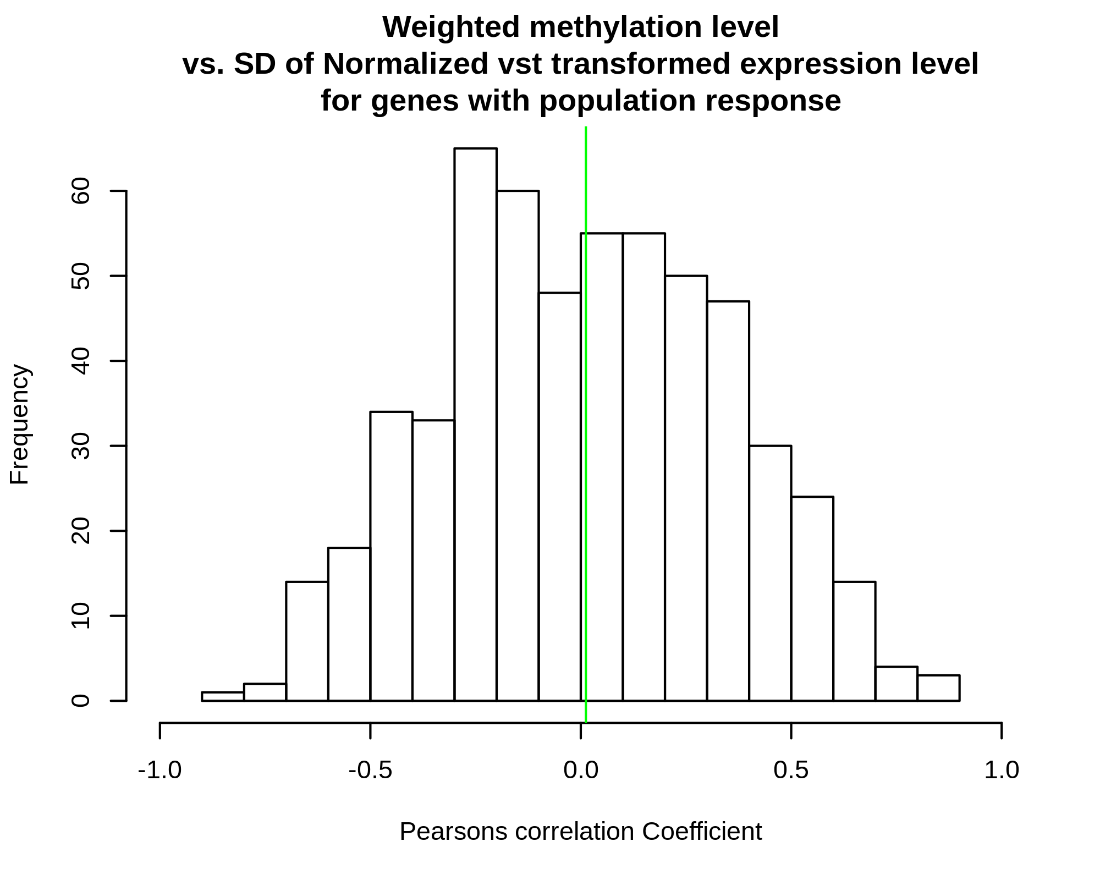


## Figure S12: Scatter- and violin plot of gene-wise stability of gene expression as a function of methylation level

Standard deviation in expression (vst transformed for visualisation) in individual genes as a function of weighted methylation level plotted as a) scatterplot and b) violin plot with categorized x axis. Only genes showing effects of population in methylation level was included. Each gene has 20 points on the dot plot (a), one from each acclimation/treatment groups, and colours indicate population. Violin plot (b) shows the stability of gene expression grouped by methylation level. Group limits were based on Liu et al., (2019). Low methylation level has a significantly larger standard deviation than medium and high methylation level (tested on normalised counts). The same result was found using all genes (not shown).


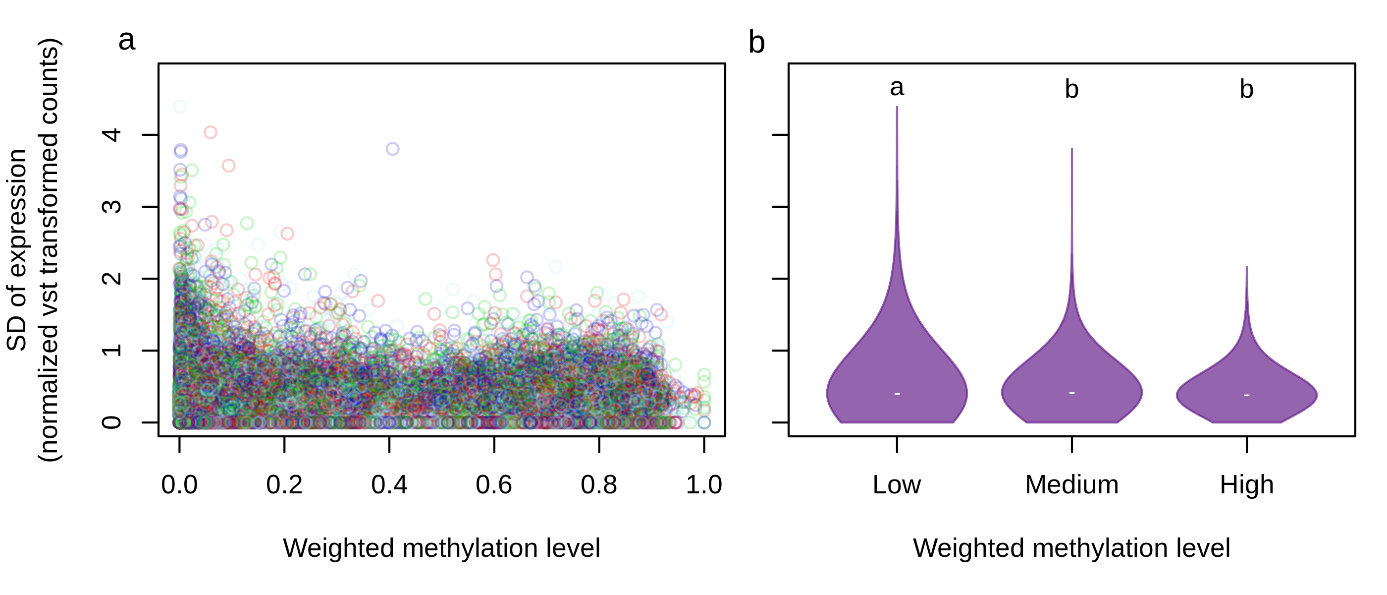


## Figure S13: Number of metabolites with responses to population and temperature in LCMS and NMR analyses

Barplots with metabolome data for spiders having undergone CTmax and CCRTemp assays after common garden acclimation. a,b) LC-MS identified metabolites of spiders after a) CTmax assays and b) CCRTemp assays. c,d) NMR peaks of spiders extracted by aquatic solution (AQ, solid bars) or organic solution (ORG, hashed bars), after c) CTmax assay and d) CCRTemp assay. The total number of metabolites or peaks identified can be seen on the x axis label (a,b) or the legend (c,d).

**
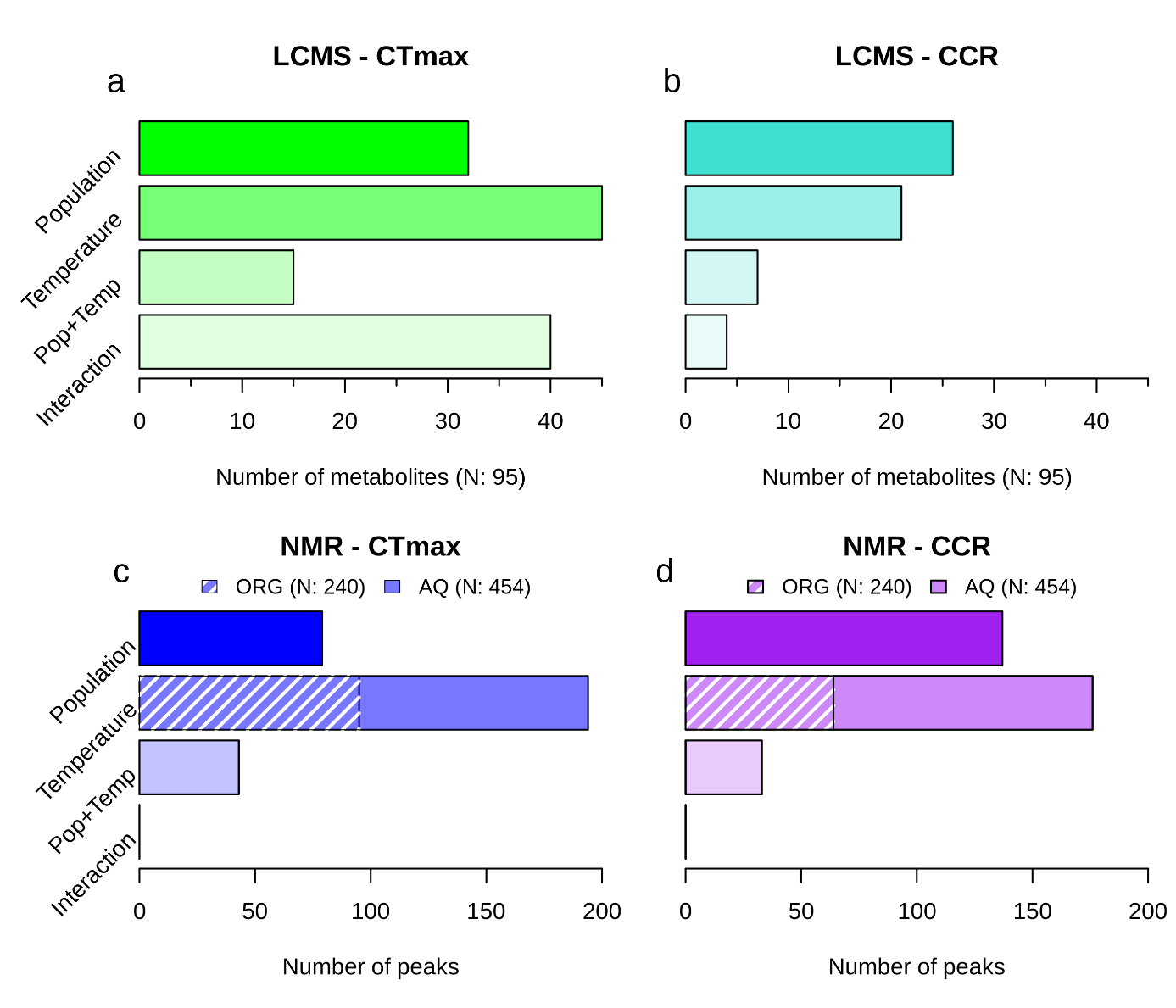
**

## Figure S14: PCA of metabolite LCMS data from the CTmax treatment

Principal component analysis of metabolites from the LC-MS analysis for spiders having undergone CTmax treatment, colored according to population and temperature acclimation. Only metabolites that showed population effects (a, c) or temperature effect (b,d) are plotted. Here the first three principal components are plottet. There is a tendency for Otavi (red) and Betta (light blue) to separate from Karasburg (blue) and Stampriet (green).


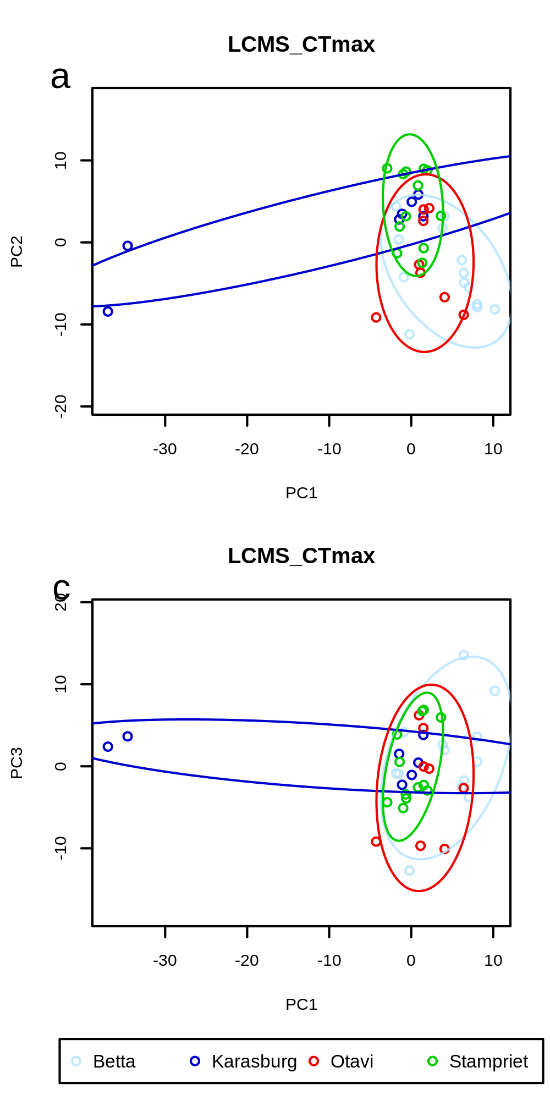

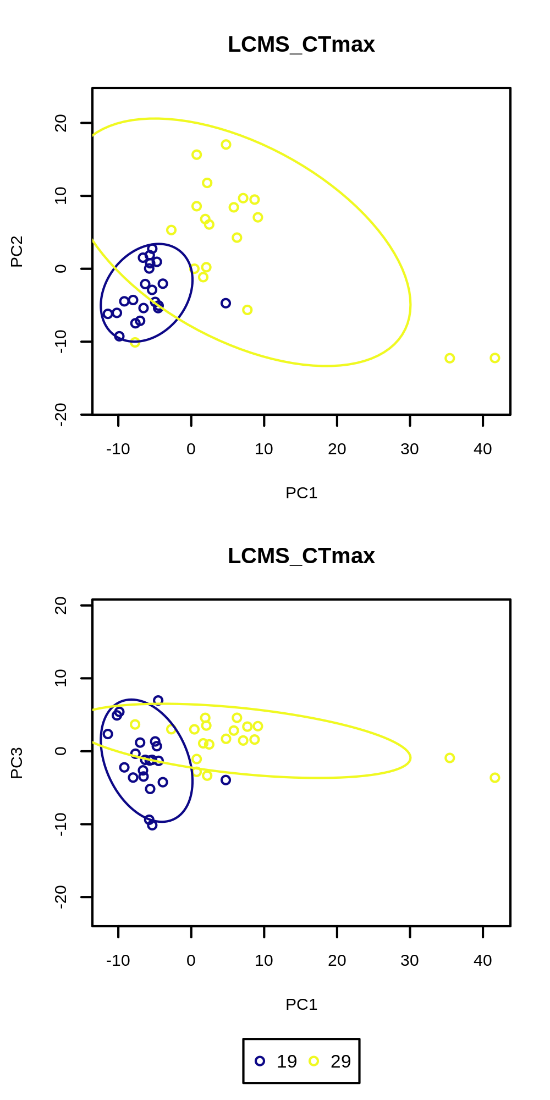


b

d

## Figure S15: PLS-DA analysis of metabolite data from the CTmax treatment

PLS-DA analysis of metabolites from the NMR analysis of spiders having been through CTmax testing. Points are metabolite intensities that show effect of either population (a, d) or temperature (b, c, e). Rows indicate metabolite extraction and analysis type: a, b) NMR aquatic extraction, c) NMR organic extraction and d, e) LC-MS.


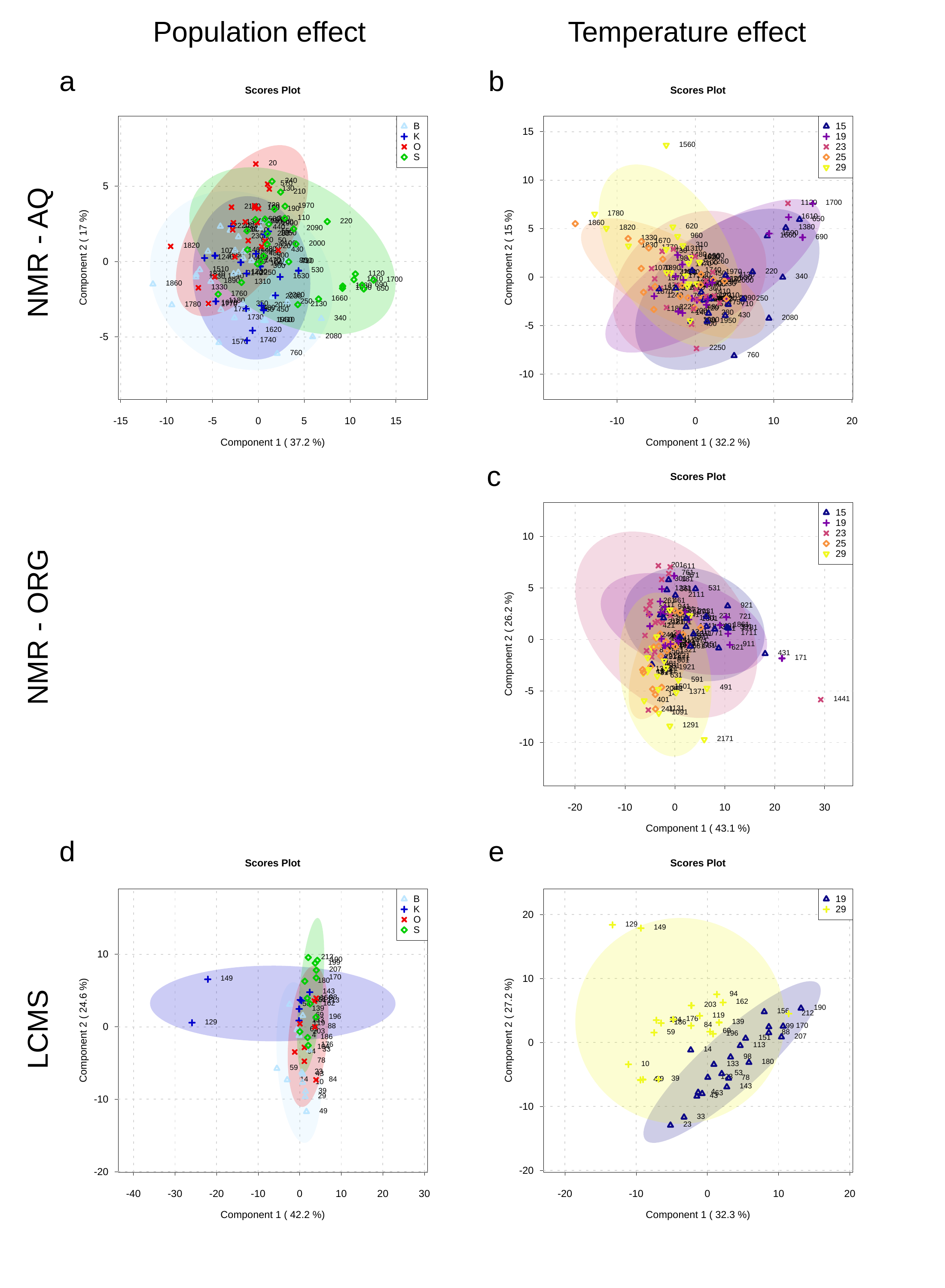


## Figure S16: Metabolite intensities for named LC-MS metabolites for CTmax tested spiders.

Metabolite intensities of all named LC-MS metabolites in CTmax performance tested spiders, giving a visualization of direction of change with temperature and difference between populations. Trendlines have been added for ease of interpretation.


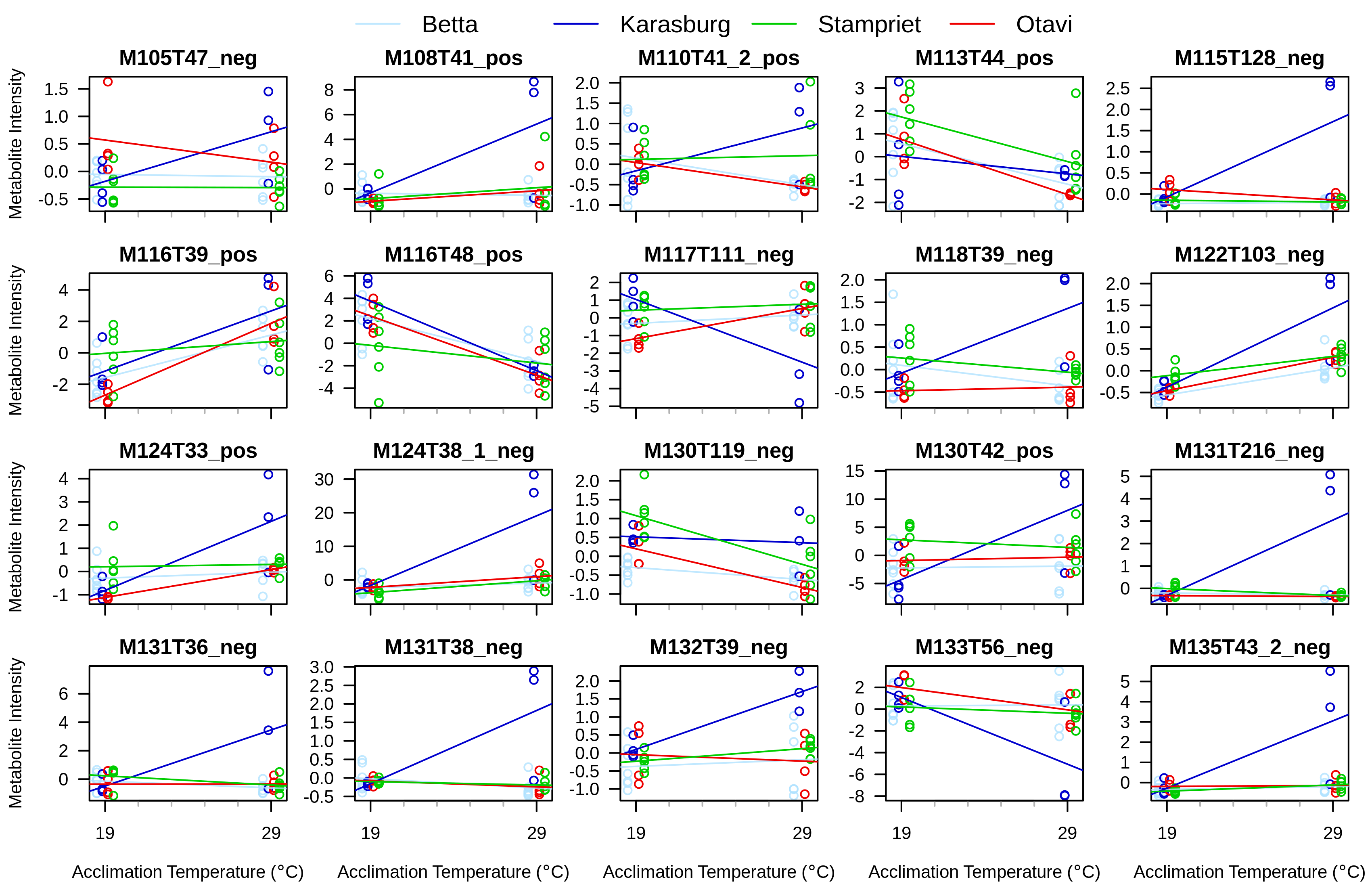


Figure S16 – continued: Metabolite intensities for named LC-MS metabolites for CTmax tested spiders.


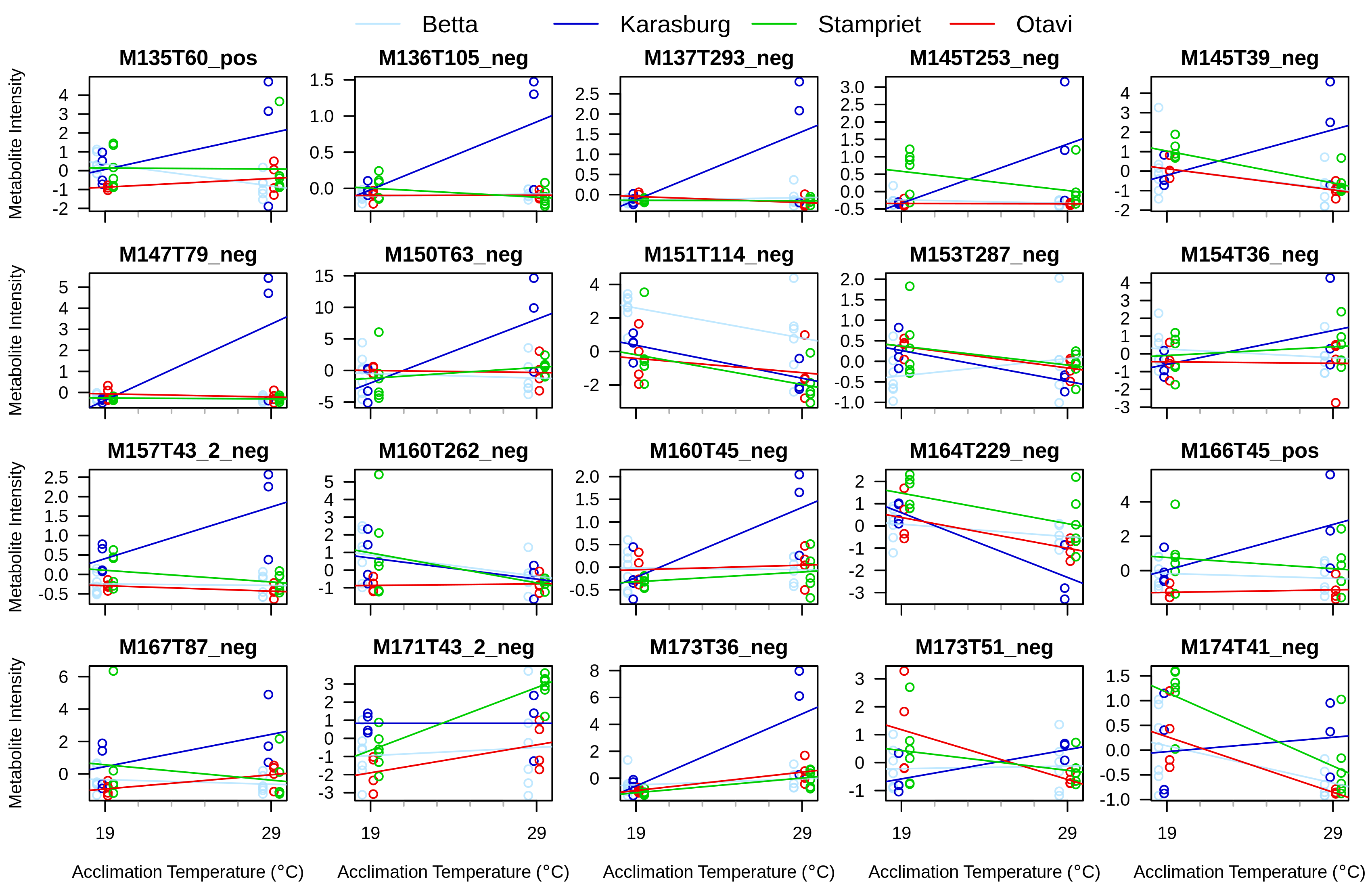


Figure S16 – continued: Metabolite intensities for named LC-MS metabolites for CTmax tested spiders.


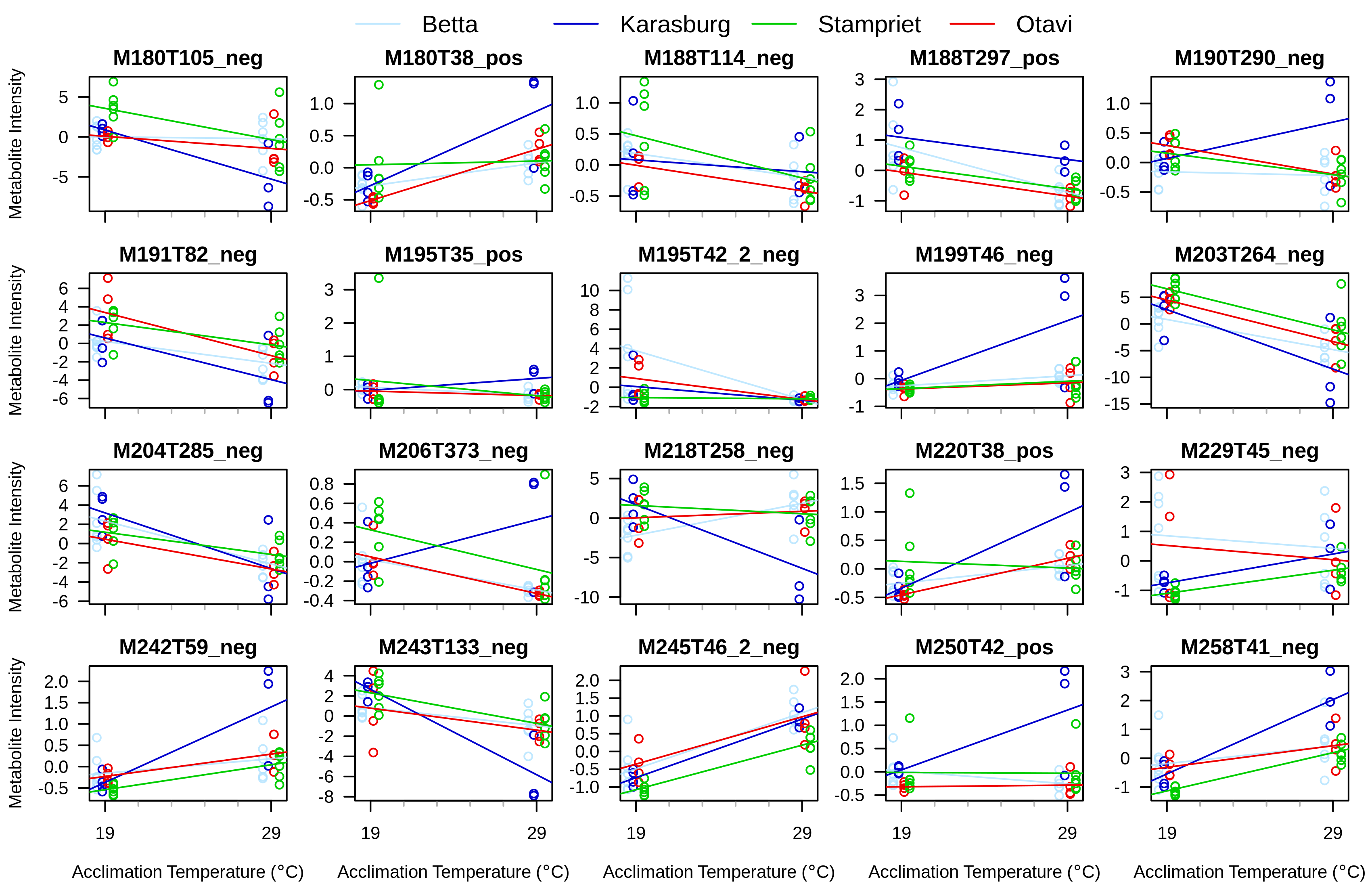


Figure S16 – continued: Metabolite intensities for named LC-MS metabolites for CTmax tested spiders.


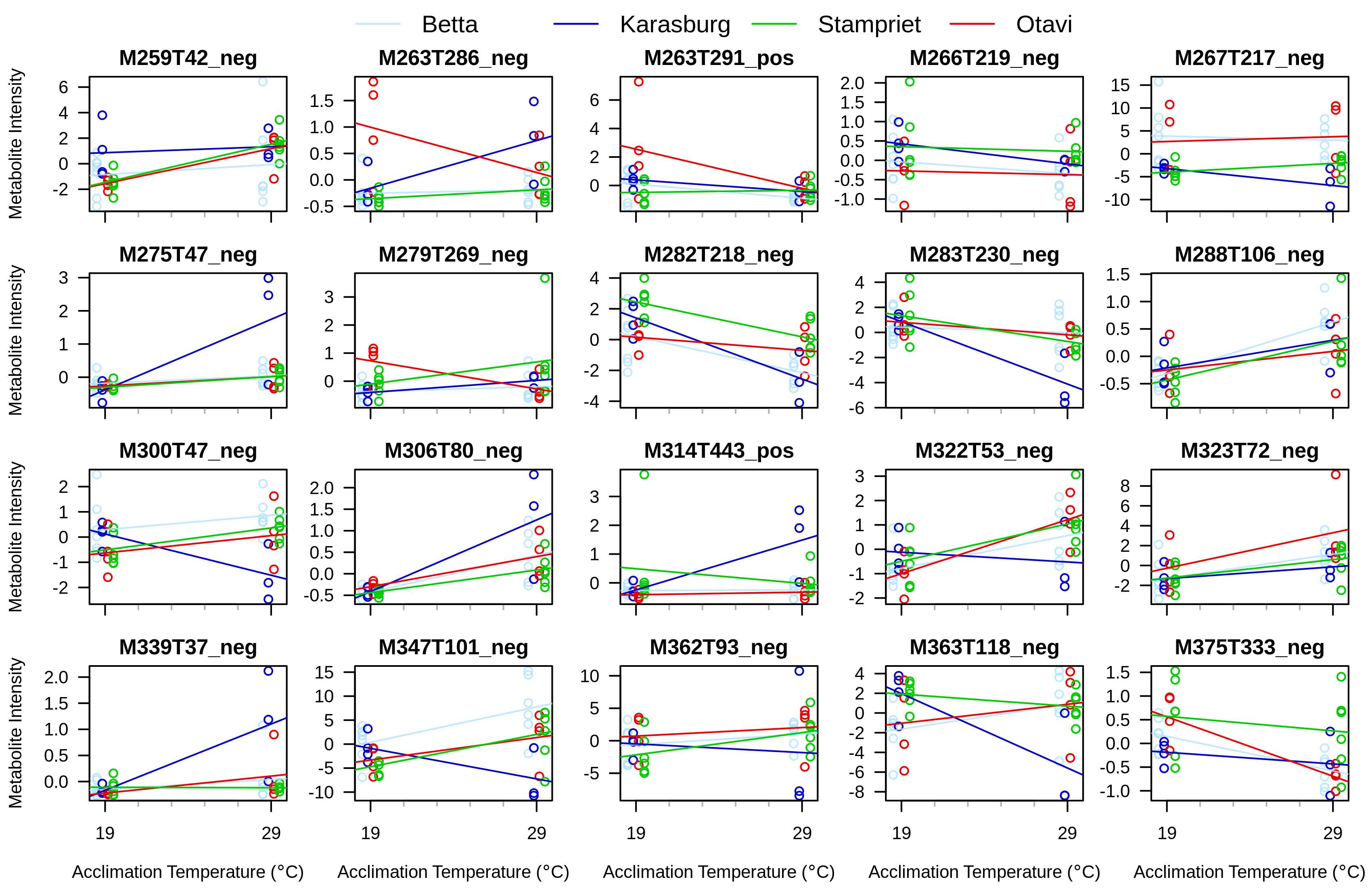


Figure S16 – continued: Metabolite intensities for named LC-MS metabolites for CTmax tested spiders.


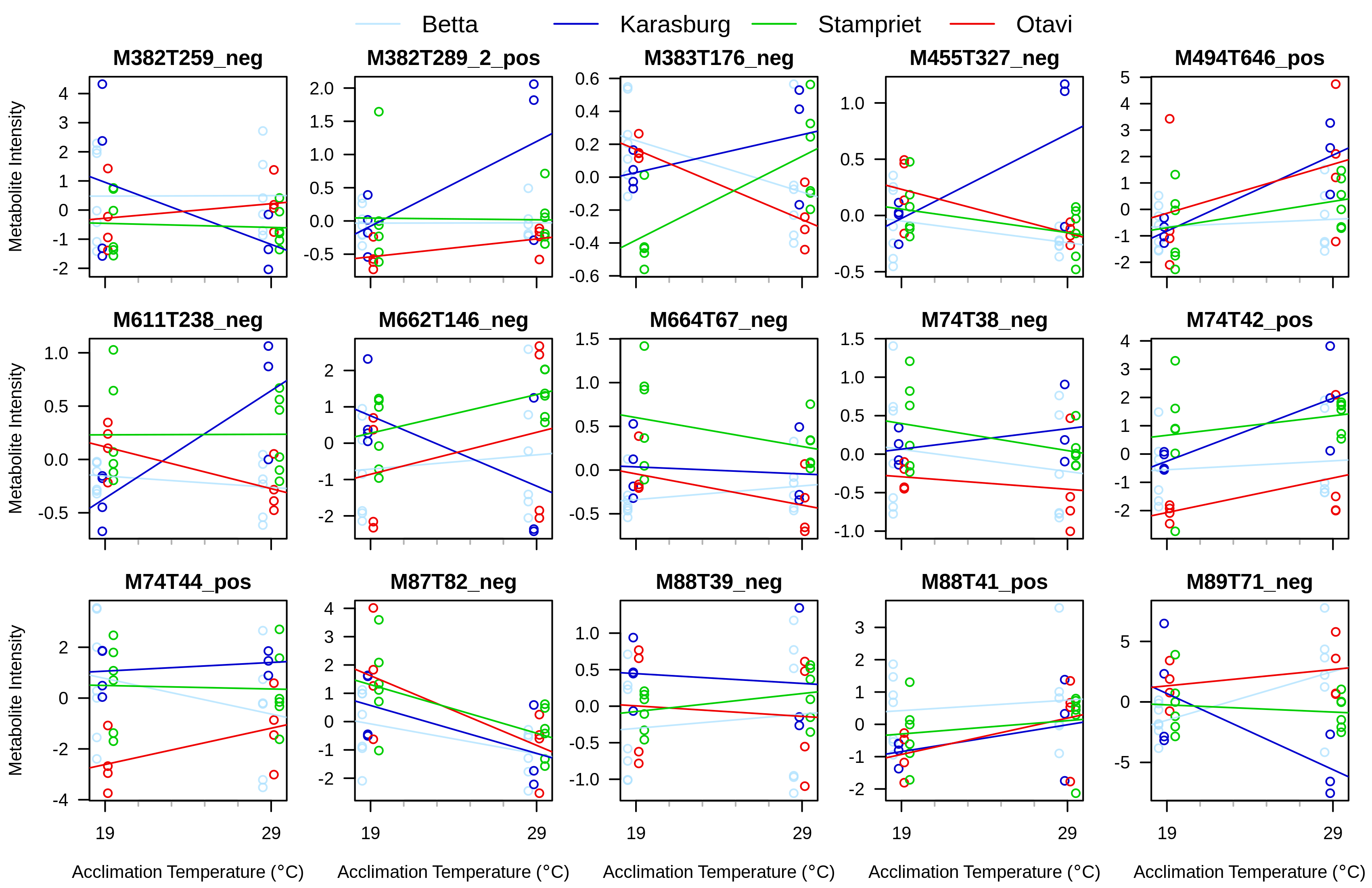


## Figure S17: PCA of metabolite LCMS data from the CCRTemp treatment

Principal component analysis of metabolites from the LC-MS analysis for spiders having undergone CCRTemp treatment, colored according to population and temperature acclimation. Only metabolites that showed population effects (a, c) or temperature effect (b,d) are plotted. Here the first three principal components are plottet. There is a tendency for Otavi (red) and Betta (light blue) to separate from Karasburg (blue) and Stampriet (green).


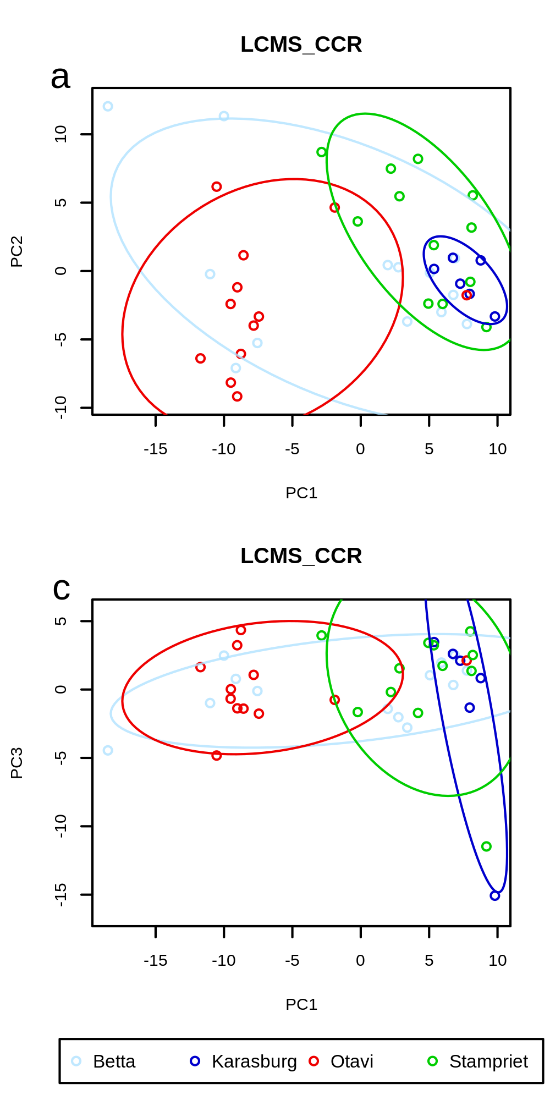

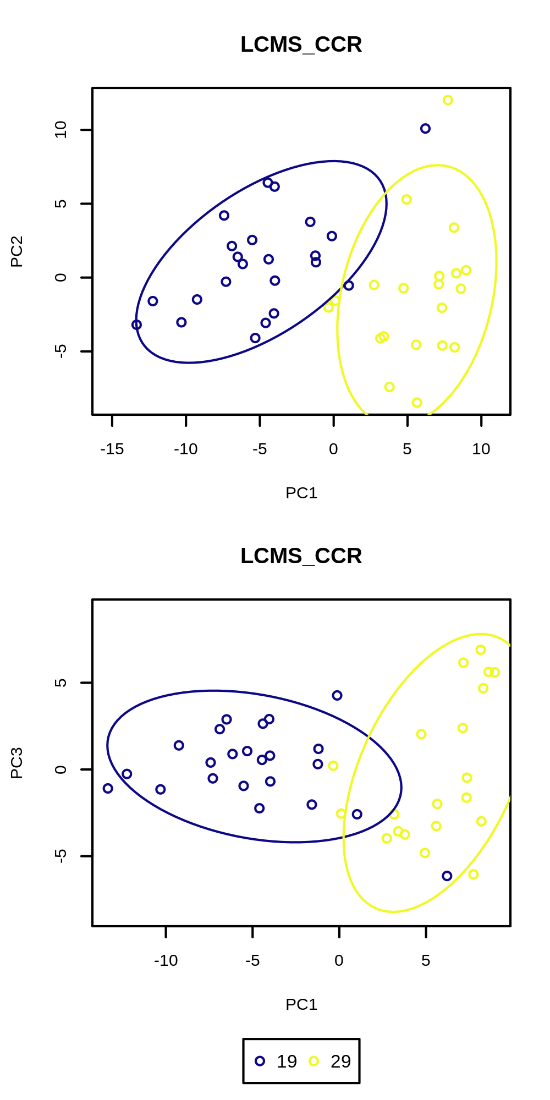


b

d

## Figure S18: PLS-DA analysis of metabolite data from the CCRTemp treatment

PLS-DA analysis of metabolites from the NMR analysis of spiders having been through CCRTemp testing. Points are metabolite intensities that show effect of either population (a, d) or temperature (b, c, e). Rows indicate metabolite extraction and analysis type: a, b) NMR aquatic extraction, c) NMR organic extraction and d, e) LC-MS.
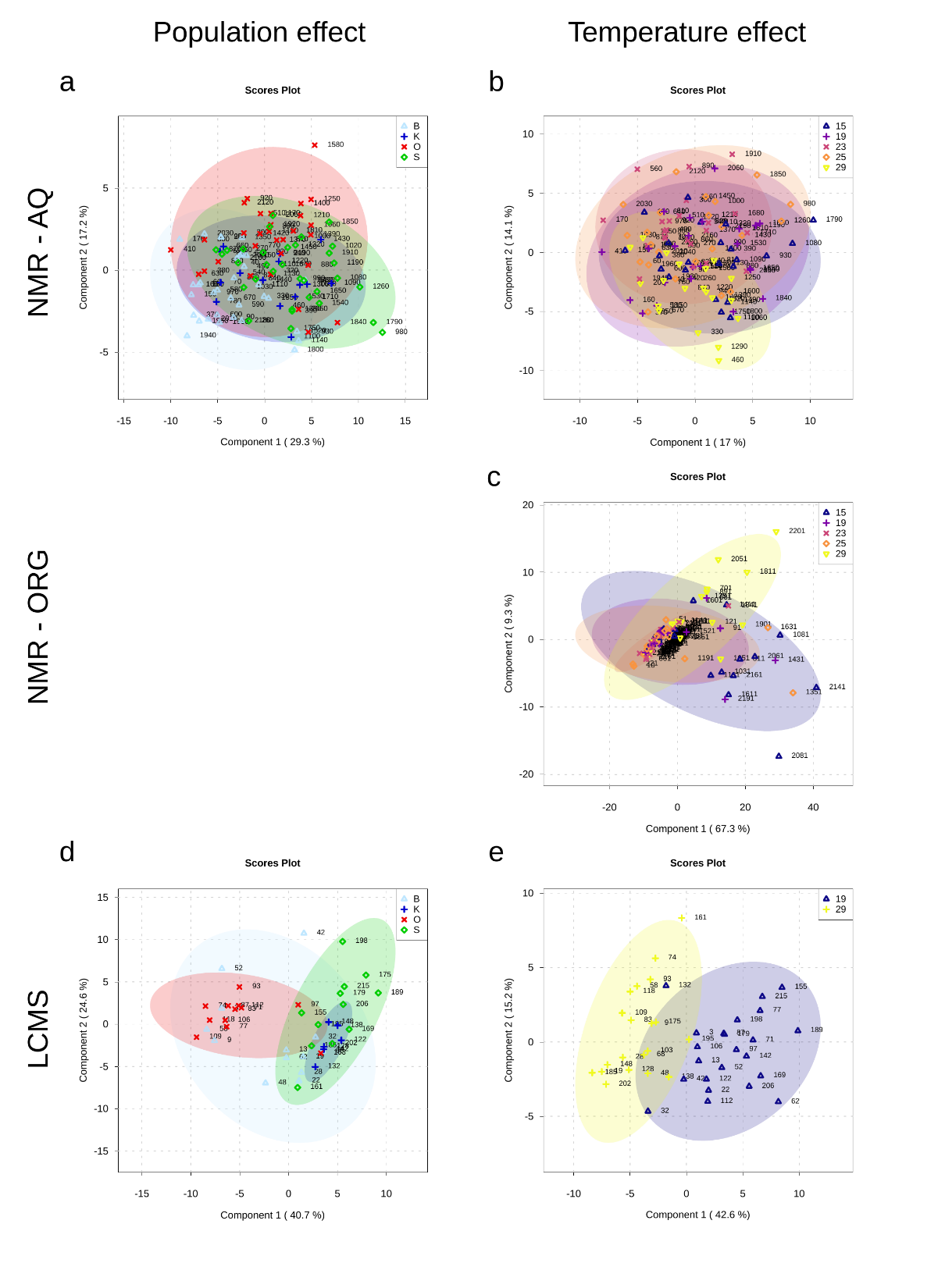


## Figure S19: Metabolite intensities for named LC-MS metabolites for CCRTemp tested spiders.

Metabolite intensities of all named LC-MS metabolites in CCRTemp performance tested spiders, giving a visualization of direction of change with temperature and difference between populations. Trendlines have been added for ease of interpretation.


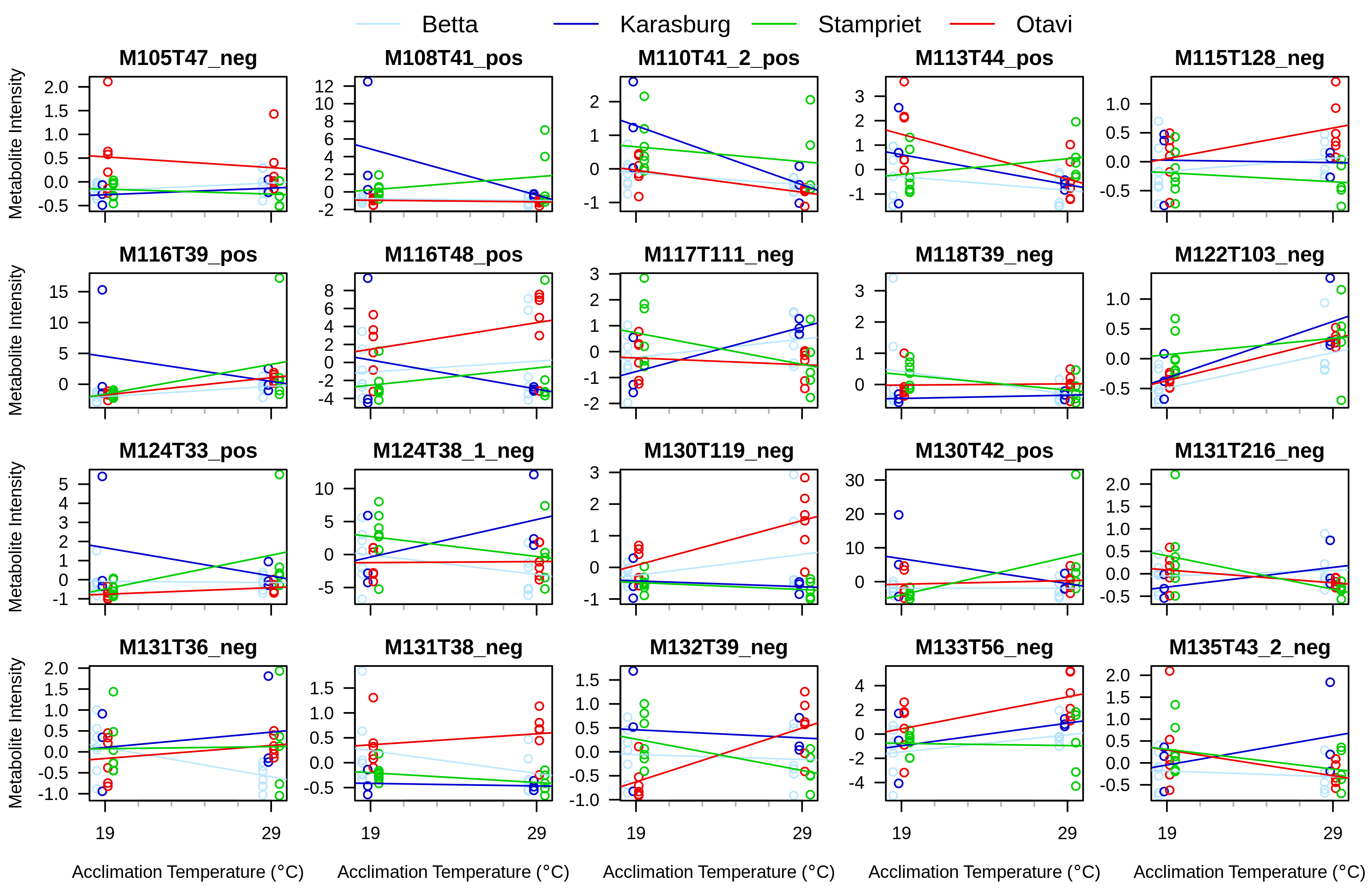


Figure S19 – continued: Metabolite intensities for named LC-MS metabolites for CCRTemp tested spiders.


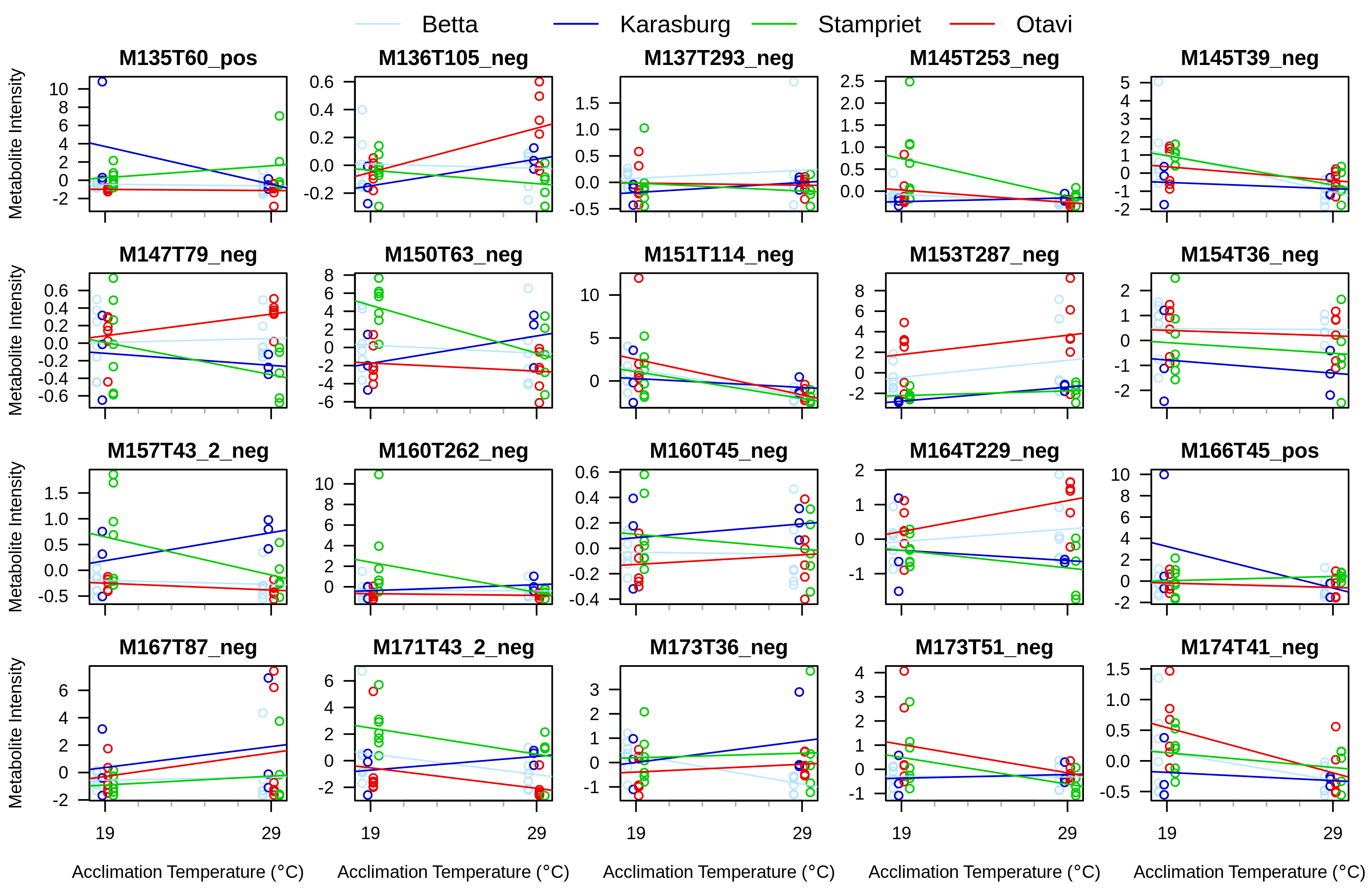


Figure S19 – continued: Metabolite intensities for named LC-MS metabolites for CCRTemp tested spiders.


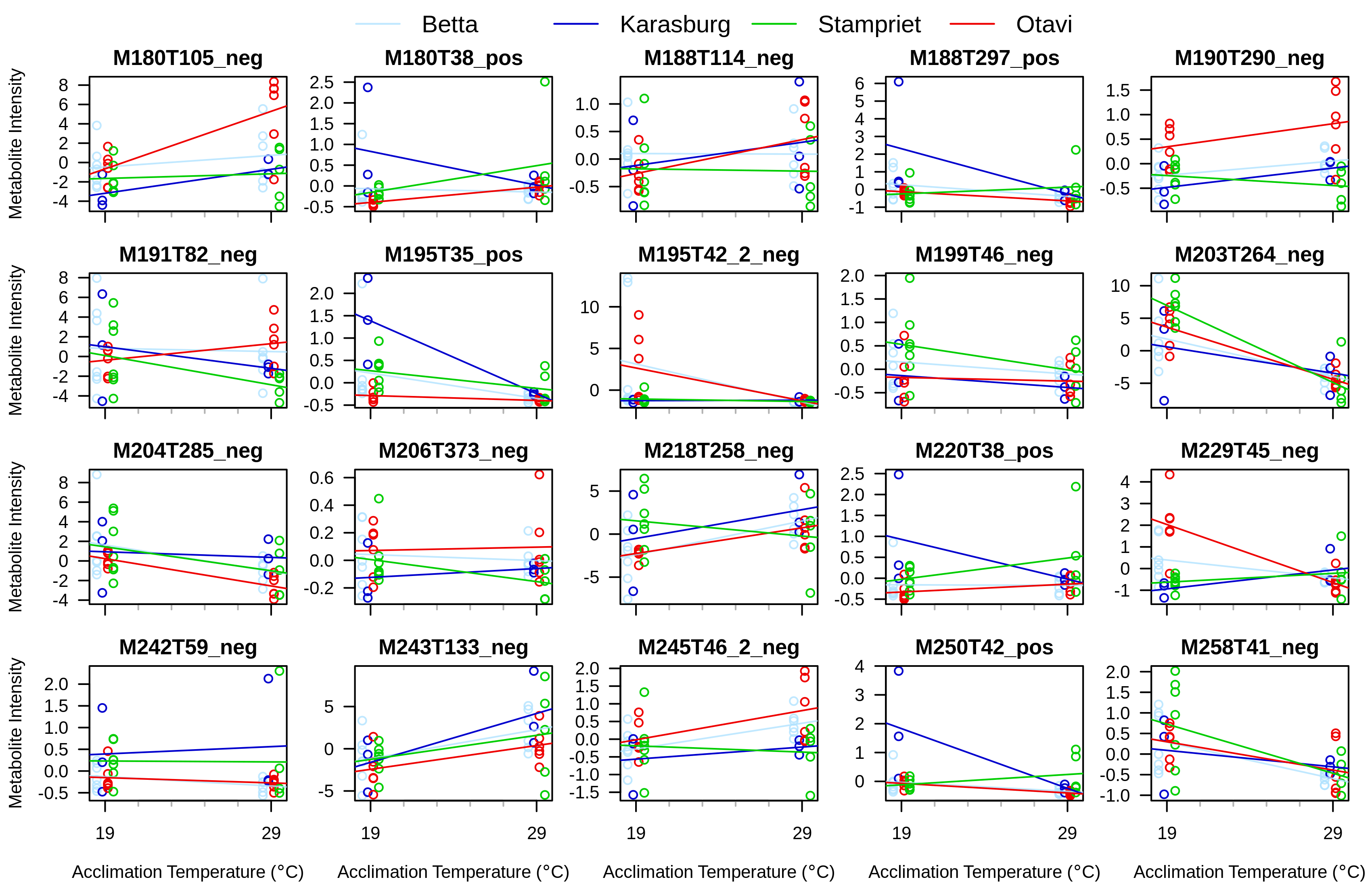


Figure S19 – continued: Metabolite intensities for named LC-MS metabolites for CCRTemp tested spiders.


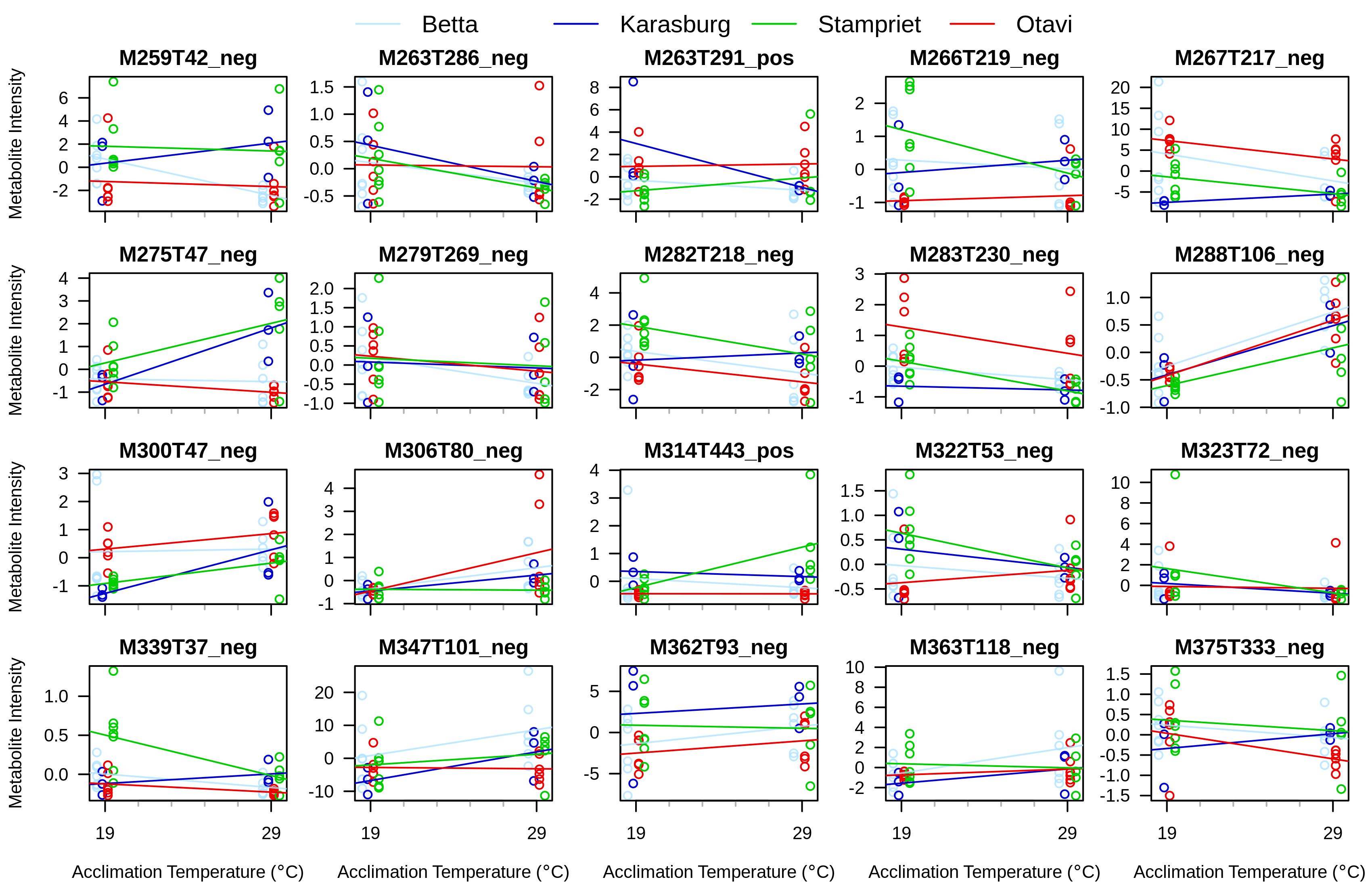


Figure S19 – continued: Metabolite intensities for named LC-MS metabolites for CCRTemp tested spiders.


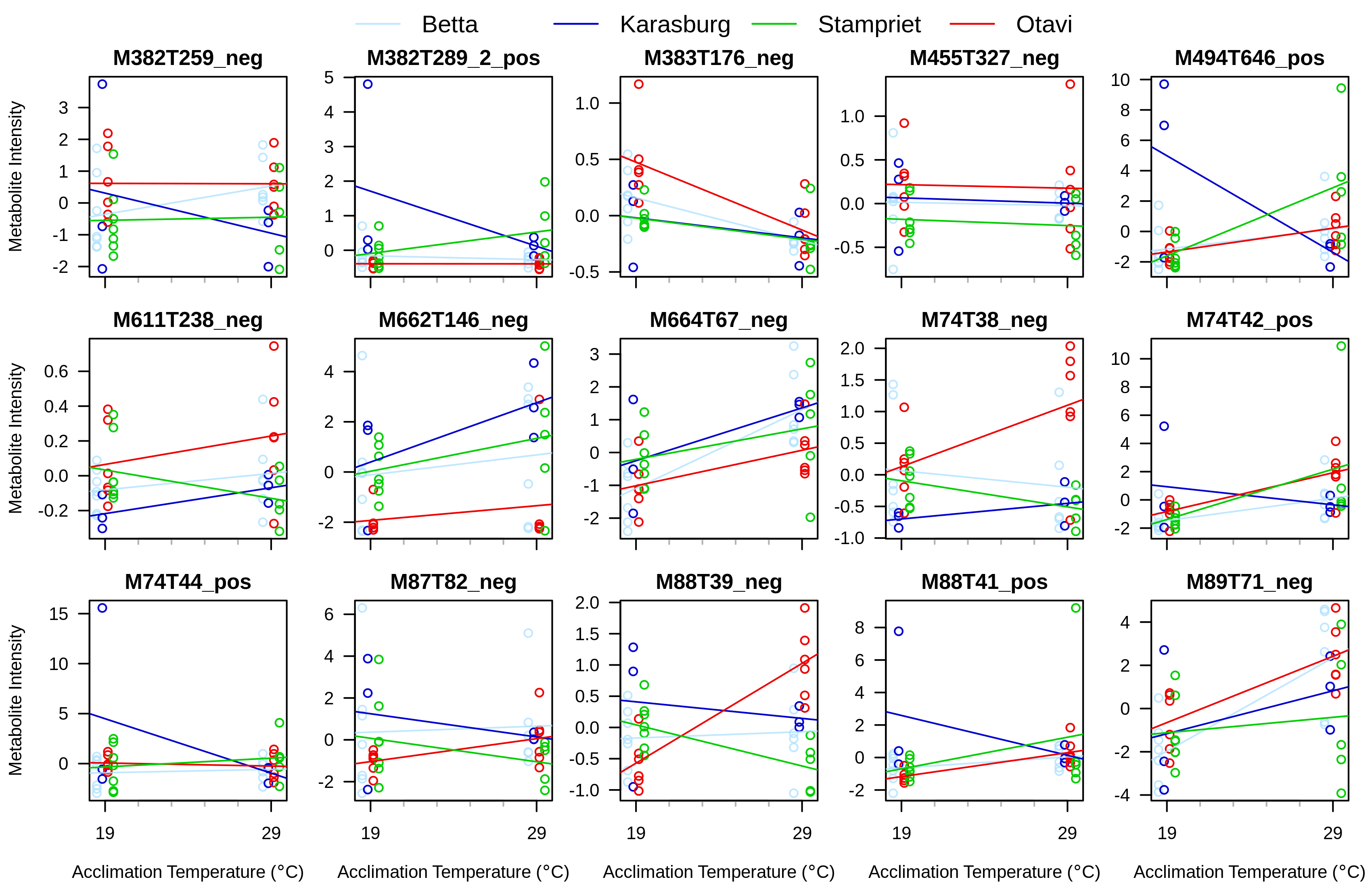


## Figure S20: PCA of metabolite NMR-Aq data from the CTmax treatment

Principal Component Analysis of Metabolites from NMR from spiders having gone through CTmax treatment, colored according to population and temperature acclimation. Only metabolites that showed population effects (a, c) or temperature effect (b,d) are plotted. Here the first three principal components are plottet.


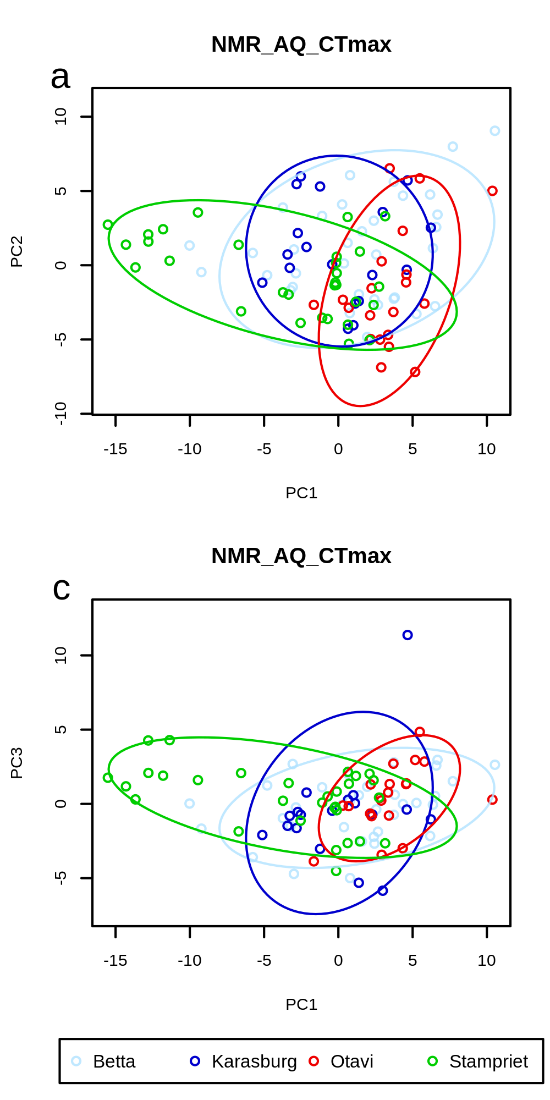

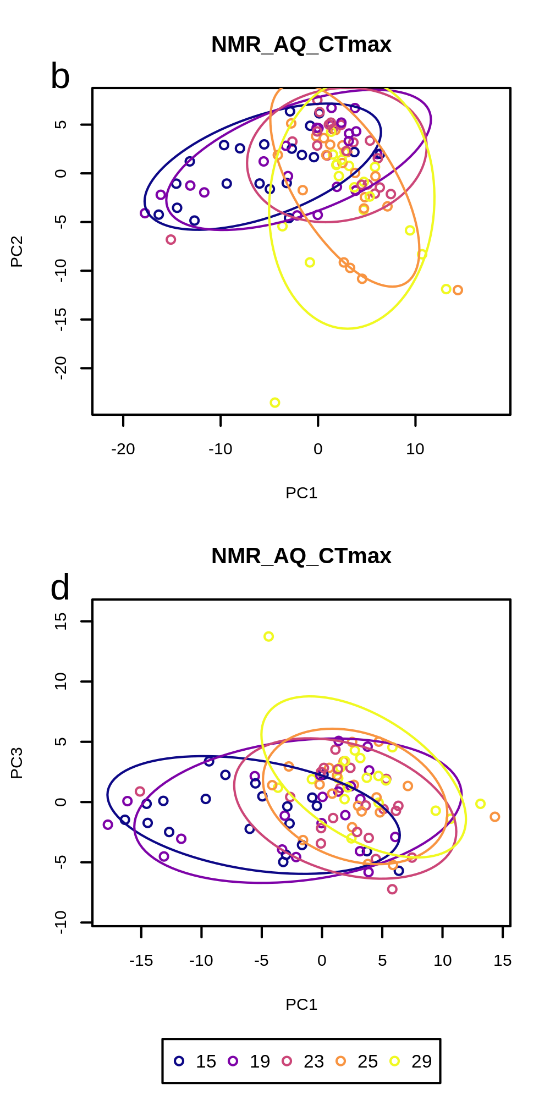


## Figure S21: PCA of metabolite NMR-Aq data from the CCRTemp treatment

Principal Component Analysis of Metabolites from NMR from spiders having gone through CCR treatment, colored according to population and temperature acclimation. Only metabolites that showed population effects (a, c) or temperature effect (b,d) are plotted. Here the first three principal components are plottet.


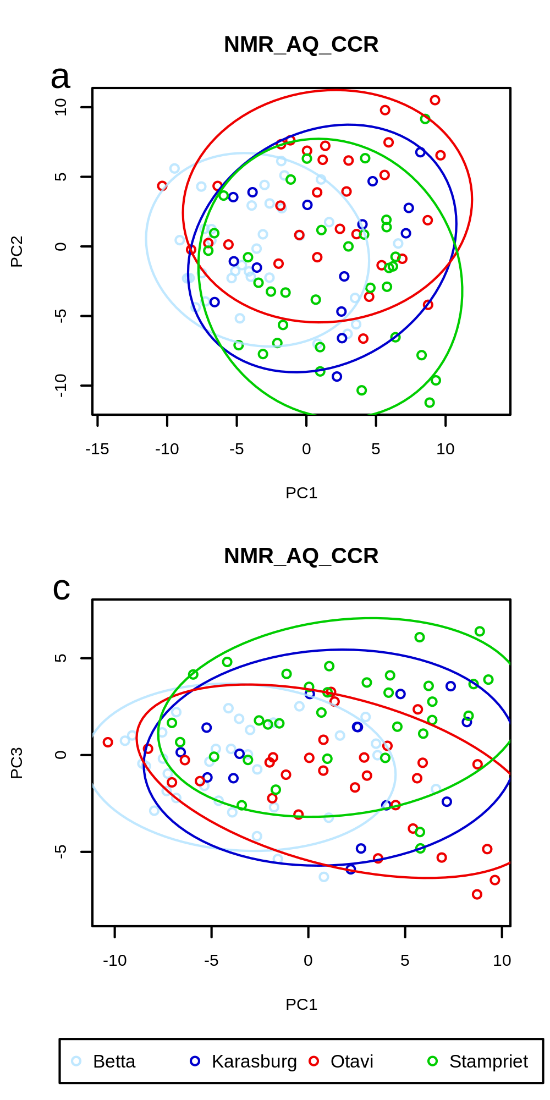

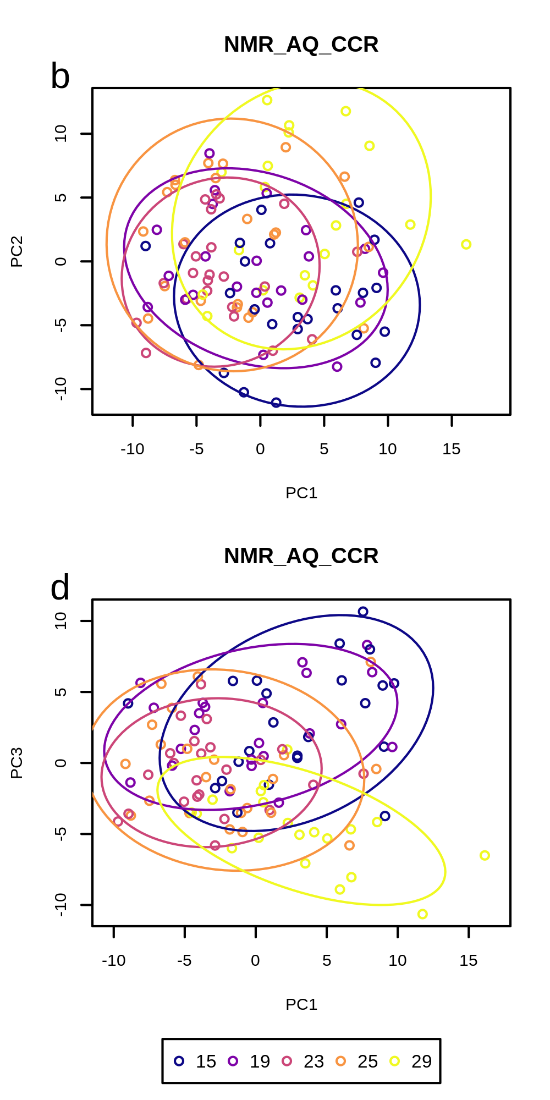


## Figure S22: Number of ASVs with responses to population and temperature in microbiome

Barplot with the number of microbial ASVs showing population and temperature responses in S. dumicola. The total number of ASVs passing the filtering criterion is 78.

**
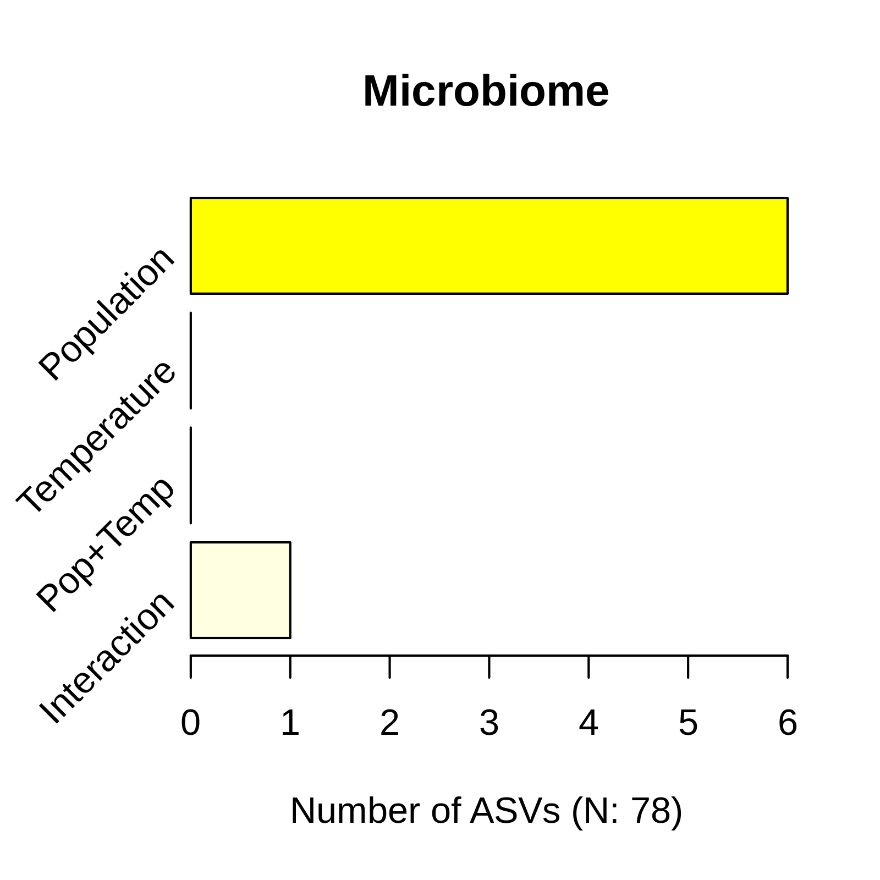
**

## Figure S23: Gene expression with similar population specific acclimation responses as the heat tolerance phenotype

Genes with expression levels showing similar population-specific trends as that of Heat tolerance (CTmax). Genes presented in these graphs show a response suggesting an involvement in the observed population dependent heat tolerance, thus linking gene expression to the expressed phenotype.


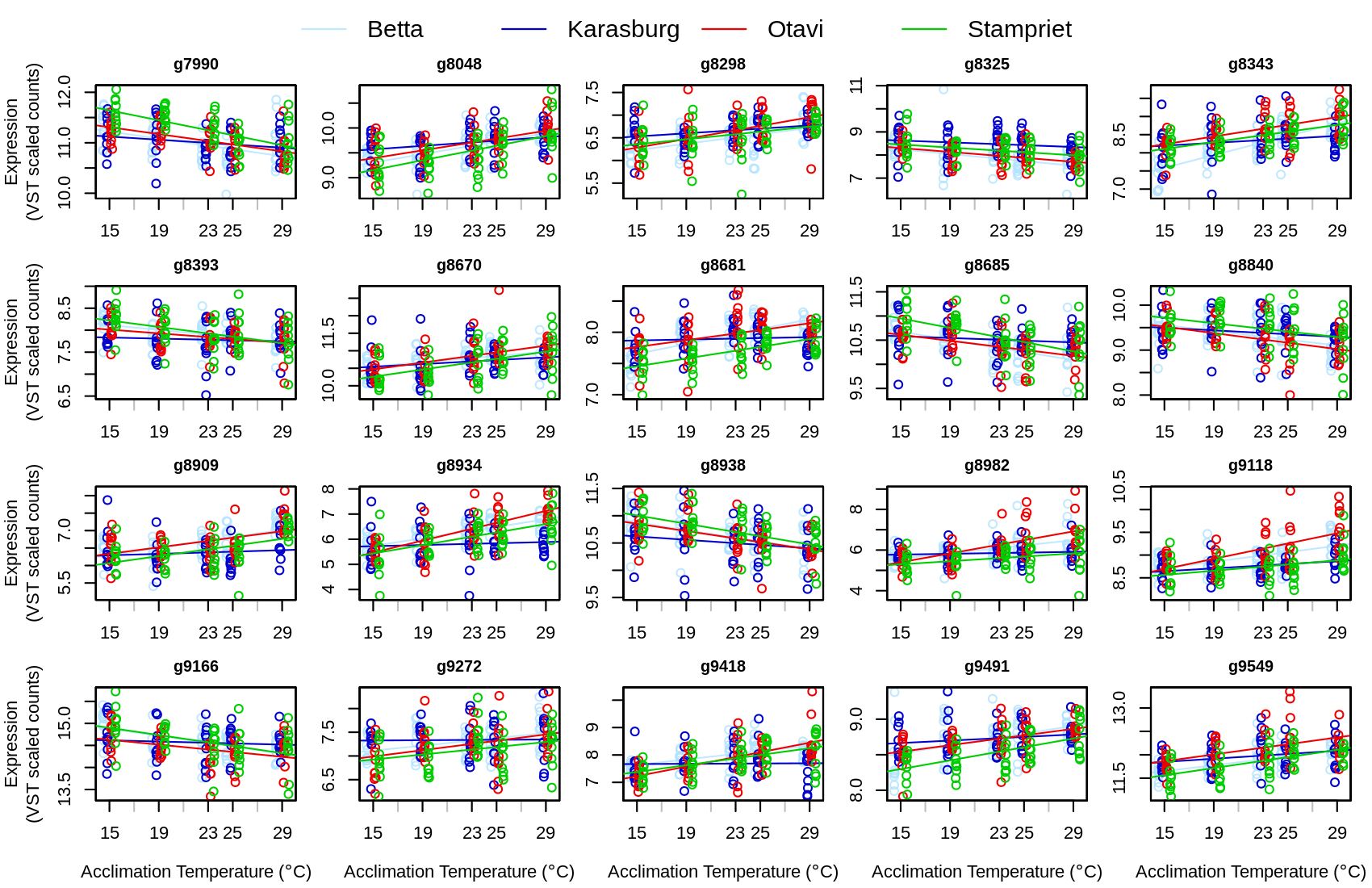


Figure S23 – continued: Gene expression with similar population specific acclimation responses as the heat tolerance phenotype


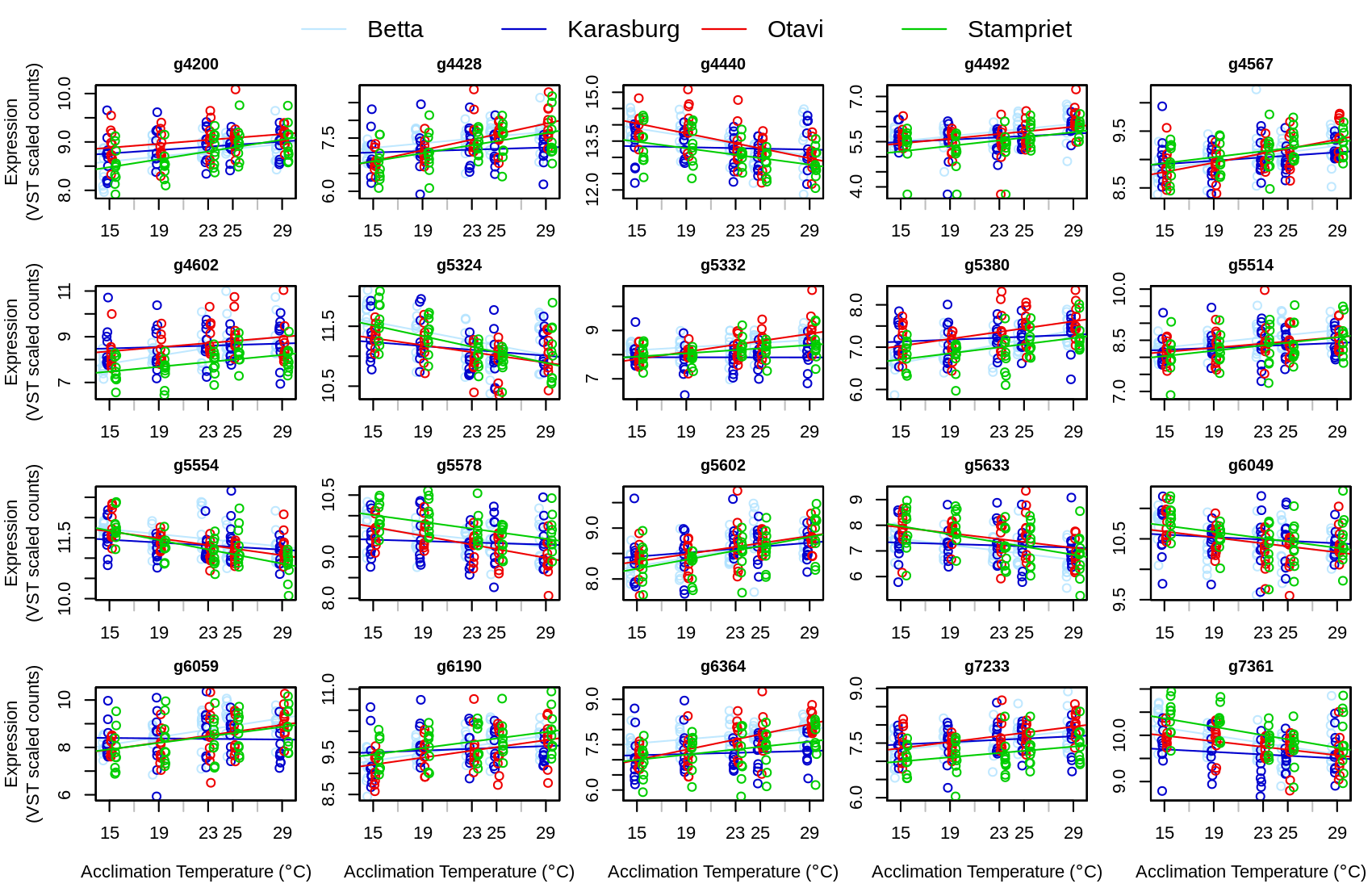


Figure S23 – continued: Gene expression with similar population specific acclimation responses as the heat tolerance phenotype


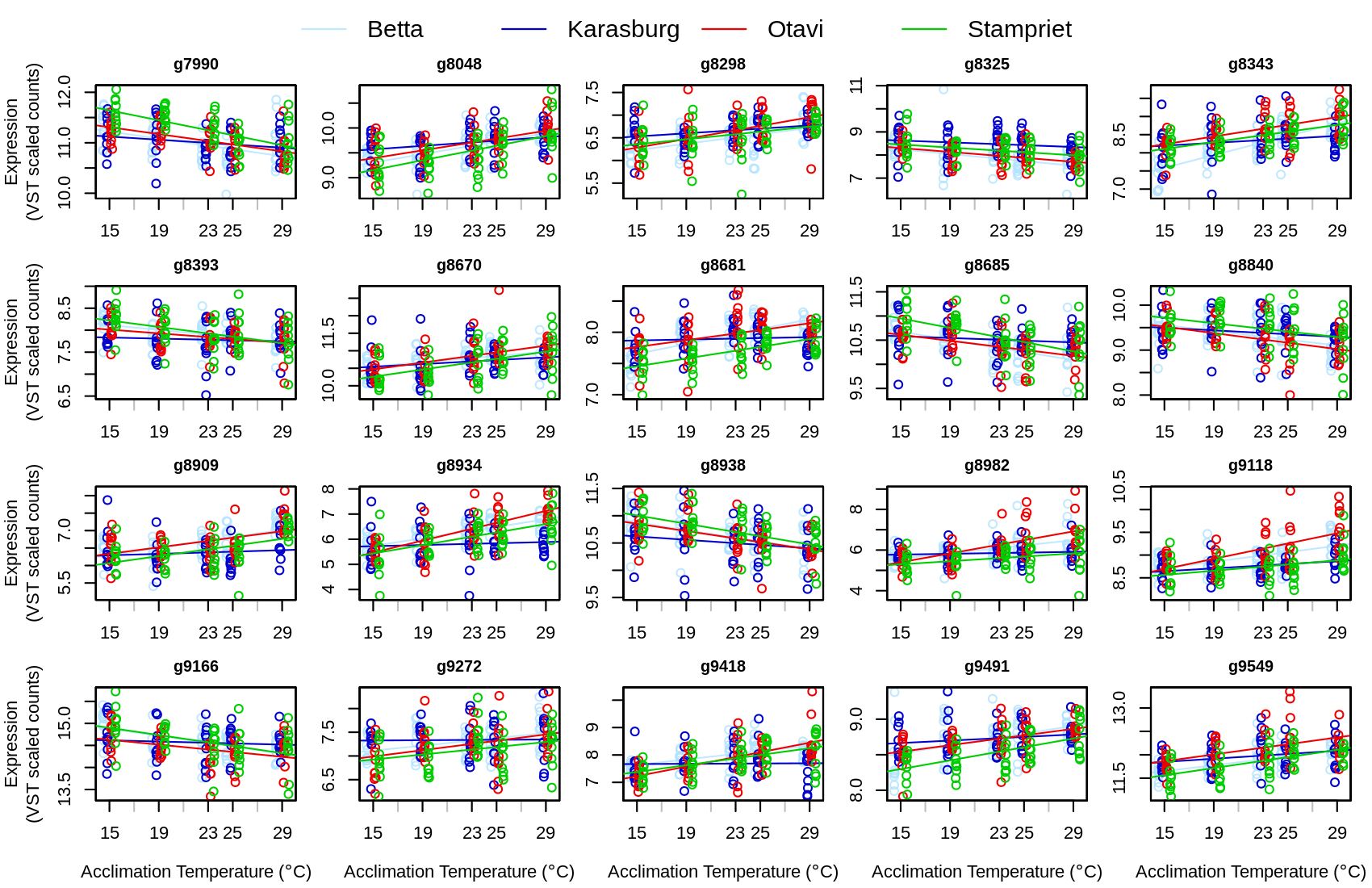


Figure S23 – continued: Gene expression with similar population specific acclimation responses as the heat tolerance phenotype


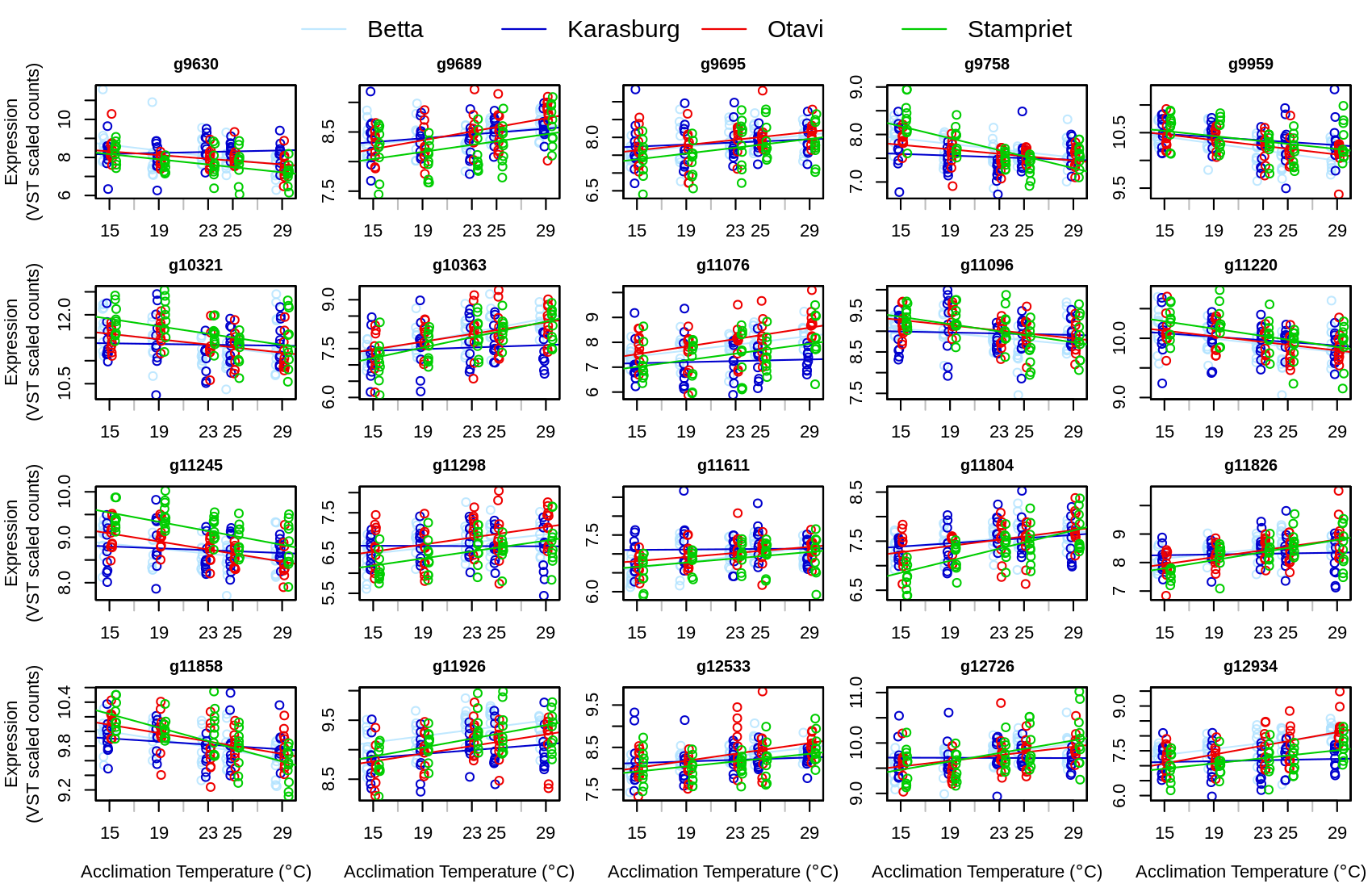


Figure S23 – continued: Gene expression with similar population specific acclimation responses as the heat tolerance phenotype


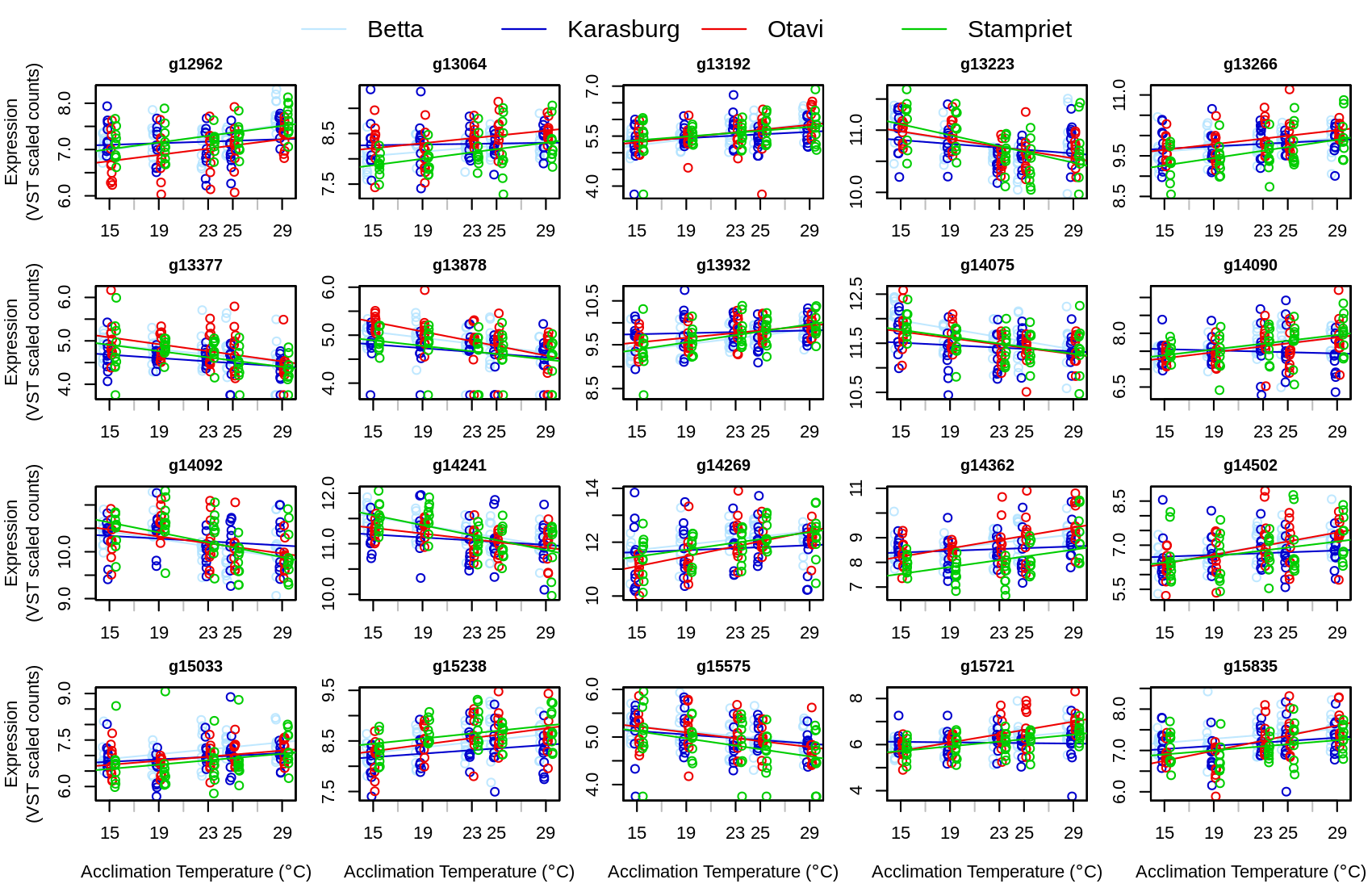


Figure S23 – continued: Gene expression with similar population specific acclimation responses as the heat tolerance phenotype


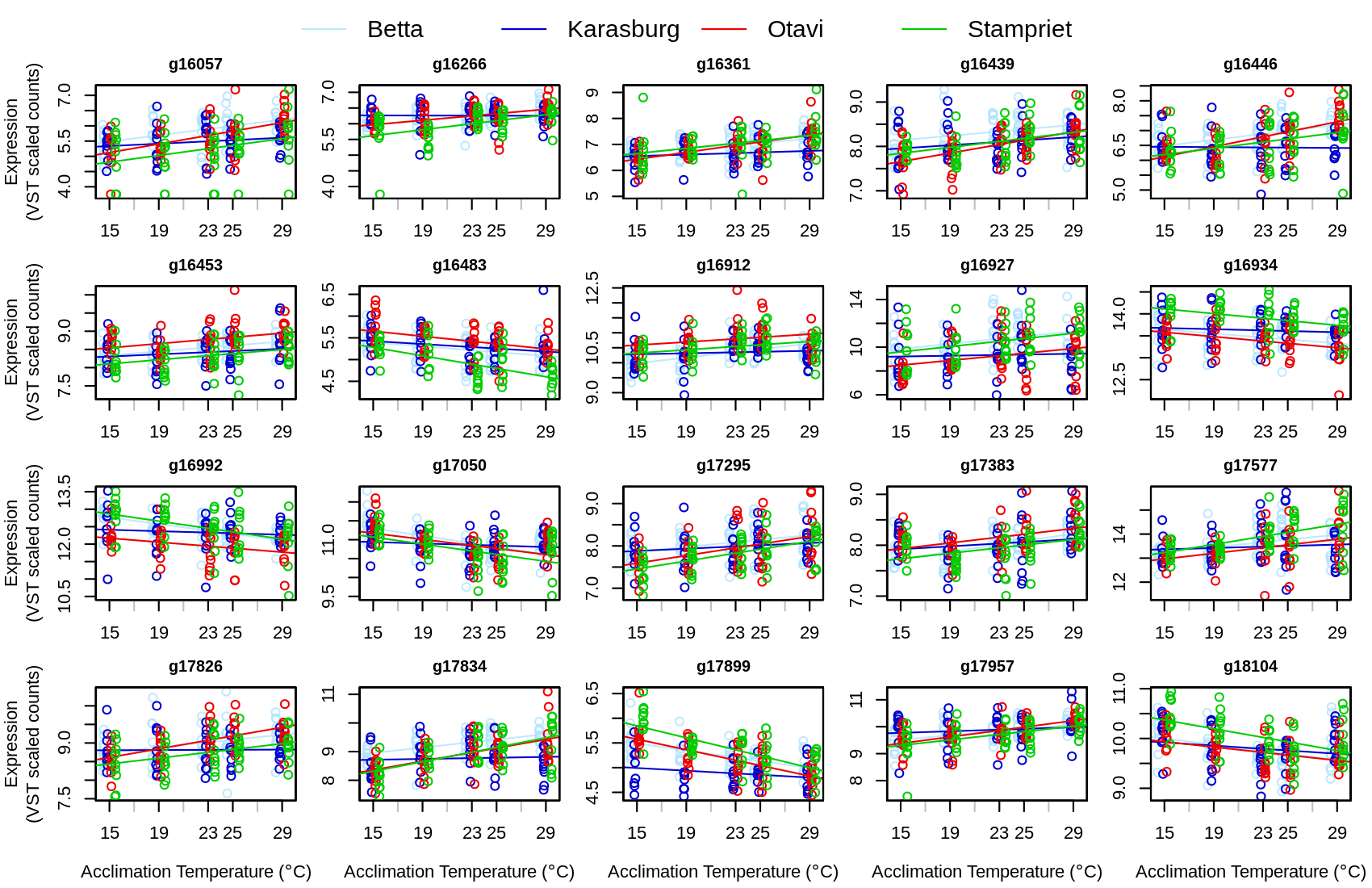


Figure S23 – continued: Gene expression with similar population specific acclimation responses as the heat tolerance phenotype


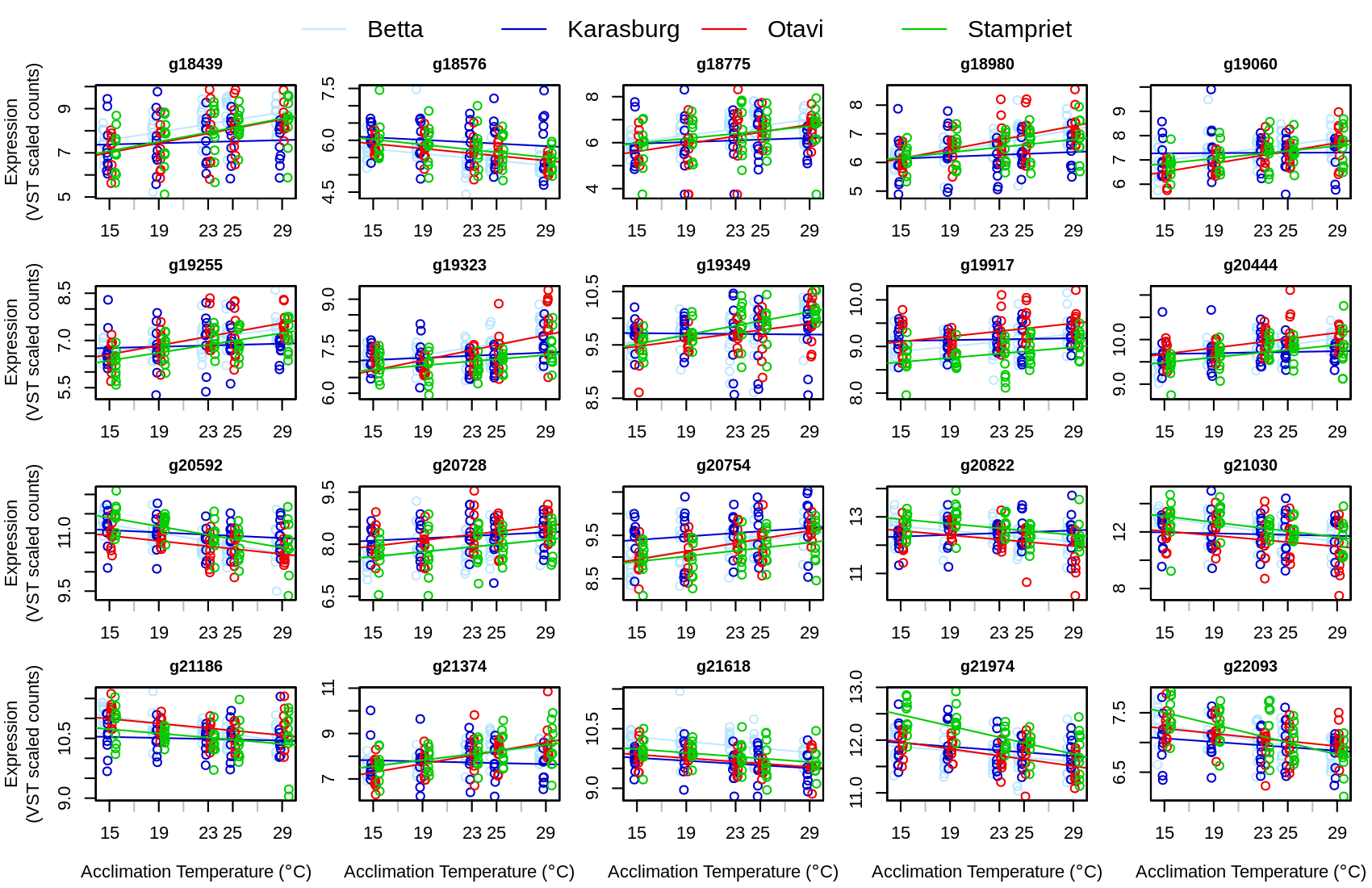


Figure S23 – continued: Gene expression with similar population specific acclimation responses as the heat tolerance phenotype


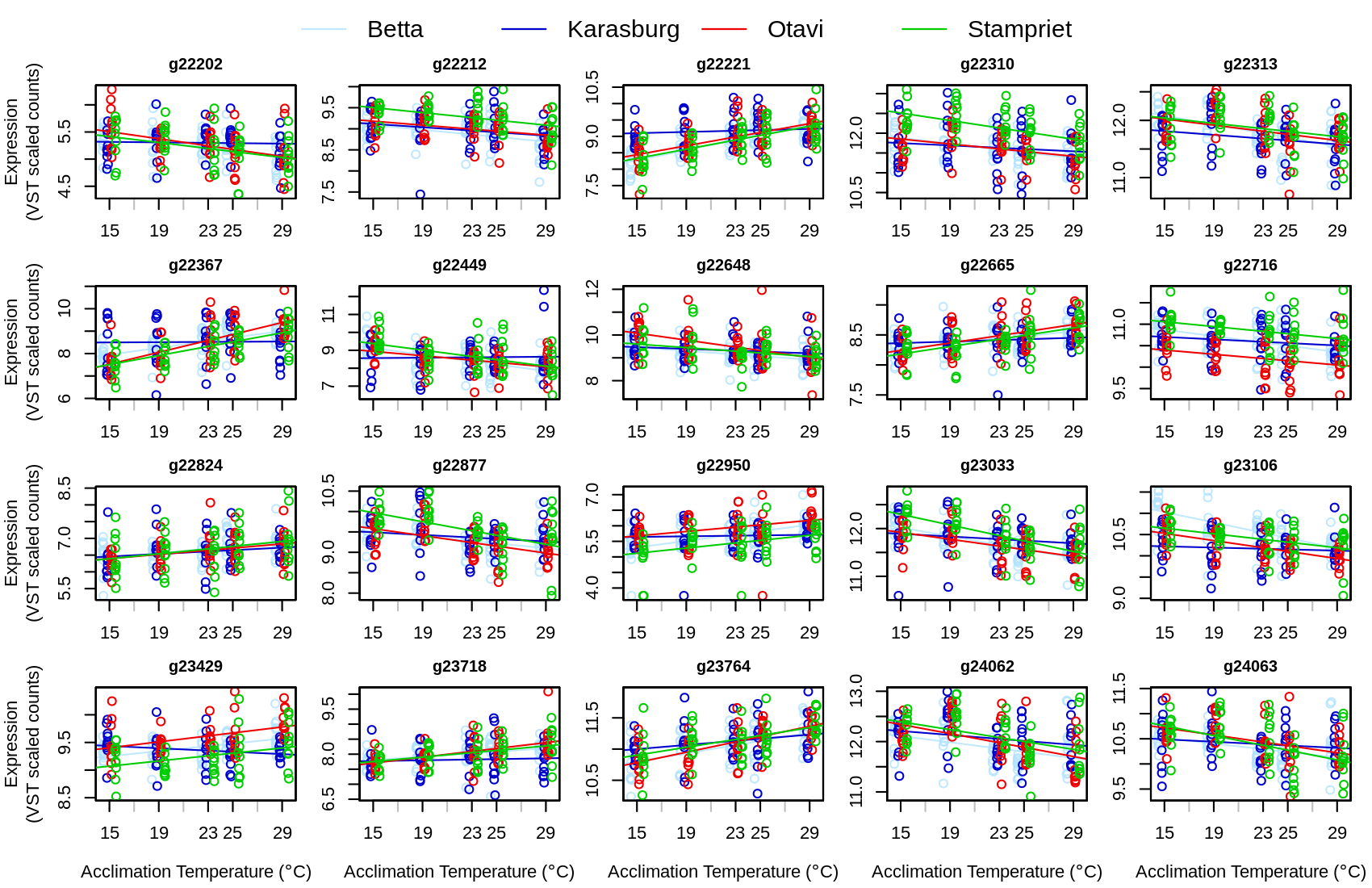


Figure S23 – continued: Gene expression with similar population specific acclimation responses as the heat tolerance phenotype


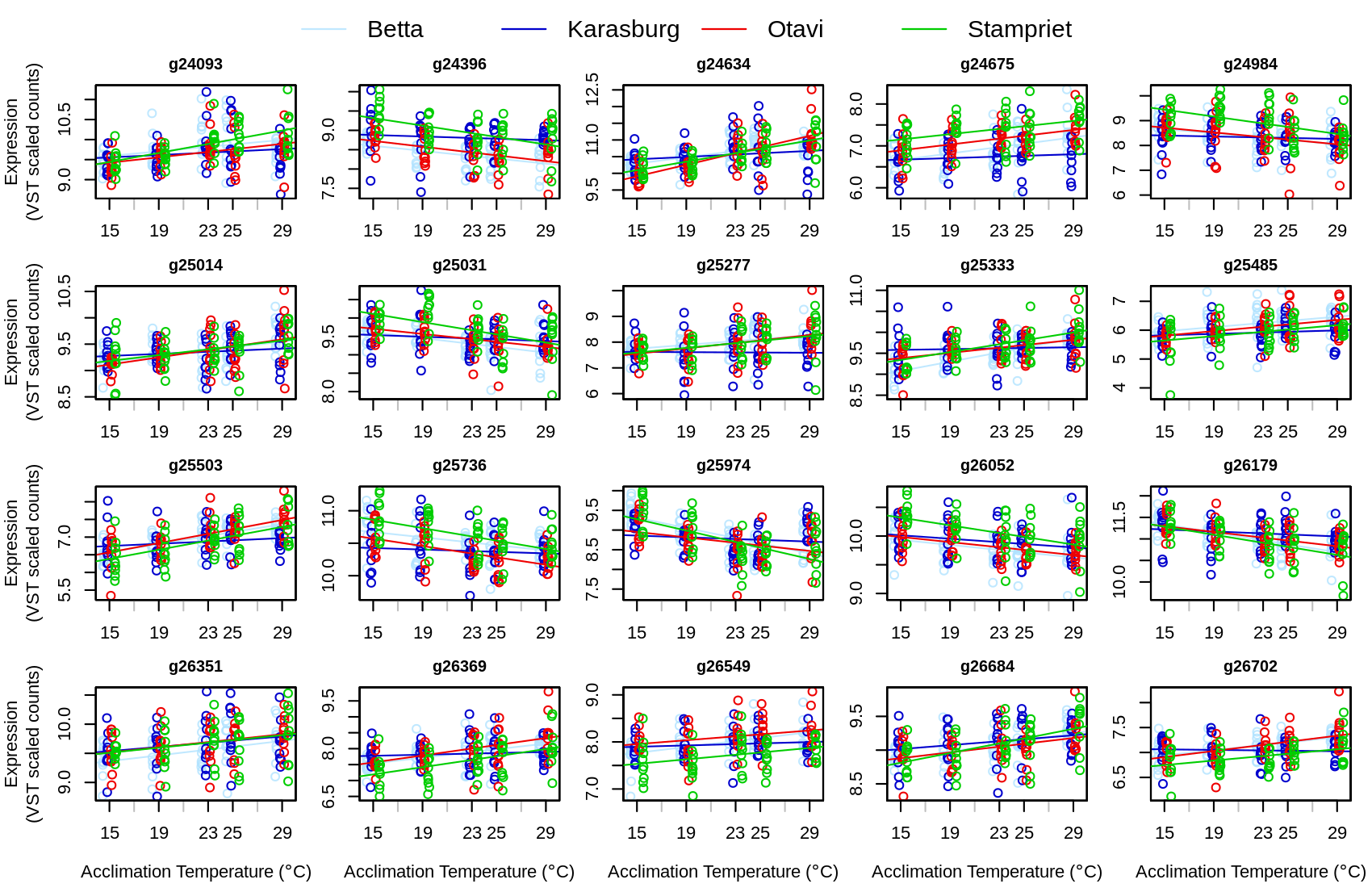


Figure S23 – continued: Gene expression with similar population specific acclimation responses as the heat tolerance phenotype


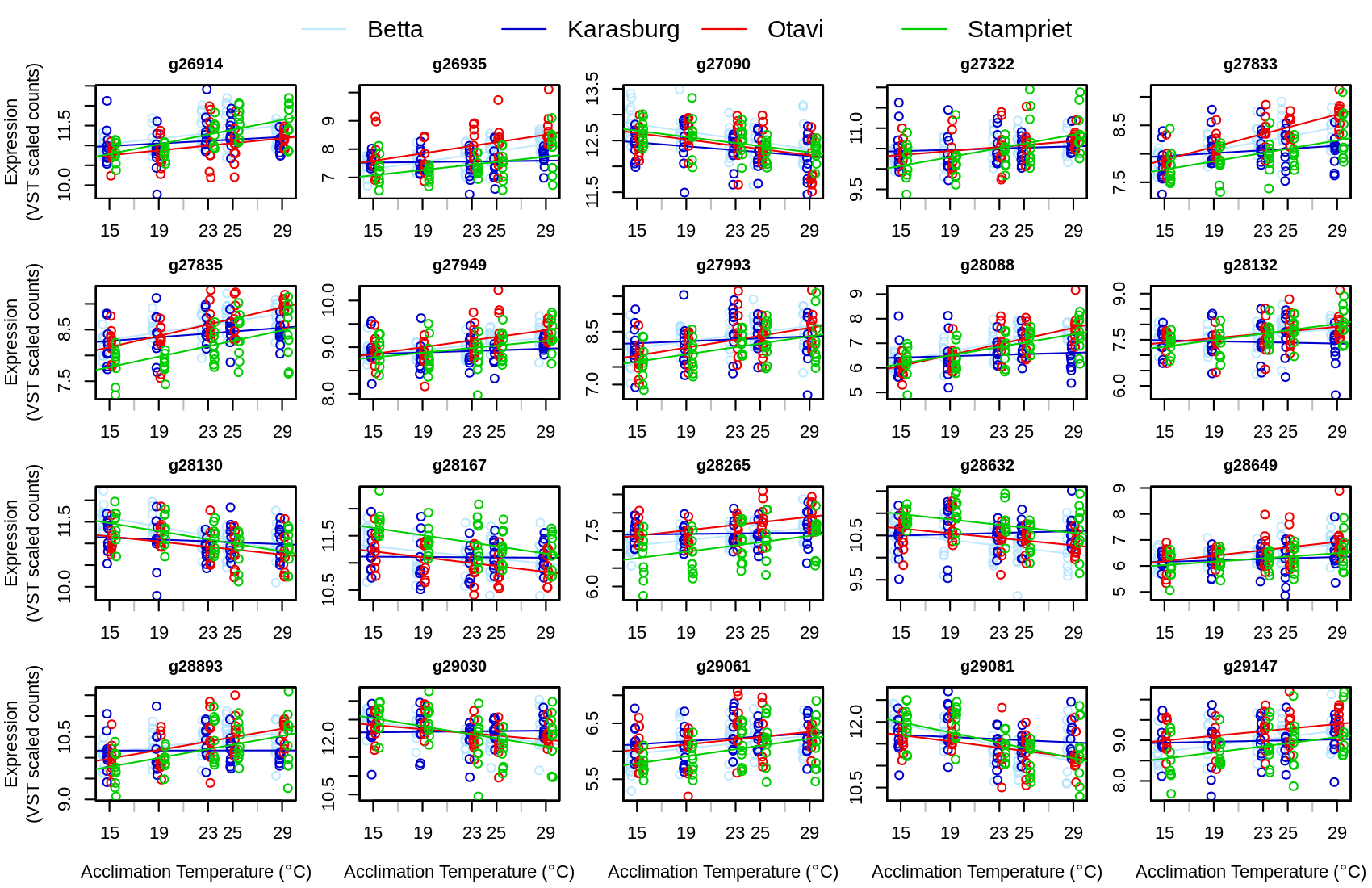


Figure S23 – continued: Gene expression with similar population specific acclimation responses as the heat tolerance phenotype


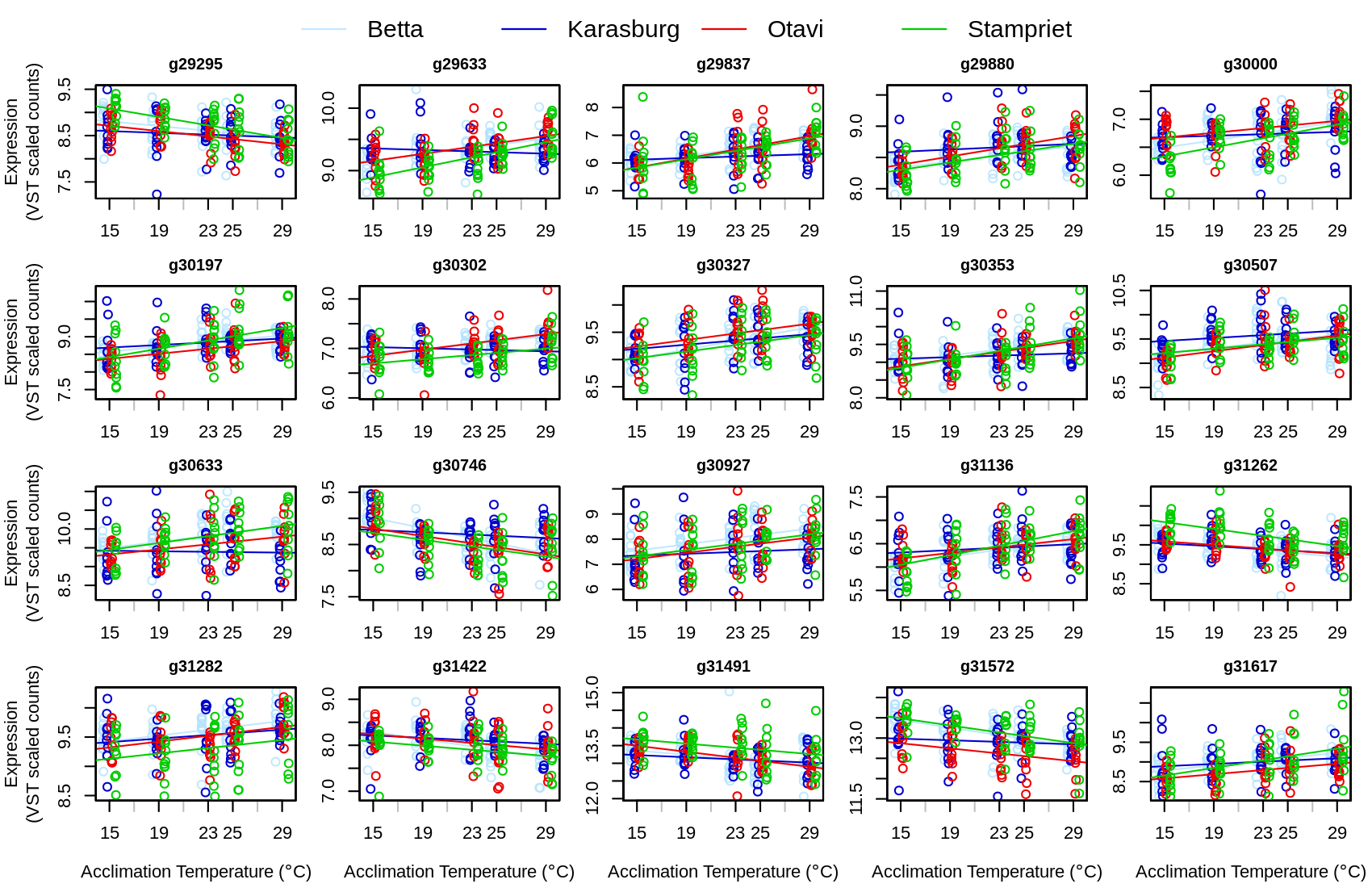


Figure S23 – continued: Gene expression with similar population specific acclimation responses as the heat tolerance phenotype


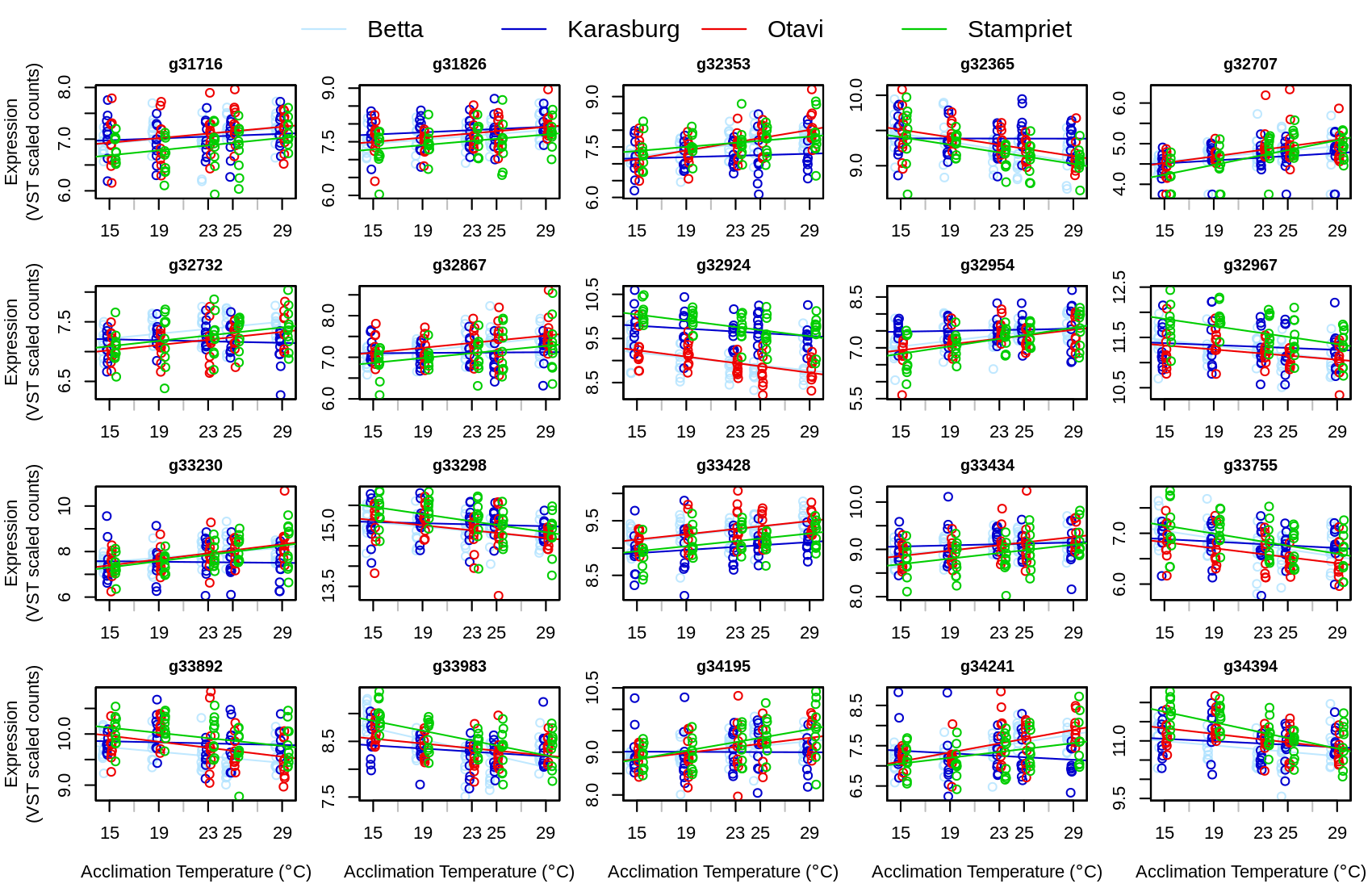


Figure S23 – continued: Gene expression with similar population specific acclimation responses as the heat tolerance phenotype


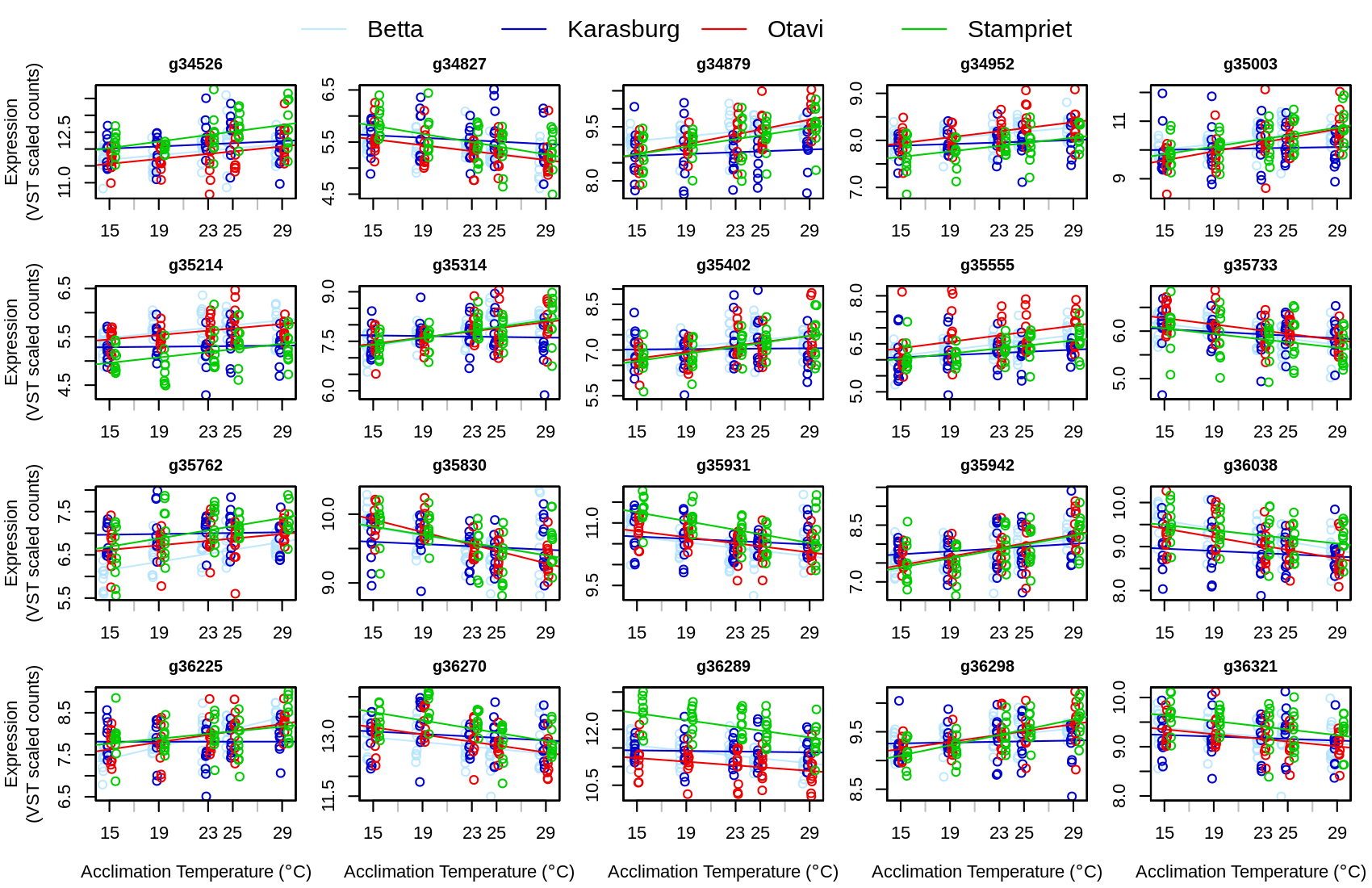


Figure S23 – continued: Gene expression with similar population specific acclimation responses as the heat tolerance phenotype


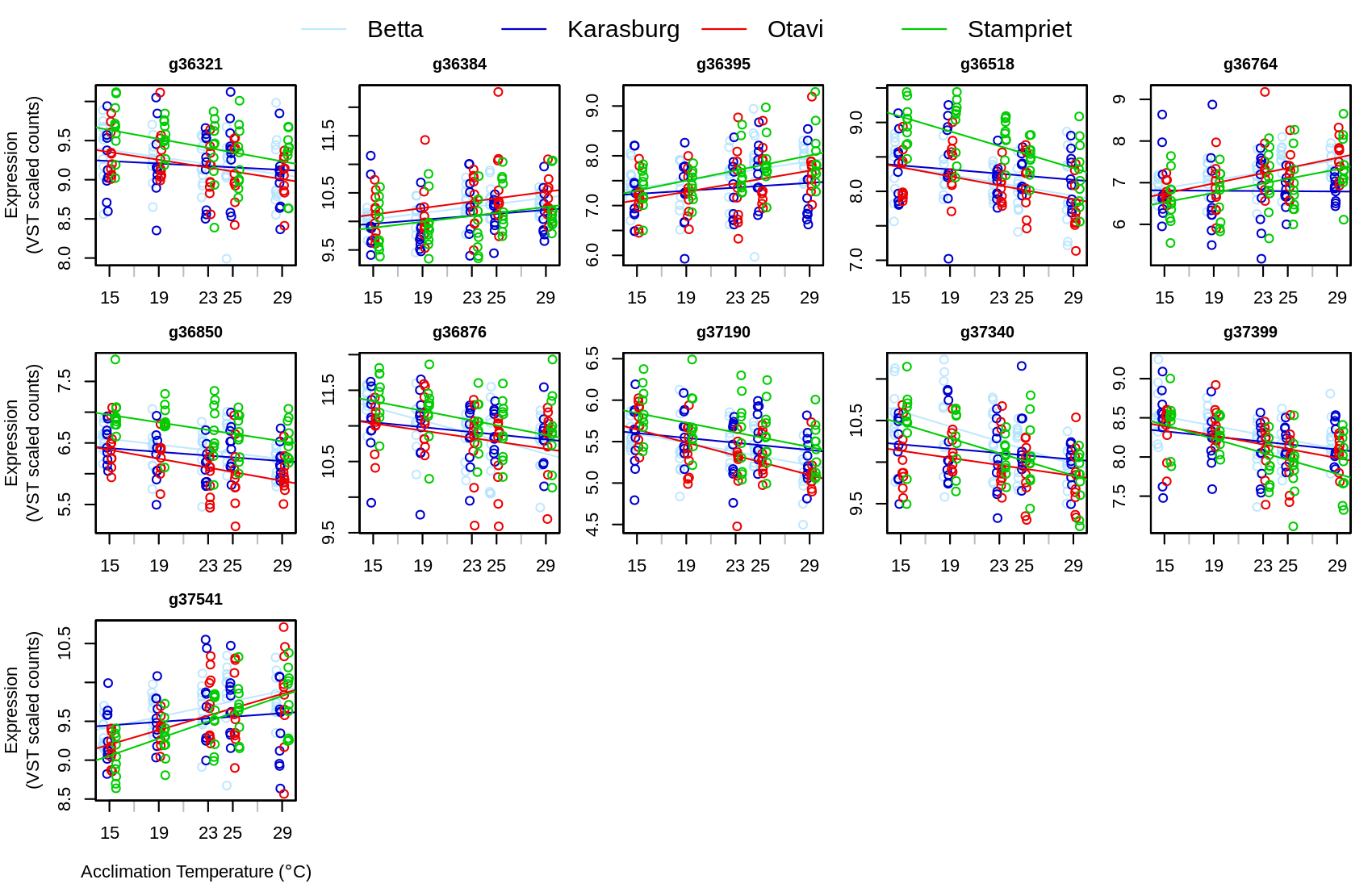


## Figure S24: Gene expression with similar population specific acclimation responses as the cold tolerance phenotype

Genes with expression levels showing similar population-specific trends as that of cold tolerance (CCRTemp). Genes presented in these graphs show a response suggesting a clearer involvement in the observed population dependent cold tolerance, linking gene expression to the expressed phenotype.


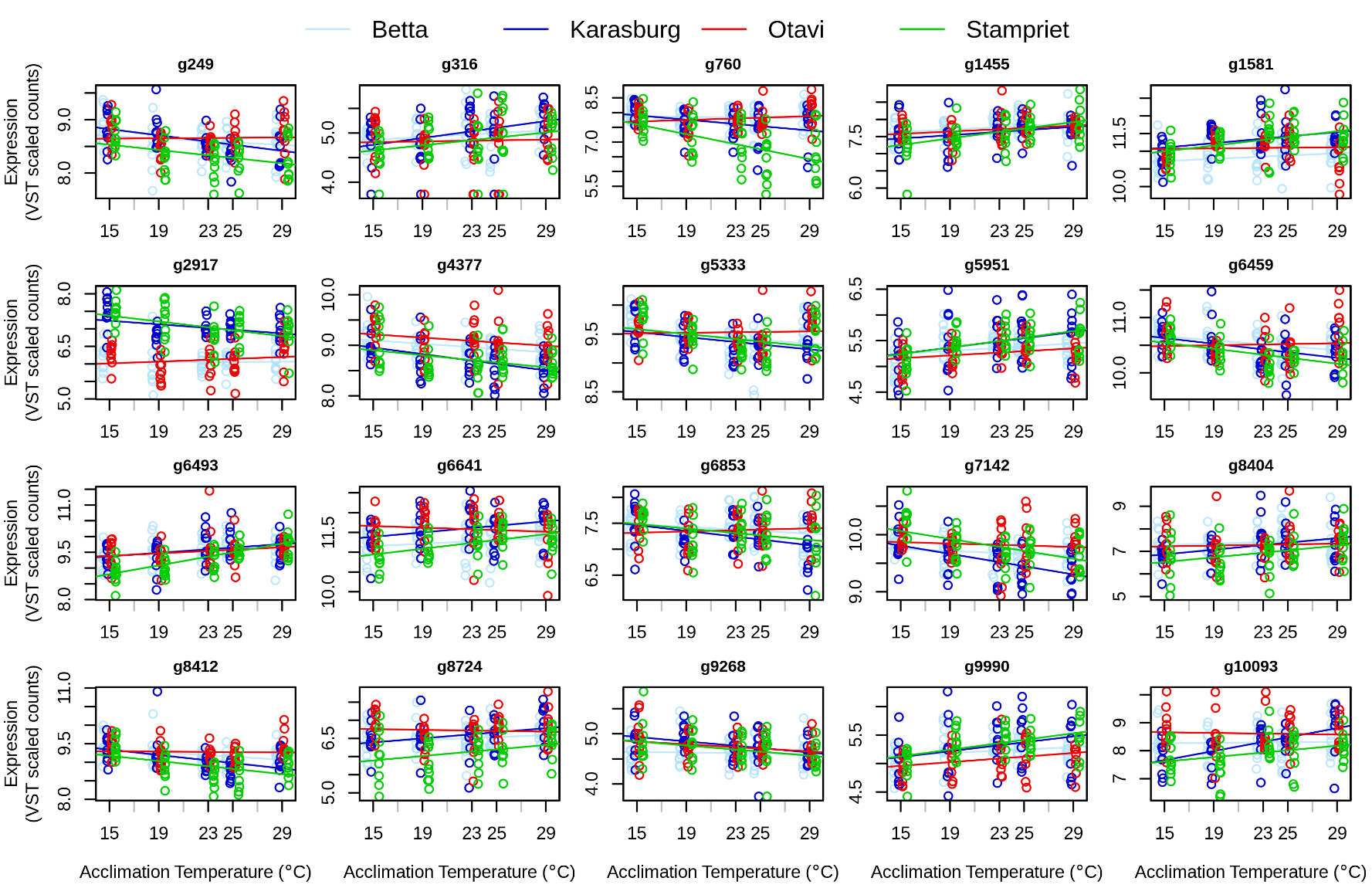


Figure S24 - continued: Gene expression with similar population specific acclimation responses as the cold tolerance phenotype


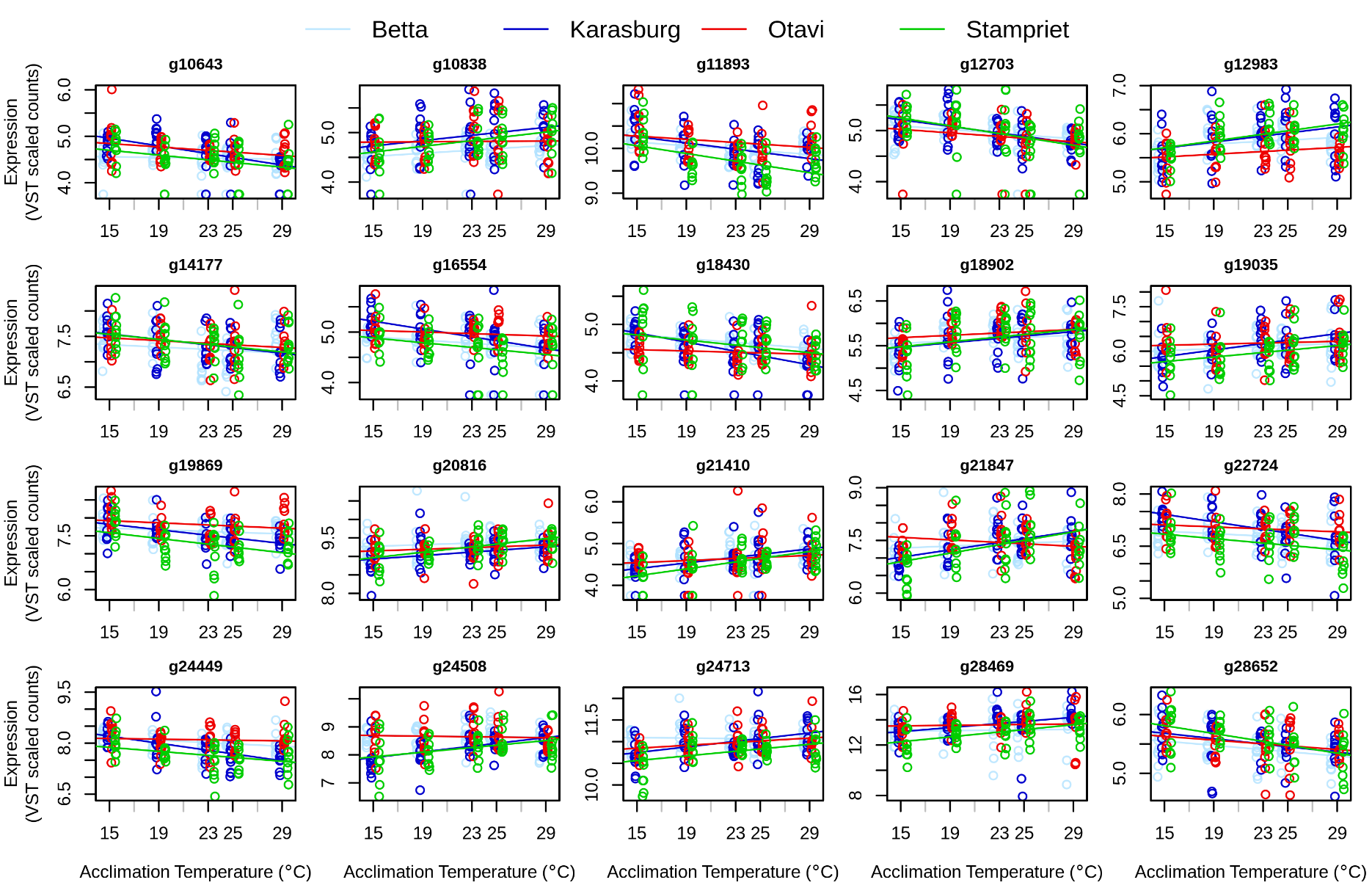


Figure S24 - continued: Gene expression with similar population specific acclimation responses as the cold tolerance phenotype


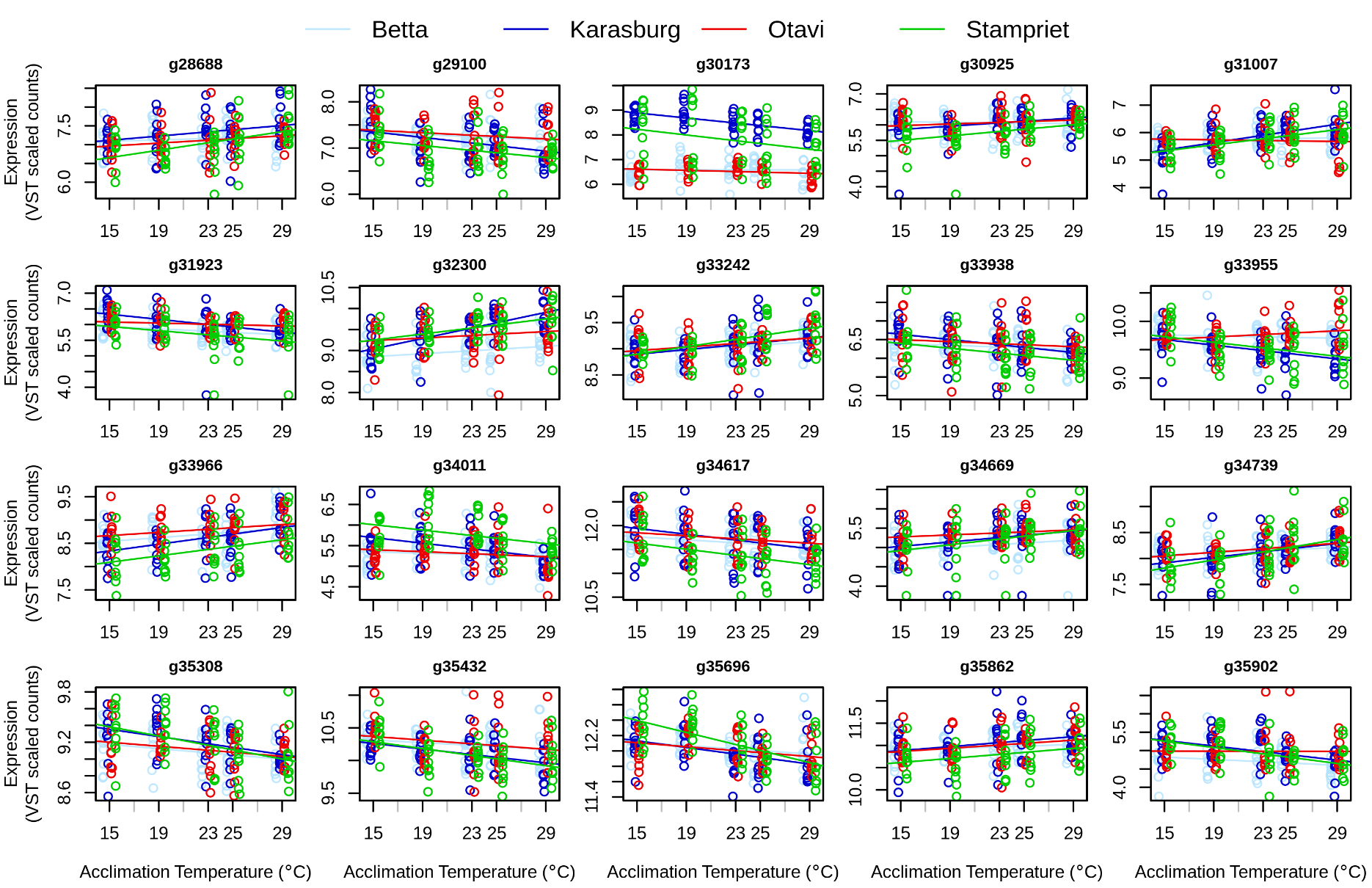


Figure S24 - continued: Gene expression with similar population specific acclimation responses as the cold tolerance phenotype


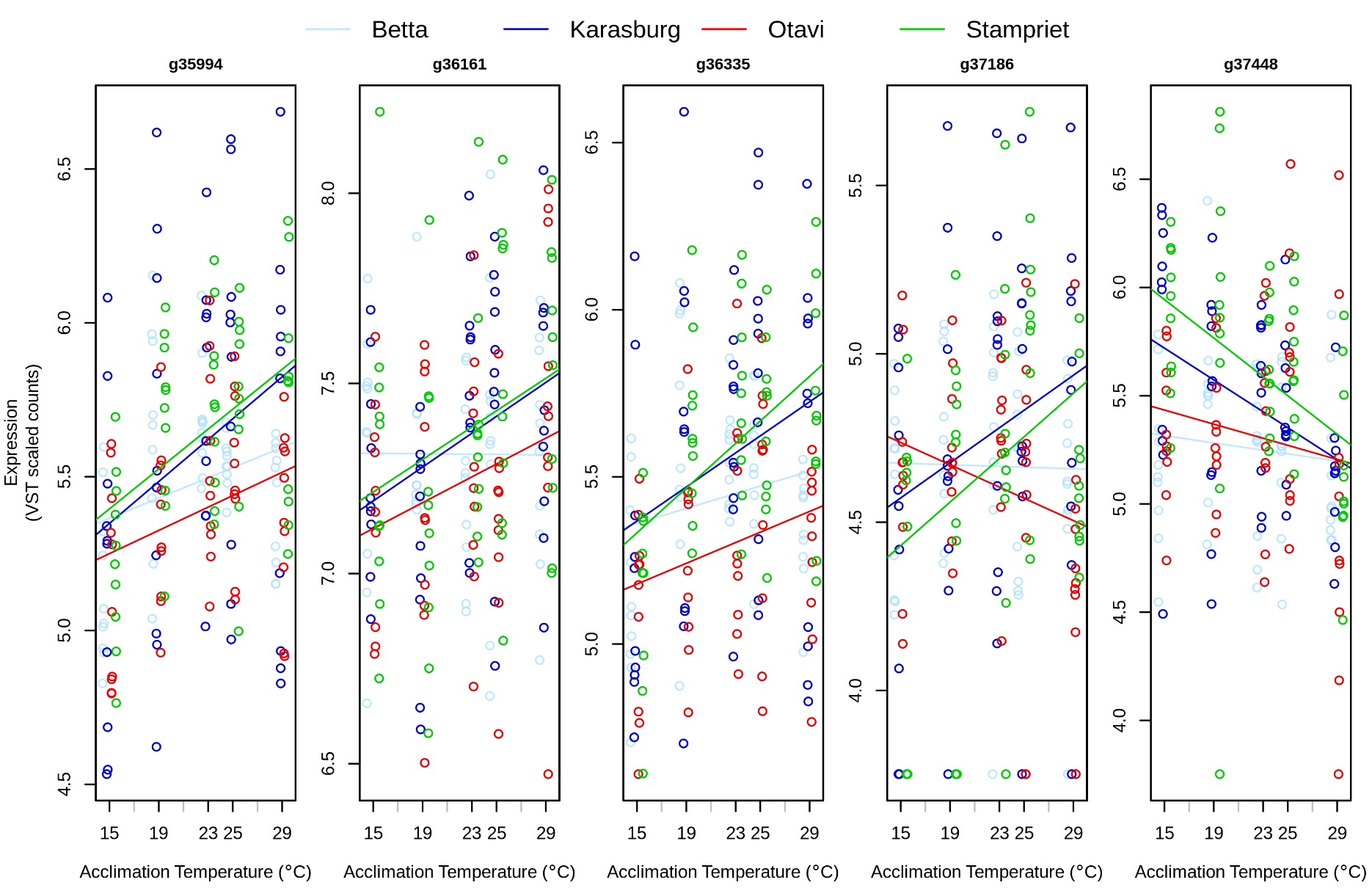


## Figure S25: Hydrophilic metabolites with similar population specific acclimation responses as the heat tolerance phenotype

Metabolites with intensity levels showing similar population-specific trends as that of Heat tolerance (CTmax). These 4 metabolites were identified using NMR on spiders in aquatic solution. Metabolites presented in these graphs show a response suggesting an involvement in the observed population dependent heat tolerance, thus linking metabolites to the expressed phenotype.


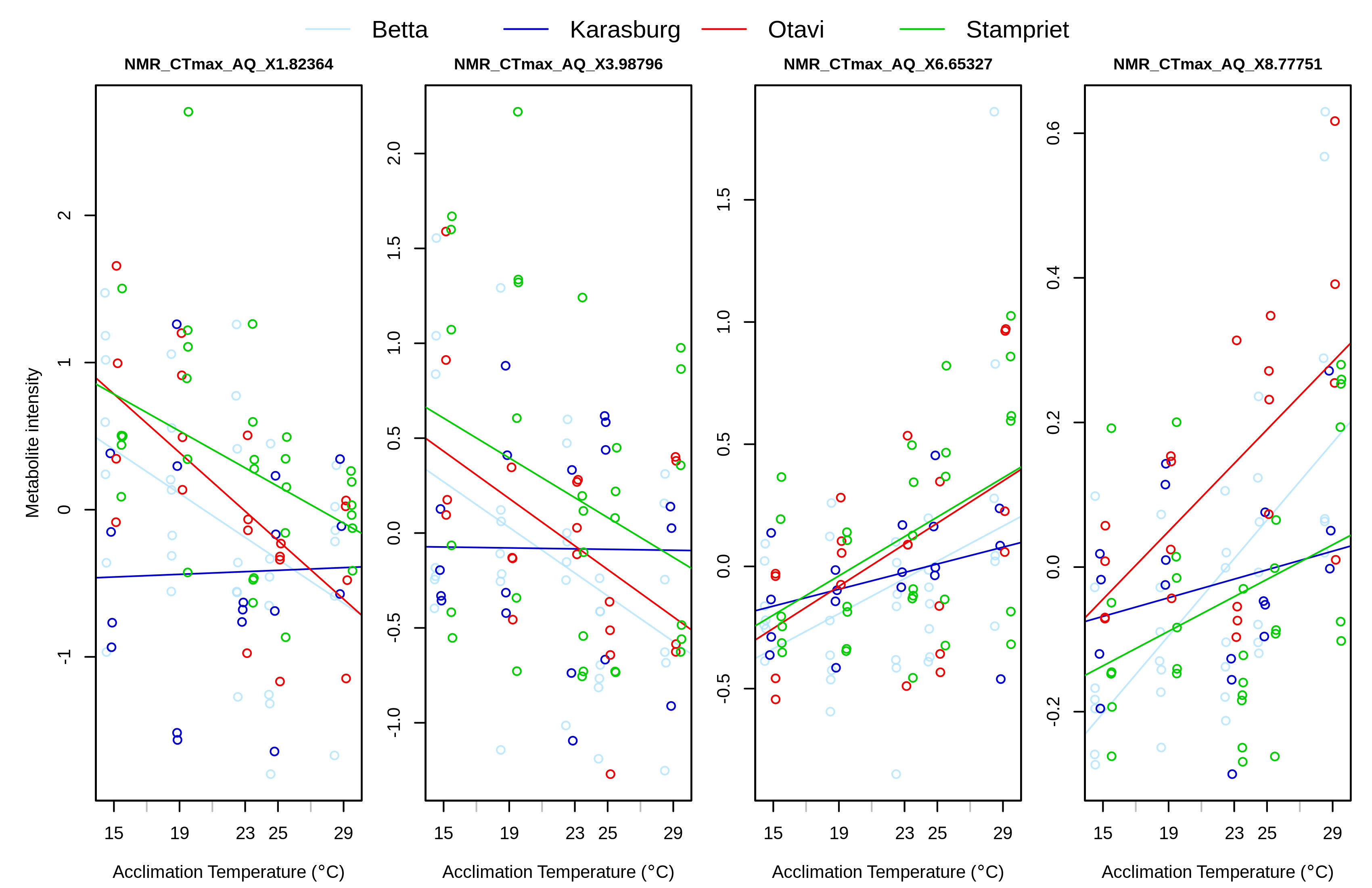


## Figure S26: Hydrophobic metabolites with similar population specific acclimation responses as the heat tolerance phenotype

Metabolites with intensity levels showing similar population-specific trends as that of Heat tolerance (CTmax). These 2 metabolites were identified using NMR on spiders in organic solution. Metabolites presented in these graphs show a response suggesting an involvement in the observed population dependent heat tolerance, thus linking metabolites to the expressed phenotype.


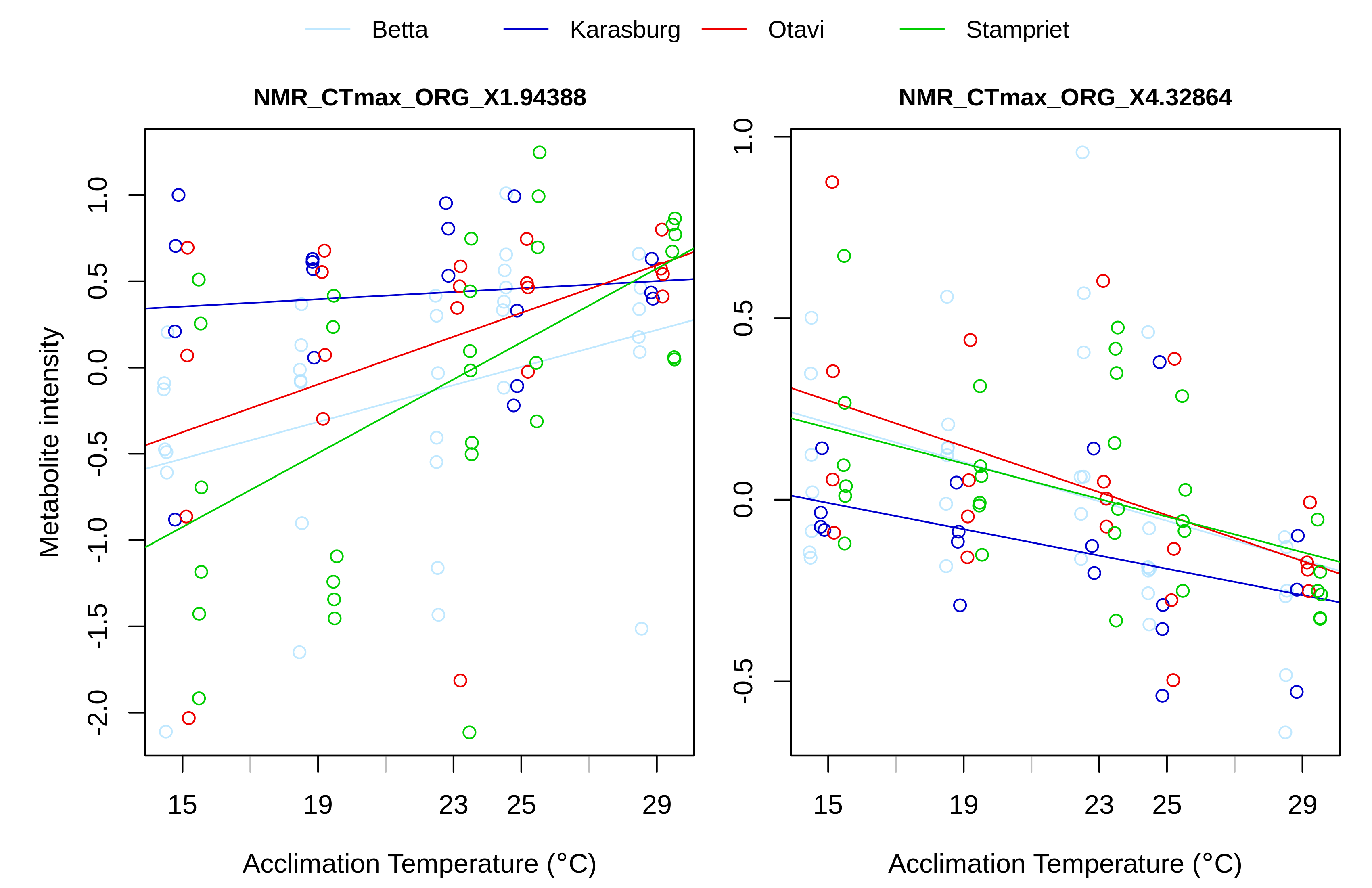


## Figure S27: Hydrophilic metabolites with similar population specific acclimation responses as the cold tolerance phenotype

Metabolites with intensity levels showing similar population-specific trends as that of Cold tolerance (CCRTemp). This metabolite was identified using NMR on spiders in aquatic solution. The metabolite presented in here show a response suggesting an involvement in the observed population dependent cold tolerance, thus potentially linking metabolite to the expressed phenotype.


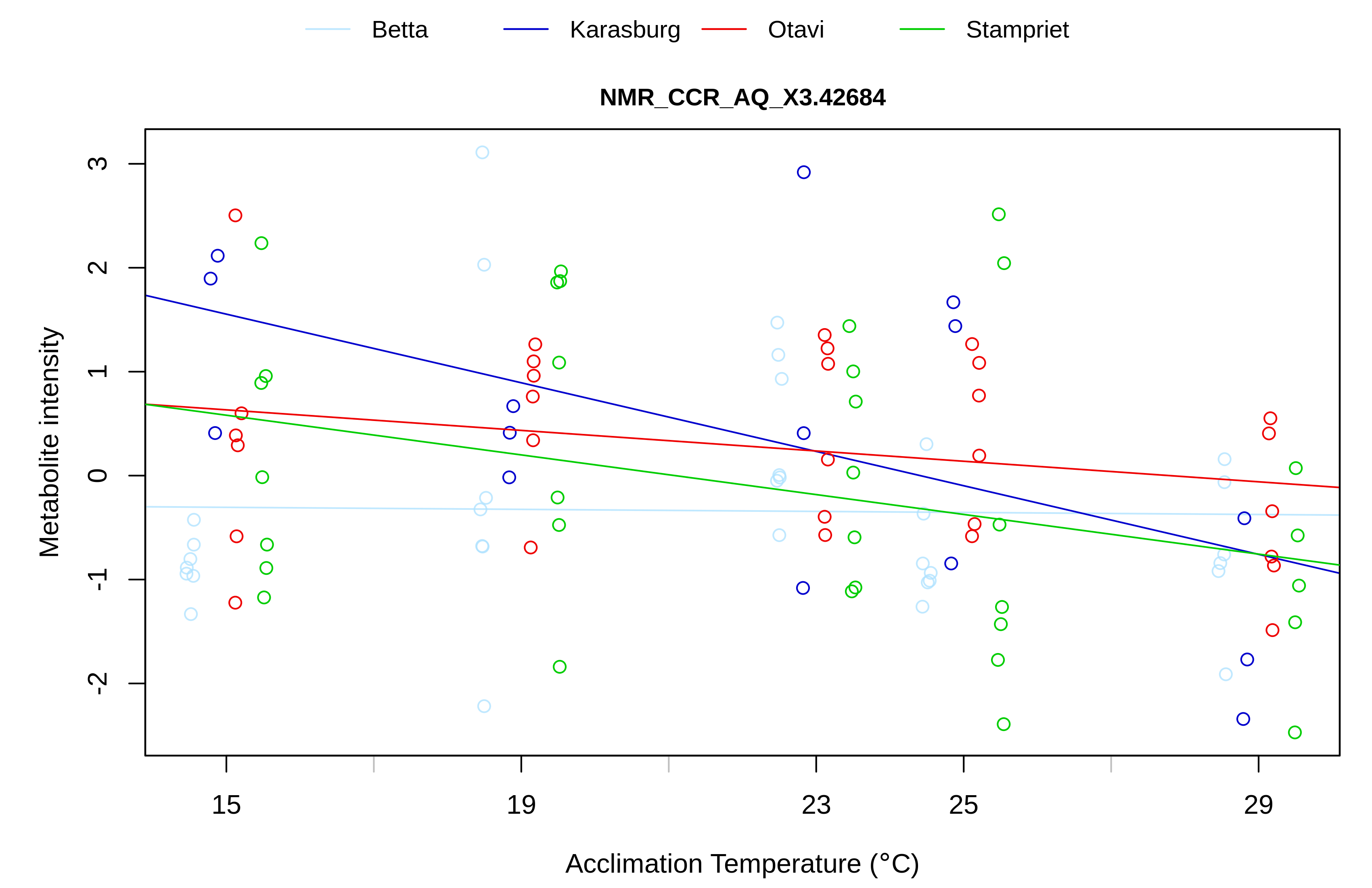


## Figure S28: Phylogenies with scenarios for gain/loss of plasticity in heat and cold tolerance

Phylogenetic relationships showing the possible scenarios for ancestral loss or gain of plasticity for temperature tolerances in *S. dumicola* spiders.


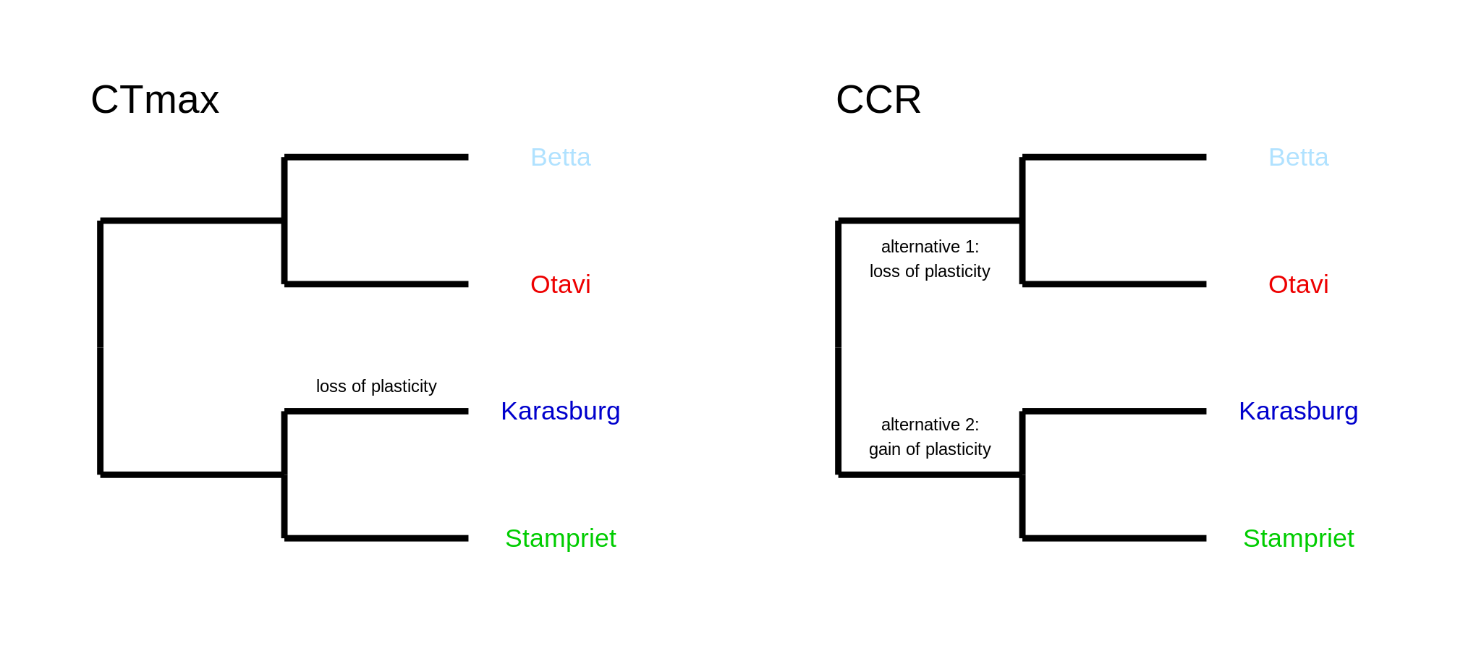


## Figure S29: PCA of microbiomic data

Principal component analyses of microbiome ASVs with population effects. PCA analysis on differential microbiome ASV abundances with population effect. Three principal components are plotted, along with a screeplot and a biplot. Karasburg (blue) separates clearly from Otavi (red), Betta (light blue) and Stampriet (green).


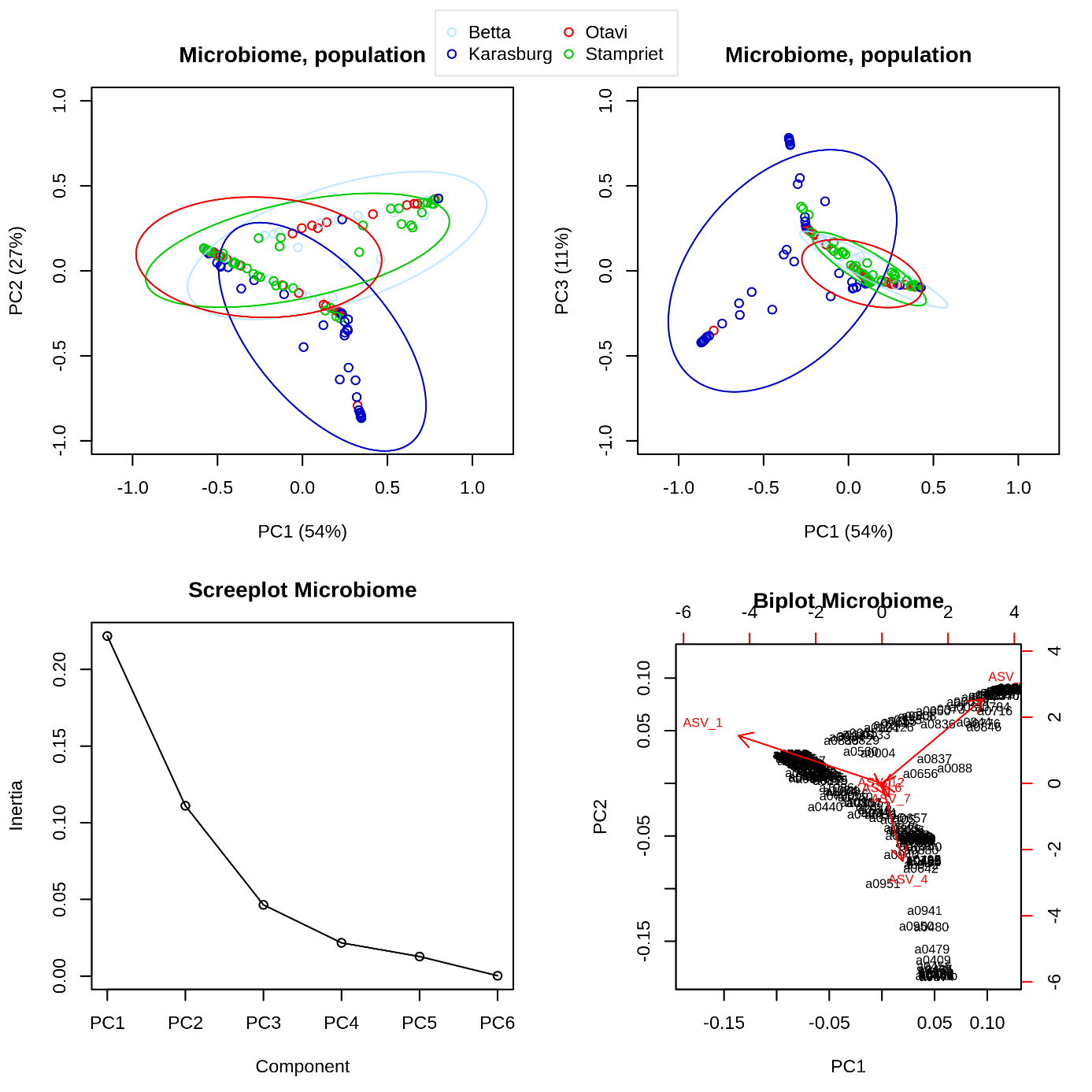


## Figure S30: Plots of microbiomic ASVs with population effect

Reaction norms for microbiome ASV abundances for two acclimation temperatures. The graphs show relative abundance as a function of temperature acclimation for each of the ASVs showing population effects.


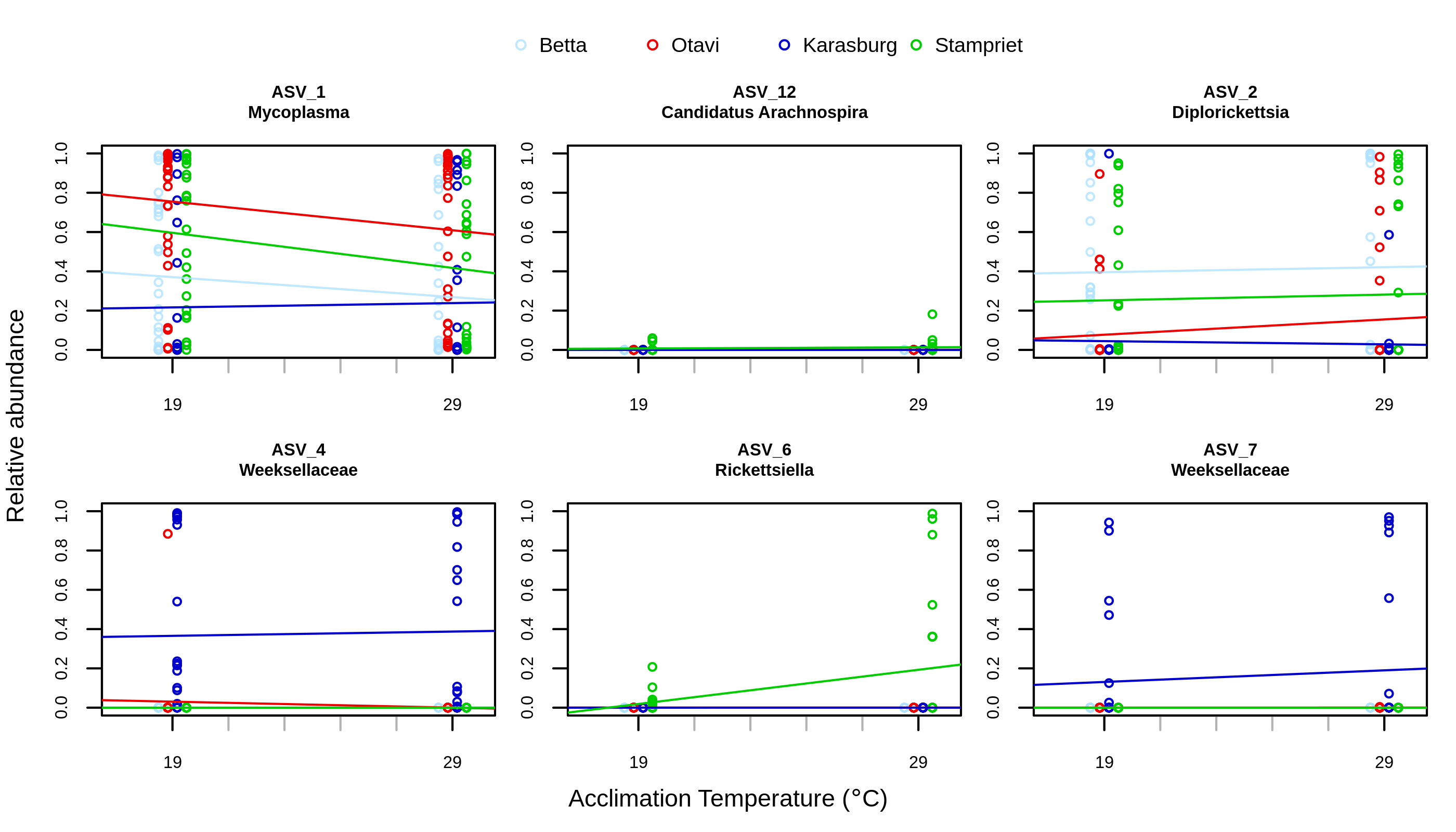


ASV_1 in this study corresponds to ASV_4 in Busck et al., (2020).

ASV_2 in this study corresponds to ASV_9 in Busck et al., (2020).

ASV_12 *Candidatus* Arachnospira was formerly classified as *Borrelia.*

## Figure S31: Coverage plot for methylation data

Plot of the coverage distribution for the methylation data. The maximum coverage threshold of 32x were determined by estimating the top one percent of the distribution, and confirmed via visual inspection.


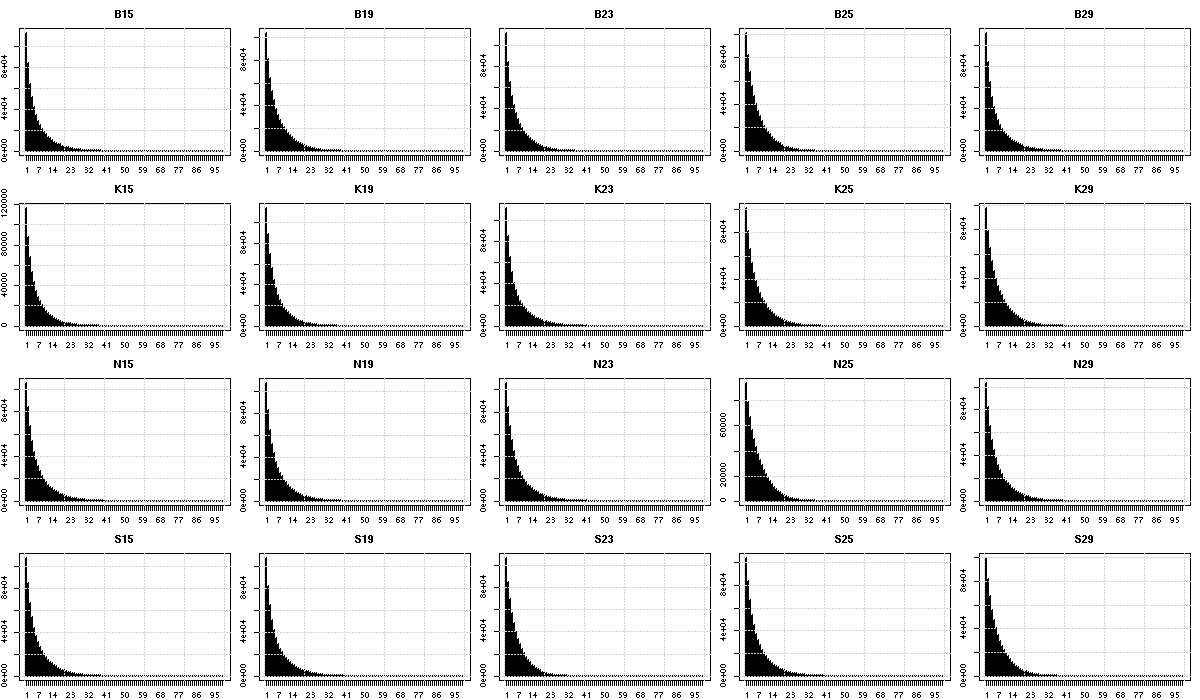


## Figure S32: PCA of metabolite NMR-Org data from the CTMax and CCRTemp treatment

Principal Component Analysis of Metabolites from NMR from spiders having gone through CTmax (a, c) or CCR (b, d) treatment. Only metabolites that showed temperature effect are plotted. No metabolites extracted using organic solution showed population responses, thus there is no graph colored by population. Here the first three principal components are plottet.

**
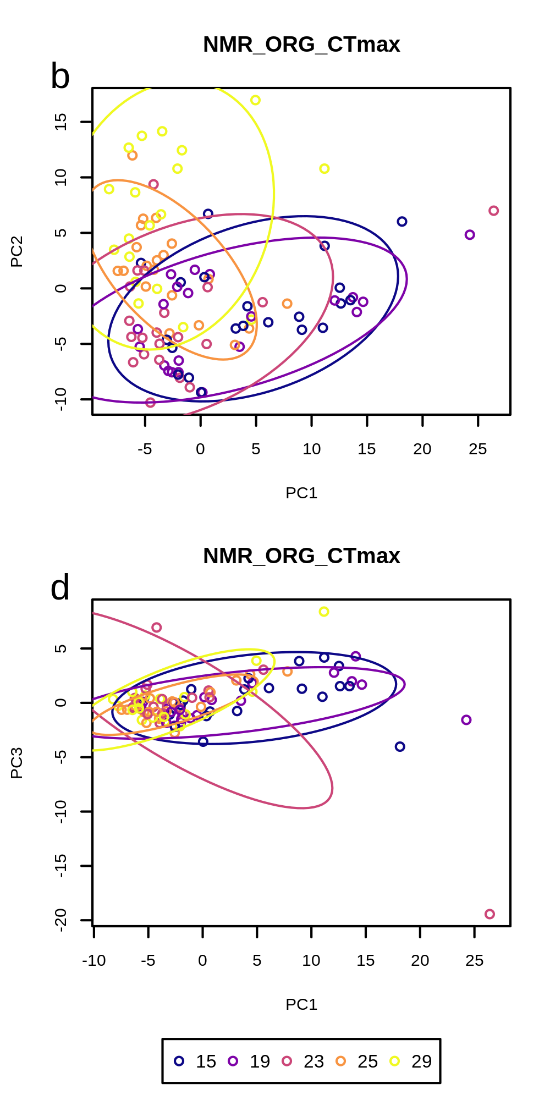

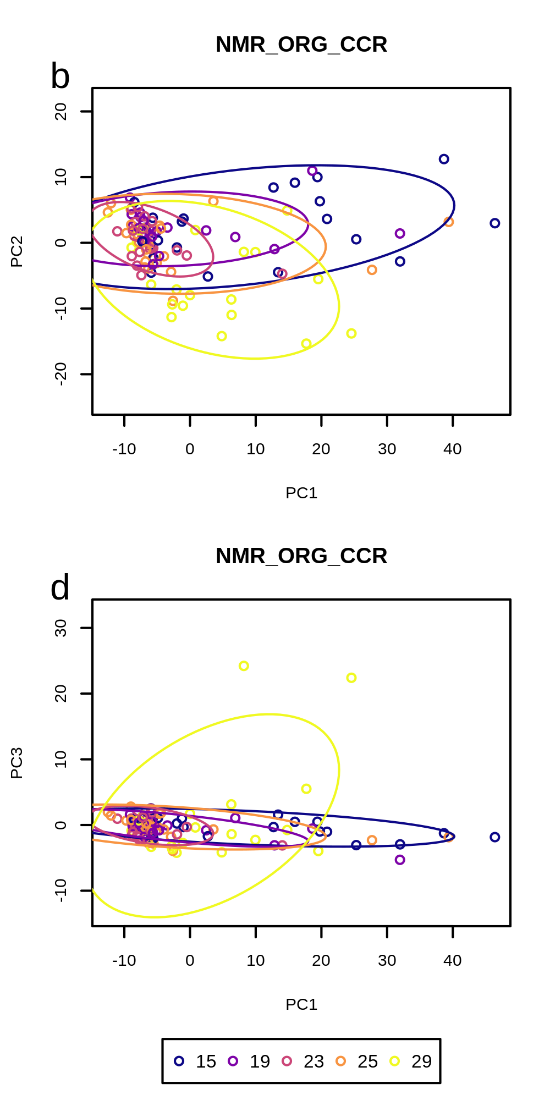
**

a

c

## Figure S33: Methylation in plastically expressed genes in response to temperature acclimation

Violin plots of weighted methylation level in three categories of genes: genes that are not plastically expressed in our study, genes that are plastically expressed, or plastically expressed genes with similar population patterns as temperature tolerance phenotypes. Plastic genes show a highly bimodal pattern (middle), which means that a subset of plastic genes have low methylation level, whereas other genes are plastic despite the potential inhibition from methylation, and may thus be regulated by other means than methylation. Plastic genes more likely to be involved in temperature tolerances (right) are generally highly methylated.


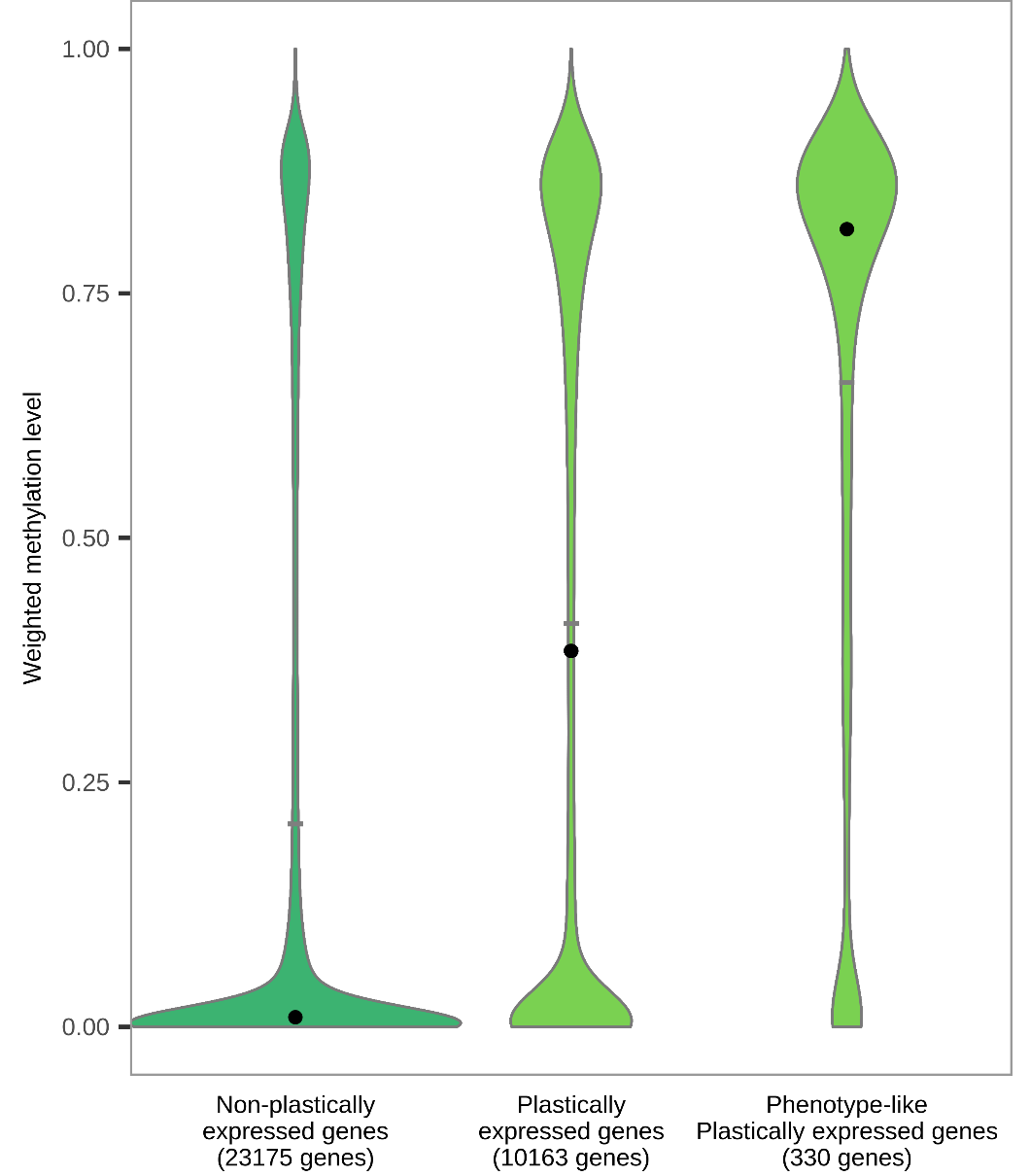


## Table S1: Number of social spider *S. dumicola* nest replicates per population/acclimation group

|  | **Count of Nests/boxes** |
| --- | --- |
| **Betta** | **101** |
| 15 | 20 |
| 19 | 20 |
| 23 | 20 |
| 25 | 20 |
| 29 | 21 |
| **Karasburg** | **65** |
| 15 | 13 |
| 19 | 13 |
| 23 | 13 |
| 25 | 13 |
| 29 | 13 |
| **Otavi** | **111** |
| 15 | 22 |
| 19 | 22 |
| 23 | 22 |
| 25 | 22 |
| 29 | 23 |
| **Stampriet** | **94** |
| 15 | 19 |
| 19 | 18 |
| 23 | 18 |
| 25 | 19 |
| 29 | 20 |
| **Grand Total** | **371** |

## Table S2: Table of LCMS metabolites and their effects

Metabolites identified by Liquid Chromatography coupled Mass Spectrometer (LC-MS) that were significantly different between treatments or populations. Suffix “_neg” means feature retrieved using negative ionization; Suffix “_pos” means feature retrieved using positive ionization.

| **Feature ID** | **Metabolite** | **Group** | **Assay** | **Significance** | **ID Level^*^** |
| --- | --- | --- | --- | --- | --- |
| M229T45_neg | D-Ribulose 5-phosphate | sugar | CCR | interaction | 2 |
| M250T42_pos | 2'-Deoxyadenosine | nucleoside precursor/ degradation product | CCR | interaction | 2 |
| M88T39_neg | L-Alanine | amino acid | CCR | interaction | 1 |
| M494T646_pos | L-α-Lysophosphatidyl-choline; 16:0 | lipid derivative | CCR | interaction | 1 |
| M145T39_neg | L-glutamine | amino acid | CCR | plastic | 1 |
| M180T105_neg | Tyrosine | amino acid | CCR | plastic | 1 |
| M258T41_neg | D-Glucosamine 6-phosphate | hexose phosphate | CCR | plastic | 2 |
| M243T133_neg | Uridine | nucleoside | CCR | plastic | 1 |
| M110T41_2_pos | Cytosine | nucleotide | CCR | plastic | 2 |
| M229T45_neg | D-Ribulose 5-phosphate | sugar | CCR | plastic | 2 |
| M339T37_neg | Fructose 1,6-bisphosphate | hexose phosphate | CCR | plastic | 2 |
| M145T253_neg | 2-Methylglutaric acid | TCA degradation product | CCR | plastic | 2 |
| M383T176_neg | 5-Adenosyl-L-homocysteine | amino acid derivative, metabolic intermediate | CCR | plastic | 2 |
| M89T71_neg | D-Lactate | fermentation product | CCR | plastic | 1 |
| M195T35_pos | Gluconate | sugar derivative | CCR | plastic | 2 |
| M306T80_neg | Glutathione reduced | tripeptide, antioxidant | CCR | plastic | 1 |
| M174T41_neg | L-Citrulline | amino acid | CCR | plastic | 2 |
| M203T264_neg | L-Tryptophane | amino acid | CCR | plastic | 1 |
| M664T67_neg | NADH | redox carrier | CCR | plastic | 2 |
| M122T103_neg | Nicotinic acid (niacin) | acid, vitamin | CCR | plastic | 2 |
| M288T106_neg | Ophthalmic acid | oligopeptide | CCR | plastic | 2 |
| M74T42_pos | Trimethylamine N-oxide | amine oxide | CCR | plastic | 2 |
| M151T114_neg | Xanthine | nucleoside precursor/ degradation product | CCR | plastic | 1 |
| M283T230_neg | Xanthosine | nucleoside | CCR | plastic | 1 |
| M204T285_neg | Xanthurenic acid | carboxylic acid | CCR | plastic | 1 |
| M74T38_neg | Glycine | amino acid | CCR | population | 1 |
| M131T38_neg | L-Asparagine | amino acid | CCR | population | 1 |
| M130T119_neg | L-Leucine | amino acid | CCR | population | 1 |
| M164T229_neg | Phenylalanine | amino acid | CCR | population | 2 |
| M180T105_neg | Tyrosine | amino acid | CCR | population | 2 |
| M157T43_2_neg | Allantoin | metabolic intermediate | CCR | population | 2 |
| M133T56_neg | Malic acid | metabolic intermediate | CCR | population | 1 |
| M110T41_2_pos | cytosine | nucleotide | CCR | population | 2 |
| M171T43_2_neg | Glycerol 3-phosphate | polyol | CCR | population | 1 |
| M229T45_neg | D-Ribulose 5-Phosphate | sugar | CCR | population | 2 |
| M339T37_neg | Fructose 1,6-bisphosphate | hexose phosphate | CCR | population | 2 |
| M259T42_neg | Fructose 6-phosphate | hexose phosphate | CCR | population | 2 |
| M153T287_neg | 2,3-Dihydroxybenzoic acid | phenolic compound | CCR | population | 2 |
| M250T42_pos | 2'-Deoxyadenosine | nucleoside precursor/ degradation product | CCR | population | 2 |
| M266T219_neg | 2'-Deoxyguanosine | nucleoside | CCR | population | 2 |
| M267T217_neg | 2-Deoxyxanthosine | nucleoside | CCR | population | 2 |
| M145T253_neg | 2-Methylglutaric acid | TCA degradation product | CCR | population | 2 |
| M275T47_neg | 6-Phosphogluconic acid | pentose phosphate pathway metabolic intermediate | CCR | population | 2 |
| M105T47_neg | D-Glyceric Acid | organic acid | CCR | population | 1 |
| M195T35_pos | Gluconate | sugar derivative | CCR | population | 2 |
| M150T63_neg | Guanine | nucleotide | CCR | population | 1 |
| M108T41_pos | Hypotaurine | osmolyte, antioxidant | CCR | population | 2 |
| M190T290_neg | N-acetyl-DL-methionine | amino acid derivative | CCR | population | 1 |
| M662T146_neg | NAD+ | redox carrier | CCR | population | 2 |
| M382T289_2_pos | Succinyladenosine | nucleoside derivative | CCR | population | 1 |
| M283T230_neg | Xanthosine | nucleoside | CCR | population | 1 |
|  |  |  |  |  |  |
| M190T290_neg | N-acetyl-DL-methionine | amino acid derivative | Ctmax | interaction | 1 |
| M383T176_neg | 5-adenosyl-L-homocysteine | amino acid derivative, metabolic intermediate | Ctmax | interaction | 2 |
| M136T105_neg | Tyramine | amino acid derivative | Ctmax | interaction | 2 |
| M130T42_pos | Hydroxyproline | amino acid | Ctmax | interaction | 2 |
| M160T45_neg | L-2-aminoadipic acid | metabolic intermediate | Ctmax | interaction | 2 |
| M173T36_neg | L-Arginine | amino acid | Ctmax | interaction | 2 |
| M131T38_neg | L-Asparagine | amino acid | Ctmax | interaction | 1 |
| M145T39_neg | L-Glutamine | amino acid | Ctmax | interaction | 1 |
| M131T36_neg | L-Ornithine | amino acid | Ctmax | interaction | 2 |
| M118T39_neg | L-Threonine | amino acid | Ctmax | interaction | 2 |
| M180T38_pos | Tyrosine | amino acid | Ctmax | interaction | 1 |
| M258T41_neg | D-Glucosamine 6-phosphate | hexose phosphate | Ctmax | interaction | 2 |
| M220T38_pos | N-acetyl-D-glucosamine | amino sugar | Ctmax | interaction | 2 |
| M137T293_neg | 4-Hydroxybenzoic acid | aromatic acid | Ctmax | interaction | 2 |
| M263T286_neg | Phe-Val | dipeptide | Ctmax | interaction | 2 |
| M89T71_neg | D-Lactate | fermentation product | Ctmax | interaction | 1 |
| M157T43_2_neg | Allantoin | metabolic intermediate | Ctmax | interaction | 2 |
| M115T128_neg | Fumaric acid | TCA cycle intermediate | Ctmax | interaction | 1 |
| M117T111_neg | Succinic acid | TCA cycle intermediate | Ctmax | interaction | 1 |
| M122T103_neg | Nicotinic acid (niacin) | acid, vitamin | Ctmax | interaction | 2 |
| M250T42_pos | 2'-Deoxyadenosine | nucleoside precursor/ degradation product | Ctmax | interaction | 2 |
| M242T59_neg | Cytidine | nucleoside | Ctmax | interaction | 2 |
| M150T63_neg | Guanine | nucleoside | Ctmax | interaction | 1 |
| M243T133_neg | Uridine | nucleoside | Ctmax | interaction | 1 |
| M135T43_2_neg | Hypoxanthine | nucleoside precursor/ degradation product | Ctmax | interaction | 2 |
| M347T101_neg | Inosine 5'-monophosphate | nucleoside precursor/ degradation product | Ctmax | interaction | 2 |
| M363T118_neg | Xanthosine 5' monophosphate | nucleoside precursor/ degradation product | Ctmax | interaction | 2 |
| M275T47_neg | 6-Phosphogluconic acid | pentose phosphate pathway metabolic intermediate | Ctmax | interaction | 2 |
| M131T216_neg | Glutaric acid | carboxylic acid | Ctmax | interaction | 2 |
| M108T41_pos | Hypotaurine | osmolyte, antioxidant | Ctmax | interaction | 2 |
| M124T33_pos M124T38_1_neg | Taurine | osmolyte | Ctmax | interaction | 1 |
| M455T327_neg | Flavin mononucleotide (FMN) | redox carrier | Ctmax | interaction | 1 |
| M147T79_neg | L-2-Hydroxyglutaric acid | short chain hydroxy acid | Ctmax | interaction | 2 |
| M199T46_neg | D-Erythrose 4-phosphate | pentose phosphate pathway intermediate | Ctmax | interaction | 2 |
| M339T37_neg | Fructose 1,6-bisphosphate | hexose phosphate | Ctmax | interaction | 2 |
| M133T56_neg | Malic acid | TCA cycle intermediate | Ctmax | interaction | 2 |
| M145T253_neg | 2-Methylglutaric acid | TCA degradation product | Ctmax | interaction | 2 |
| M611T238_neg | Glutathione oxidized | tripeptide | Ctmax | interaction | 1 |
| M218T258_neg | Pantothenic acid | vitamin B5 | Ctmax | interaction | 1 |
| M188T114_neg | N-Acetyl-L-glutamic acid | metabolic intermediate | Ctmax | plastic | 2 |
| M113T44_pos | 3-amino-2-piperidone | amino acid derivative | Ctmax | plastic | 2 |
| M204T285_neg | Xanthurenic acid | carboxylic acid | Ctmax | plastic | 1 |
| M188T297_pos | Kynurenic acid | amino acid derivative | Ctmax | plastic | 2 |
| M160T45_neg | L-2-Aminoadipic acid | metabolic intermediate | Ctmax | plastic | 2 |
| M173T36_neg | L-Arginine | amino acid | Ctmax | plastic | 2 |
| M174T41_neg | L-Citrulline | amino acid | Ctmax | plastic | 2 |
| M130T119_neg | L-Leucine | amino acid | Ctmax | plastic | 1 |
| M203T264_neg | L-Tryptophane | amino acid | Ctmax | plastic | 2 |
| M164T229_neg | Phenylalanine | amino acid | Ctmax | plastic | 2 |
| M180T105_neg M180T38_pos | Tyrosine | amino acid | Ctmax | plastic | 1 |
| M258T41_neg | D-Glucosamine 6-phosphate | hexose phosphate | Ctmax | plastic | 2 |
| M220T38_pos | N-acetyl-D-glucosamine | amino sugar | Ctmax | plastic | 2 |
| M160T262_neg | Indole-3-carboxylic acid | amino acid derivative | Ctmax | plastic | 2 |
| M263T291_pos | Phe-Val | dipeptide | Ctmax | plastic | 2 |
| M494T646_pos | L-α-Lysophosphatidyl-choline; 16:0 | lipid derivative | Ctmax | plastic | 1 |
| M191T82_neg | Citric acid | TCA cycle intermediate | Ctmax | plastic | 1 |
| M116T48_pos | N,N-dimethylalanine | amino acid derivative | Ctmax | plastic | 2 |
| M122T103_neg | nicotinic acid (niacin) | acid, vitamin | Ctmax | plastic | 2 |
| M242T59_neg | Cytidine | nucleoside | Ctmax | plastic | 2 |
| M243T133_neg | Uridine | nucleoside | Ctmax | plastic | 1 |
| M135T43_2_neg | Hypoxanthine | nucleoside precursor/ degradation product | Ctmax | plastic | 2 |
| M347T101_neg | Inosine 5'-monophosphate | nucleoside precursor/ degradation product | Ctmax | plastic | 2 |
| M151T114_neg | Xanthine | nucleoside precursor/ degradation product | Ctmax | plastic | 1 |
| M283T230_neg | Xanthosine | nucleoside | Ctmax | plastic | 1 |
| M322T53_neg | Cytidine 5-monophosphate | nucleotide | Ctmax | plastic | 2 |
| M282T218_neg | Guanosine | nucleotide | Ctmax | plastic | 2 |
| M323T72_neg | Uridine 5-monophosphate | nucleotide | Ctmax | plastic | 1 |
| M275T47_neg | 6-Phosphogluconic acid | pentose phosphate pathway metabolic intermediate | Ctmax | plastic | 2 |
| M195T42_2_neg | Gluconate | sugar derivative | Ctmax | plastic | 2 |
| M87T82_neg | Pyruvic acid | organic acid | Ctmax | plastic | 2 |
| M116T39_pos | Betaine | osmolyte | Ctmax | plastic | 2 |
| M108T41_pos | Hypotaurine | osmolyte, antioxidant | Ctmax | plastic | 2 |
| M124T33_pos M124T38_1_neg | Taurine | osmolyte | Ctmax | plastic | 1 |
| M171T43_2_neg | Glycerol 3-phosphate | polyol | Ctmax | plastic | 1 |
| M199T46_neg | D-Erythrose 4-phosphate | pentose phosphate pathway intermediate | Ctmax | plastic | 2 |
| M339T37_neg | Fructose 1,6-bisphosphate | hexose phosphate | Ctmax | plastic | 2 |
| M259T42_neg | Fructose 6-phosphate | hexose phosphate | Ctmax | plastic | 2 |
| M133T56_neg | Malic acid | TCA cycle intermediate | Ctmax | plastic | 2 |
| M306T80_neg | Glutathione reduced | tripeptide, antioxidant | Ctmax | plastic | 1 |
| M288T106_neg | Ophthalmic acid | oligopeptide | Ctmax | plastic | 2 |
| M375T333_neg | Riboflavin | vitamin B2 | Ctmax | plastic | 1 |
| M245T46_2_neg | 1-(3-sn-phosphatidyl)-rac-glycerol | lipid derivative | Ctmax | plastic | 2 |
| M136T105_neg | Tyramine | amino acid derivative | Ctmax | population | 2 |
| M188T297_pos | Kynurenic acid | amino acid derivative | Ctmax | population | 2 |
| M74T42_pos | Trimethylamine N-oxide | amine oxide | Ctmax | population | 2 |
| M74T44_pos | Glycine | amino acid | Ctmax | population | 1 |
| M173T36_neg | L-Arginine | amino acid | Ctmax | population | 2 |
| M131T38_neg | L-Asparagine | amino acid | Ctmax | population | 1 |
| M132T39_neg | L-Aspartic acid | amino acid | Ctmax | population | 2 |
| M130T119_neg | L-Leucine | amino acid | Ctmax | population | 1 |
| M164T229_neg | Phenylalanine | amino acid | Ctmax | population | 2 |
| M258T41_neg | D-Glucosamine 6-phosphate | hexose phosphate | Ctmax | population | 2 |
| M137T293_neg | 4-Hydroxybenzoic acid | aromatic acid | Ctmax | population | 2 |
| M263T286_neg | Phe-Val | dipeptide | Ctmax | population | 2 |
| M157T43_2_neg | Allantoin | metabolic intermediate | Ctmax | population | 2 |
| M115T128_neg | Fumaric acid | TCA cylce intermediate | Ctmax | population | 1 |
| M122T103_neg | nicotinic acid (niacin) | acid, vitamin | Ctmax | population | 2 |
| M250T42_pos | 2'-Deoxyadenosine | nucleoside precursor/ degradation product | Ctmax | population | 2 |
| M267T217_neg | 2-Deoxyxanthosine | nucleoside | Ctmax | population | 2 |
| M135T43_2_neg | Hypoxanthine | nucleoside precursor/ degradation product | Ctmax | population | 2 |
| M347T101_neg | Inosine 5'-monophosphate | nucleoside precursor/ degradation product | Ctmax | population | 2 |
| M151T114_neg | Xanthine | nucleoside precursor/ degradation product | Ctmax | population | 1 |
| M282T218_neg | Guanosine | nucleotide | Ctmax | population | 2 |
| M105T47_neg | D-Glyceric Acid | organic acid | Ctmax | population | 1 |
| M131T216_neg | Glutaric acid | carboxylic acid | Ctmax | population | 2 |
| M108T41_pos | Hypotaurine | osmolyte, antioxidant | Ctmax | population | 2 |
| M124T38_1_neg | Taurine | osmolyte | Ctmax | population | 1 |
| M171T43_2_neg | Glycerol 3-phosphate | polyol | Ctmax | population | 1 |
| M664T67_neg | NADH | redox carrier | Ctmax | population | 2 |
| M147T79_neg | L-2-Hydroxyglutaric acid | short chain hydroxy acid | Ctmax | population | 2 |
| M199T46_neg | D-Erythrose 4-phosphate | pentose phosphate pathway intermediate | Ctmax | population | 2 |
| M145T253_neg | 2-Methylglutaric acid | TCA degradation product | Ctmax | population | 2 |
| M166T45_pos | Pyridoxal | vitamin B6 | Ctmax | population | 2 |
| M245T46_2_neg | 1-(3-sn-phosphatidyl)-rac-glycerol | lipid derivative | Ctmax | population | 2 |

* According to Sumner et al., (2007): 1 = verified by authentic standard, 2 = putatively annotated compounds verified by MS spectral libraries.

## Table S3: Model summary output for growth rate model

Best model summary output for growth rate data of *S. dumicola* spiders. The analysis was based on grand mean centered treatment data (acclimation temperature).

Linear model: growth rate ~ Population * Treatment

|  | **Growth rate** | | | | |
| --- | --- | --- | --- | --- | --- |
| *Predictors* | *Estimates* | *std. Error* | *CI* | *Statistic* | *p* |
| (Intercept) | 1.53 ^***^ | 0.05 | 1.43 – 1.62 | 31.10 | **<0.001** |
| Population [Karasburg] | -0.05 | 0.08 | -0.21 – 0.11 | -0.64 | 0.525 |
| Population [Otavi] | -0.11 | 0.07 | -0.25 – 0.02 | -1.62 | 0.106 |
| Population [Stampriet] | -0.07 | 0.09 | -0.25 – 0.11 | -0.76 | 0.447 |
| Treatment | 0.10 ^***^ | 0.01 | 0.08 – 0.12 | 10.29 | **<0.001** |
| Population [Karasburg] * Treatment | 0.01 | 0.02 | -0.03 – 0.04 | 0.30 | 0.762 |
| Population [Otavi] * Treatment | -0.03 ^*^ | 0.01 | -0.06 – -0.00 | -2.01 | **0.045** |
| Population [Stampriet] * Treatment | 0.00 | 0.02 | -0.03 – 0.04 | 0.23 | 0.817 |
| Observations | 304 | | | | |
| R^2^ / R^2^ adjusted | 0.490 / 0.478 | | | | |
| AIC | 439.668 | | | | |
| F-statistic | 40.66 on 7 and 296 DF | | | | |
| p-value | < 2.2e-16 | | | | |
| ** p < 0.05   ** p < 0.01   *** p < 0.001* | | | | | |

## Table S4: ANOVA output for model on growth rate

ANOVA output from the best model on growth rate of *S. dumicola* spiders. The analysis was based on grand mean centered treatment data (acclimation temperature).

Linear model: Growth rate ~ Population * Treatment

| **ANOVA on Growth rate model** | | | | | | |
| --- | --- | --- | --- | --- | --- | --- |
| *Row* | *Df* | *Sum Sq* | *Mean Sq* | *F value* | *Pr(>F)* |  |
| Population | 3 | 0.84 | 0.28 | 1.16 | 0.33 |  |
| Treatment | 1 | 66.11 | 66.11 | 274.65 | 0.00 | *** |
| Population:Treatment | 3 | 1.56 | 0.52 | 2.16 | 0.09 |  |
| Residuals | 296 | 71.25 | 0.24 |  |  |  |

** p < 0.05   ** p < 0.01   *** p < 0.001*

## Table S5: ANOVA output for model on survival

Anova() output from the best model on survival of *S. dumicola* spiders compared to the null model. The analysis was based on grand mean centered treatment data (acclimation temperature).

General linear model, binomial, logit link: Survival ~ Population + Treatment.

| **Analysis of deviance on Survival model** | | | | | | |
| --- | --- | --- | --- | --- | --- | --- |
| *Row* | *Df* | *Deviance* | *Resid. Df* | *Resid. Dev* | *Pr(>Chi)* |  |
| Null |  |  | 303 | 1325.39 |  |  |
| Population | 3 | 17.99 | 300 | 1307.39 | 0.00 | *** |
| Treatment | 1 | 233.25 | 299 | 1074.15 | 0.00 | *** |

** p < 0.05   ** p < 0.01   *** p < 0.001*

## Table S6: Model summary for survival model

Best model summary output for survival data of *S. dumicola* spiders. The analysis was based on grand mean centered treatment data (acclimation temperature).

General linear model, binomial, logit link: Survival ~ Population + Treatment.

|  | **Survival data** | | | | |
| --- | --- | --- | --- | --- | --- |
| *Predictors* | *Log-Odds* | *std. Error* | *CI* | *Statistic* | *p* |
| (Intercept) | 2.11 ^***^ | 0.06 | 1.99 – 2.22 | 35.75 | **<0.001** |
| Population [Karasburg] | 0.42 ^***^ | 0.10 | 0.22 – 0.63 | 4.02 | **<0.001** |
| Population [Otavi] | 0.06 | 0.08 | -0.10 – 0.22 | 0.73 | 0.466 |
| Population [Stampriet] | 0.12 | 0.11 | -0.09 – 0.33 | 1.10 | 0.274 |
| Treatment | 0.11 ^***^ | 0.01 | 0.09 – 0.12 | 14.82 | **<0.001** |
| Observations | 304 | | | | |
| R^2^ Tjur | 0.010 | | | | |
| R^2^ McFadden (adj) | 0.125 | | | | |
| AIC | 1754.826 | | | | |
| p-value (to null model) | < 2.2e-16 | | | | |
| ** p < 0.05   ** p < 0.01   *** p < 0.001* | | | | | |

## Table S7: Test output on whether CTmax population trends differ from zero

Table of the summary from the emtrends() function, testing whether the population trends are significantly different from zero. The linear model tested were based on CTmax temperature tolerance data.

| **CTmax - Trendlines diverging from zero** | | | | | | | |
| --- | --- | --- | --- | --- | --- | --- | --- |
| *Population* | *Treatment.trend* | *SE* | *df* | *lower.CL* | *upper.CL* | *t.ratio* | *p.value* |
| Betta | 0.104 | 0.010 | 298 | 0.084 | 0.124 | 10.057 | 0.000 |
| Karasburg | 0.022 | 0.013 | 298 | -0.004 | 0.048 | 1.690 | 0.092 |
| Otavi | 0.086 | 0.011 | 298 | 0.064 | 0.108 | 7.674 | 0.000 |
| Stampriet | 0.042 | 0.013 | 298 | 0.017 | 0.066 | 3.319 | 0.001 |

## Table S8: Test output on whether CCRTemp population trends differ from zero

Table of the summary from the emtrends() function, testing whether the population trends are significantly different from zero. The linear model tested were based on CCRTemp temperature tolerance data.

| **CCRTemp - Trendlines diverging from zero** | | | | | | | |
| --- | --- | --- | --- | --- | --- | --- | --- |
| *Population* | *Treatment.trend* | *SE* | *df* | *lower.CL* | *upper.CL* | *t.ratio* | *p.value* |
| Betta | 0.028 | 0.047 | 320 | -0.065 | 0.121 | 0.589 | 0.557 |
| Karasburg | 0.201 | 0.059 | 320 | 0.085 | 0.316 | 3.408 | 0.001 |
| Otavi | 0.033 | 0.047 | 320 | -0.060 | 0.125 | 0.695 | 0.488 |
| Stampriet | 0.138 | 0.055 | 320 | 0.029 | 0.247 | 2.494 | 0.013 |

## Table S9: Best model summary on CTmax data

Best model summary output from analyses of CTmax data of *S. dumicola* spiders. The analysis was based on grand mean centered treatment data (acclimation temperature).

Linear model: CTmax ~ Population * Treatment + Body mass + Time since feeding + Preacclimation duration.

|  | **CTmax – heat tolerance** | | | | |
| --- | --- | --- | --- | --- | --- |
| *Predictors* | *Estimates* | *std. Error* | *CI* | *Statistic* | *p* |
| (Intercept) | 50.42 ^***^ | 0.13 | 50.17 – 50.67 | 397.71 | **<0.001** |
| Population [Karasburg] | -0.29 ^***^ | 0.09 | -0.46 – -0.13 | -3.42 | **0.001** |
| Population [Otavi] | 0.07 | 0.08 | -0.08 – 0.22 | 0.92 | 0.358 |
| Population [Stampriet] | -0.18 | 0.10 | -0.36 – 0.01 | -1.85 | 0.065 |
| Treatment | 0.10 ^***^ | 0.01 | 0.08 – 0.12 | 10.06 | **<0.001** |
| Body mass | -0.01 ^*^ | 0.01 | -0.02 – -0.00 | -1.97 | **0.050** |
| Time since feeding | 0.05 ^*^ | 0.02 | 0.00 – 0.10 | 2.09 | **0.037** |
| Preacclimation duration | 0.00 ^*^ | 0.00 | 0.00 – 0.01 | 2.24 | **0.026** |
| Population [Karasburg] * Treatment | -0.08 ^***^ | 0.02 | -0.11 – -0.05 | -5.35 | **<0.001** |
| Population [Otavi] * Treatment | -0.02 | 0.01 | -0.05 – 0.01 | -1.28 | 0.202 |
| Population [Stampriet] * Treatment | -0.06 ^***^ | 0.02 | -0.09 – -0.03 | -4.07 | **<0.001** |
| Observations | 309 | | | | |
| R^2^ / R^2^ adjusted | 0.409 / 0.389 | | | | |
| AIC | 407.455 | | | | |
| F-statistic | 20.61 on 10 and 298 DF | | | | |
| p-value | < 2.2e-16 | | | | |
| ** p < 0.05   ** p < 0.01   *** p < 0.001* | | | | | |

## Table S10: ANOVA output from CTmax model

ANOVA output from the best model on CTmax of *S. dumicola* spiders. The analysis was based on grand mean centered treatment data (acclimation temperature).

Linear model: CTmax ~ Population * Treatment + Body mass + Time since feeding + preacclimation duration.

| **ANOVA on CTmax model** | | | | | | |
| --- | --- | --- | --- | --- | --- | --- |
| *Row* | *Df* | *Sum Sq* | *Mean Sq* | *F value* | *Pr(>F)* |  |
| Population | 3 | 3.82 | 1.27 | 6.07 | 0.00 | *** |
| Treatment | 1 | 27.67 | 27.67 | 131.78 | 0.00 | *** |
| Body mass | 1 | 1.81 | 1.81 | 8.63 | 0.00 | ** |
| Time since feeding | 1 | 1.13 | 1.13 | 5.40 | 0.02 | * |
| Preacclimation duration | 1 | 1.09 | 1.09 | 5.19 | 0.02 | * |
| Population:Treatment | 3 | 7.74 | 2.58 | 12.29 | 0.00 | *** |
| Residuals | 298 | 62.58 | 0.21 |  |  |  |

** p < 0.05   ** p < 0.01   *** p < 0.001*

## Table S11: Test output on whether CTmax population trends differ

Table showing the output from the emtrends() function on the CTmax model, testing whether trends are different from each other. Significance groups have been added by the MultComp cld() function.

| **CTmax - Trendlines significance groups** | | | | | | |
| --- | --- | --- | --- | --- | --- | --- |
| *Population* | *Treatment.trend* | *SE* | *df* | *lower.CL* | *upper.CL* | *group* |
| Karasburg | 0.022 | 0.013 | 298 | -0.004 | 0.048 | a |
| Stampriet | 0.042 | 0.013 | 298 | 0.017 | 0.066 | a |
| Otavi | 0.086 | 0.011 | 298 | 0.064 | 0.108 | b |
| Betta | 0.104 | 0.010 | 298 | 0.084 | 0.124 | b |

## Table S12: Model summary output for CCRTemp model

Best model summary output from analyses of CCRTemp data of *S. dumicola* spiders. The analysis was based on grand mean centered treatment data (acclimation temperature).

Linear model: CCRTemp ~ Population * Treatment + Body mass mean + Time since feeding.

|  | **CCRTemp – cold tolerance** | | | | |
| --- | --- | --- | --- | --- | --- |
| *Predictors* | *Estimates* | *std. Error* | *CI* | *Statistic* | *p* |
| (Intercept) | 12.93 ^***^ | 0.54 | 11.86 – 13.99 | 23.78 | **<0.001** |
| Population [Karasburg] | -0.83 ^*^ | 0.35 | -1.53 – -0.13 | -2.33 | **0.020** |
| Population [Otavi] | 0.58 | 0.33 | -0.06 – 1.23 | 1.79 | 0.075 |
| Population [Stampriet] | -0.50 | 0.34 | -1.16 – 0.17 | -1.47 | 0.141 |
| Treatment | 0.03 | 0.05 | -0.07 – 0.12 | 0.59 | 0.557 |
| Body mass mean | -0.04 | 0.03 | -0.10 – 0.01 | -1.50 | 0.134 |
| Feding time | -0.31 ^**^ | 0.11 | -0.52 – -0.10 | -2.93 | **0.004** |
| Population [Karasburg] * Treatment | 0.17 ^*^ | 0.07 | 0.04 – 0.31 | 2.50 | **0.013** |
| Population [Otavi] * Treatment | 0.00 | 0.06 | -0.11 – 0.12 | 0.08 | 0.935 |
| Population [Stampriet] * Treatment | 0.11 | 0.07 | -0.02 – 0.24 | 1.62 | 0.105 |
| Observations | 330 | | | | |
| R^2^ / R^2^ adjusted | 0.098 / 0.073 | | | | |
| AIC | 1427.653 | | | | |
| F-statistic | 3.862 on 9 and 320 DF | | | | |
| p-value | 0.0001159 | | | | |
| ** p < 0.05   ** p < 0.01   *** p < 0.001* | | | | | |

## Table S13: ANOVA output from CCRTemp model

ANOVA output from the best model on CCRTemp of *S. dumicola* spiders. The analysis was based on grand mean centered treatment data (acclimation temperature).

Linear model: CCRTemp ~ Population * Treatment + Body mass + Time since feeding.

| **ANOVA on CCRTemp model** | | | | | | |
| --- | --- | --- | --- | --- | --- | --- |
| *Row* | *Df* | *Sum Sq* | *Mean Sq* | *F value* | *Pr(>F)* |  |
| Population | 3 | 48.43 | 16.14 | 3.78 | 0.01 | * |
| Treatment | 1 | 22.64 | 22.64 | 5.30 | 0.02 | * |
| Body mass | 1 | 2.77 | 2.77 | 0.65 | 0.42 |  |
| Time since feeding | 1 | 36.79 | 36.79 | 8.61 | 0.00 | ** |
| Population:Treatment | 3 | 37.91 | 12.64 | 2.96 | 0.03 | * |
| Residuals | 320 | 1367.54 | 4.27 |  |  |  |

** p < 0.05   ** p < 0.01   *** p < 0.001*

## Table S14: Test output on whether CCRTemp population trends differ

Table showing the output from the emtrends() function on the CCRTemp model, testing whether trends are different from each other. Significance groups have been added by the MultComp cld() function.

| **CCRTemp - Trendlines significance groups** | | | | | | |
| --- | --- | --- | --- | --- | --- | --- |
| *Population* | *Treatment.trend* | *SE* | *df* | *lower.CL* | *upper.CL* | *group* |
| Betta | 0.028 | 0.047 | 320 | -0.065 | 0.121 | a |
| Otavi | 0.033 | 0.047 | 320 | -0.060 | 0.125 | a |
| Stampriet | 0.138 | 0.055 | 320 | 0.029 | 0.247 | a |
| Karasburg | 0.201 | 0.059 | 320 | 0.085 | 0.316 | a |

## Table S15: Gene ontology enrichment analysis results

Functional enrichment results of genes expressed with similar population-dependent responses as was found in temperature tolerances (CTmax and CCRTemp). Ontology types investigated: Biological Process (BP) and Molecular Function (MF).

| Ontology type | Data type | GOBPID | Pvalue | OddsRatio | ExpCount | Count | Size | Term |
| --- | --- | --- | --- | --- | --- | --- | --- | --- |
| BP | CCR | GO:0007369 | 0.013958 | 165.3333 | 0.014 | 1 | 2 | gastrulation |
| BP | CCR | GO:0001704 | 0.013958 | 165.3333 | 0.014 | 1 | 2 | formation of primary germ layer |
| BP | CCR | GO:0048869 | 0.013958 | 165.3333 | 0.014 | 1 | 2 | cellular developmental process |
| BP | CCR | GO:0030154 | 0.013958 | 165.3333 | 0.014 | 1 | 2 | cell differentiation |
| BP | CCR | GO:0000002 | 0.020874 | 82.58333 | 0.021 | 1 | 3 | mitochondrial genome maintenance |
| BP | CCR | GO:0048598 | 0.041374 | 32.93333 | 0.042 | 1 | 6 | embryonic morphogenesis |
| BP | CCR | GO:0043549 | 0.041374 | 32.93333 | 0.042 | 1 | 6 | regulation of kinase activity |
| BP | CCR | GO:1904029 | 0.041374 | 32.93333 | 0.042 | 1 | 6 | regulation of cyclin-dependent protein kinase activity |
| BP | CCR | GO:0000079 | 0.041374 | 32.93333 | 0.042 | 1 | 6 | regulation of cyclin-dependent protein serine/threonine kinase activity |
| BP | CCR | GO:0051338 | 0.041374 | 32.93333 | 0.042 | 1 | 6 | regulation of transferase activity |
| BP | CCR | GO:0050790 | 0.041374 | 32.93333 | 0.042 | 1 | 6 | regulation of catalytic activity |
| BP | CCR | GO:0065009 | 0.041374 | 32.93333 | 0.042 | 1 | 6 | regulation of molecular function |
| BP | CCR | GO:0071900 | 0.041374 | 32.93333 | 0.042 | 1 | 6 | regulation of protein serine/threonine kinase activity |
| BP | CCR | GO:0045859 | 0.041374 | 32.93333 | 0.042 | 1 | 6 | regulation of protein kinase activity |
| BP | CTmax | GO:0044770 | 0.00422 | 7.539683 | 0.704 | 4 | 22 | cell cycle phase transition |
| BP | CTmax | GO:0044772 | 0.005388 | 11.02299 | 0.384 | 3 | 12 | mitotic cell cycle phase transition |
| BP | CTmax | GO:0022402 | 0.011693 | 5.388571 | 0.928 | 4 | 29 | cell cycle process |
| BP | CTmax | GO:1903047 | 0.020364 | 6.155172 | 0.608 | 3 | 19 | mitotic cell cycle process |
| BP | CTmax | GO:0048523 | 0.021385 | 3.205917 | 2.272 | 6 | 71 | negative regulation of cellular process |
| BP | CTmax | GO:0044843 | 0.024638 | 10.68889 | 0.256 | 2 | 8 | cell cycle G1/S phase transition |
| BP | CTmax | GO:0000082 | 0.024638 | 10.68889 | 0.256 | 2 | 8 | G1/S transition of mitotic cell cycle |
| BP | CTmax | GO:0070647 | 0.031049 | 9.152381 | 0.288 | 2 | 9 | protein modification by small protein conjugation or removal |
| BP | CTmax | GO:0007049 | 0.038025 | 3.594595 | 1.312 | 4 | 41 | cell cycle |
| BP | CTmax | GO:2000113 | 0.04019 | 3.015873 | 1.952 | 5 | 61 | negative regulation of cellular macromolecule biosynthetic process |
| BP | CTmax | GO:0010558 | 0.04019 | 3.015873 | 1.952 | 5 | 61 | negative regulation of macromolecule biosynthetic process |
| BP | CTmax | GO:0051172 | 0.04019 | 3.015873 | 1.952 | 5 | 61 | negative regulation of nitrogen compound metabolic process |
| BP | CTmax | GO:0031324 | 0.04019 | 3.015873 | 1.952 | 5 | 61 | negative regulation of cellular metabolic process |
| BP | CTmax | GO:0031327 | 0.04019 | 3.015873 | 1.952 | 5 | 61 | negative regulation of cellular biosynthetic process |
| BP | CTmax | GO:0009890 | 0.04019 | 3.015873 | 1.952 | 5 | 61 | negative regulation of biosynthetic process |
| BP | CTmax | GO:0000278 | 0.042428 | 4.448276 | 0.8 | 3 | 25 | mitotic cell cycle |
| BP | CTmax | GO:0044260 | 0.044224 | 2.457778 | 3.392 | 7 | 106 | cellular macromolecule metabolic process |
| BP | CTmax | GO:0009889 | 0.045356 | 2.905492 | 2.016 | 5 | 63 | regulation of biosynthetic process |
| BP | CTmax | GO:2000112 | 0.045356 | 2.905492 | 2.016 | 5 | 63 | regulation of cellular macromolecule biosynthetic process |
| BP | CTmax | GO:0010556 | 0.045356 | 2.905492 | 2.016 | 5 | 63 | regulation of macromolecule biosynthetic process |
| BP | CTmax | GO:0031326 | 0.045356 | 2.905492 | 2.016 | 5 | 63 | regulation of cellular biosynthetic process |
| MF | CTmax | GO:0000981 | 0.010249 | 4.111559 | 2.752294 | 7 | 100 | DNA-binding transcription factor activity, RNA polymerase II-specific |
| MF | CTmax | GO:0003700 | 0.010249 | 4.111559 | 2.752294 | 7 | 100 | DNA-binding transcription factor activity |
| MF | CTmax | GO:0140110 | 0.010249 | 4.111559 | 2.752294 | 7 | 100 | transcription regulator activity |

## Supplementary comment 1: Potential confounding factors:

The spiders from all four populations were sampled over a relatively short period, and due to population differences they do not show entirely synchronized phenology. We made an effort to assay spiders in a size-dependent order, nevertheless they exhibited differences in body mass when their thermal limits were estimated (ANOVA, p-value < 2e-16 in all acclimation regimes in both CTmax and CCRTemp). The spiders from Otawi were the heaviest, followed by spiders from Karasburg, Betta, and Stampriet (Figure S5). In order to test if the body mass difference among populations represents a potential confounding factor regarding their thermal tolerances (Anthony et al., 2021; Oyen et al., 2021), we correlated individual CTmax and CCRTemp with body masses for each population separately. In most tests there were non-significant correlations, and no common trend was observed. On the contrary, sometimes there is a negative trend and sometimes a positive trend.

We included time since feeding, since feeding – for practical reasons – was done on specific days for all spiders, and time since feeding could influence temperature tolerances (Manenti et al., 2018; Nyamukondiwa & Terblanche, 2009). We also included the number of days the spiders had spent in the lab at 21˚C before being separated out into acclimation treatments (Preacclimation duration). However, note that time since feeding and preacclimation duration is not biased among populations. In summary, the size of the spiders, their hunger state, and preacclimation duration, did not seem to influence their thermal tolerances in a biased way.

## References for supplement:

Anthony, S. E., Buddle, C. M., Høye, T. T., Hein, N., & Sinclair, B. J. (2021). Thermal acclimation has limited effect on the thermal tolerances of summer-collected Arctic and sub-Arctic wolf spiders. *Comparative Biochemistry and Physiology Part A: Molecular & Integrative Physiology*, *257*, 110974. doi: 10.1016/J.CBPA.2021.110974

Busck, M. M., Settepani, V., Bechsgaard, J., Lund, M. B., Bilde, T., & Schramm, A. (2020). Microbiomes and Specific Symbionts of Social Spiders: Compositional Patterns in Host Species, Populations, and Nests. *Frontiers in Microbiology*, *11*, 1–14. doi: 10.3389/fmicb.2020.01845

Liu, S., Aagaard, A., Bechsgaard, J., & Bilde, T. (2019). DNA methylation patterns in the social spider, *Stegodyphus dumicola*. *Genes*, *10*(2), 1–17. doi: 10.3390/genes10020137

Manenti, T., Cunha, T. R., Sørensen, J. G., & Loeschcke, V. (2018). How much starvation, desiccation and oxygen depletion can *Drosophila melanogaster* tolerate before its upper thermal limits are affected? *Journal of Insect Physiology*, *111*, 1–7. doi: 10.1016/J.JINSPHYS.2018.09.002

Nyamukondiwa, C., & Terblanche, J. S. (2009). Thermal tolerance in adult Mediterranean and Natal fruit flies (*Ceratitis capitata* and *Ceratitis rosa*): Effects of age, gender and feeding status. *Journal of Thermal Biology*, *34*(8), 406–414. doi: 10.1016/J.JTHERBIO.2009.09.002

Oyen, K. J., Jardine, L. E., Parsons, Z. M., Herndon, J. D., Strange, J. P., Lozier, J. D., & Dillon, M. E. (2021). Body mass and sex, not local climate, drive differences in chill coma recovery times in common garden reared bumble bees. *Journal of Comparative Physiology B: Biochemical, Systemic, and Environmental Physiology*, *191*(5), 843–854. doi: 10.1007/s00360-021-01385-7

Sumner, L. W., Amberg, A., Barrett, D., Beale, M. H., Beger, R., Daykin, C. A., … Viant, M. R. (2007). Proposed minimum reporting standards for chemical analysis: Chemical Analysis Working Group (CAWG) Metabolomics Standards Initiative (MSI). *Metabolomics*, *3*(3), 211–221. doi: 10.1007/S11306-007-0082-2/METRICS
